# Protein-based nanoparticles for antimicrobial and cancer therapy: implications for public health

**DOI:** 10.1039/d5ra01427a

**Published:** 2025-05-08

**Authors:** Ikhazuagbe Hilary Ifijen, Raymond Femi Awoyemi, Emmanuel Faderin, Uchenna Uzoma Akobundu, Abiola Samuel Ajayi, Janefrances U. Chukwu, Ogunnaike Korede Lekan, Olutoyin Deborah Asiriuwa, Muniratu Maliki, Esther Uwidia Ikhuoria

**Affiliations:** a Department of Research Outreach, Rubber Research Institute of Nigeria Iyanomo, PMB 1049 Benin City Nigeria larylans4u@yahoo.com ifijen.hilary@rrin.gov.ng; b Department of Chemistry, Mississippi State University Starkville Mississippi MS 39762 United State of America; c Department of Pharmaceutical Sciences, Southern Illinois University, Edwardsville 1 Hairpin Drive Edwardsville IL 62026-001 USA; d University of Tennessee 1000 Volunteer BLVD Knoxville TN 37916 USA; e Texas Southern University 3100 Cleburne St Houston TX 77004 USA; f Department of Chemistry, West Virginia University Morgantown WV 26506 USA; g Department of Chemistry, Wichita State University 1845 Fairmount, Box 150 Wichita KS 67260-0150 USA; h Department of Industrial Chemistry, Edo State University Iyamho Edo State Nigeria; i Department of Chemistry, University of Benin Benin City Edo State Nigeria

## Abstract

This review discusses the growing potential of protein-based nanoparticles (PBNPs) in antimicrobial and cancer therapies, emphasizing their mechanisms of action, applications, and future prospects. In antimicrobial therapy, PBNPs exhibit several mechanisms of action, including disruption of microbial membranes, enhanced antibiotic delivery, immune modulation, and biofilm disruption. Protein nanoparticles like albumin, lactoferrin, gelatin, and peptide-based variants enhance the efficacy of antibiotics, offering targeted approaches to combat multidrug-resistant pathogens. Their ability to improve drug localization and enhance microbial eradication represents a significant advancement in infectious disease management. In cancer therapy, PBNPs facilitate targeted drug delivery, controlled release, tumor microenvironment modulation, and photothermal and photodynamic therapies. Nanoparticles such as Abraxane® and engineered ferritin nanocages are at the forefront of cancer treatment, enhancing the precision and effectiveness of chemotherapy while minimizing adverse effects. Additionally, silk fibroin nanoparticles are being explored for their biodegradability and targeting capabilities. Despite their promise, challenges remain, including the scalability of production, long-term safety concerns, regulatory approval processes, and environmental impact. Addressing these issues through rigorous research and innovation is crucial for integrating PBNPs into mainstream therapeutic practices. PBNPs offer transformative solutions in both antimicrobial and cancer therapies, with significant implications for improving public health outcomes globally.

## Abstract 2

This review discusses the growing potential of protein-based nanoparticles (PBNPs) in antimicrobial and cancer therapies, emphasizing their mechanisms of action, applications, and future prospects.

## Graphical abstract

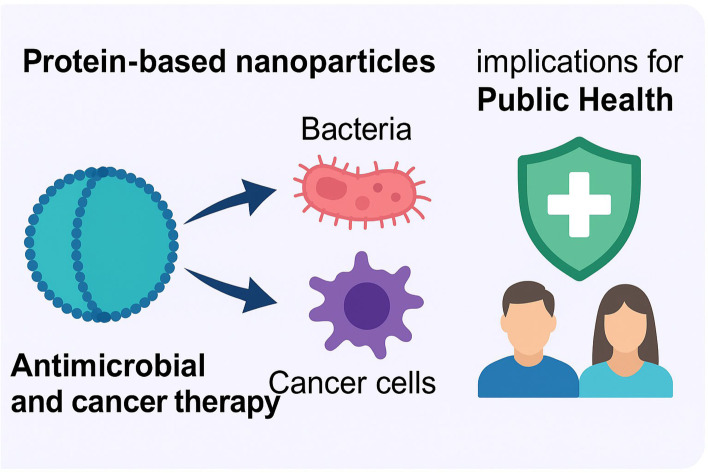

## 1 Introduction

The growing challenges posed by antimicrobial resistance and the global burden of cancer have emerged as critical threats to public health, demanding urgent and innovative solutions.^1–5^ Antimicrobial resistance is escalating at an alarming rate, rendering many conventional therapies ineffective against multidrug-resistant pathogens. This phenomenon has severe implications, as infections that were once easily treatable now result in prolonged illnesses, increased mortality rates, and rising healthcare costs. Similarly, cancer remains a leading cause of death worldwide, with its treatment hindered by significant obstacles such as systemic toxicity, the emergence of drug resistance, and the lack of specificity in targeting cancer cells.^6–10^ Together, these issues highlight a pressing need for novel therapeutic strategies capable of overcoming these limitations and improving patient outcomes.

Recent advances in nanotechnology have paved the way for groundbreaking innovations in medicine, offering the ability to engineer materials at the molecular and nanoscale levels.^11,12^ This precision has opened new horizons in drug delivery and therapeutic applications, enabling the development of materials that can effectively address the shortcomings of conventional treatments. Among these advancements, protein-based nanoparticles (PBNPs) have garnered significant attention due to their unique combination of favourable characteristics.^13,14^ Derived from naturally occurring proteins such as albumin, ferritin, silk fibroin, and gelatin, PBNPs exhibit exceptional biocompatibility and biodegradability, making them safe for use in biological systems. Moreover, their natural origin allows for functional modifications that enhance their performance in a variety of biomedical applications.^15,16^

PBNPs are particularly well-suited for therapeutic applications due to their ability to encapsulate a wide range of therapeutic agents, including small molecules, proteins, and nucleic acids. This encapsulation not only protects the therapeutic agents from premature degradation but also enhances their stability and bioavailability. Additionally, PBNPs can be engineered to deliver drugs selectively to diseased sites, minimizing systemic exposure and reducing off-target effects. These properties make PBNPs an attractive platform for addressing the challenges of both antimicrobial resistance and cancer treatment.^17–19^

The functional adaptability of PBNPs further enhances their potential in biomedical applications. Surface modifications can be incorporated into PBNPs to achieve targeted delivery, enabling them to home in on specific tissues or pathogens. These modifications can also extend the circulation time of the nanoparticles in the bloodstream, increasing their therapeutic efficacy. In addition, PBNPs can be designed to exhibit stimuli-responsive behaviour, allowing them to release their therapeutic payload in response to specific conditions, such as changes in pH, temperature, or enzyme activity, commonly found in pathological environments.^20,21^

In the realm of antimicrobial therapy, PBNPs offer several advantages that address critical challenges posed by multidrug-resistant pathogens. They can improve the stability and solubility of encapsulated antimicrobial agents, enabling them to maintain their activity for longer durations. PBNPs are also capable of disrupting biofilms, which are protective barriers formed by microbial communities that contribute to resistance mechanisms. Furthermore, PBNPs can interact with microbial membranes, increasing the permeability of these membranes and enhancing the efficacy of the encapsulated drugs. These properties position PBNPs as a promising tool for combating antimicrobial resistance and restoring the effectiveness of existing antimicrobial therapies.^22,23^

In cancer therapy, PBNPs provide transformative potential by addressing key limitations of traditional chemotherapeutic approaches. Their ability to deliver drugs precisely to tumor sites reduces systemic toxicity and enhances the therapeutic index of chemotherapeutics. PBNPs can be engineered to release their payload in a controlled manner, ensuring sustained drug delivery over time and reducing the frequency of dosing. Additionally, PBNPs offer multifunctional capabilities, enabling the integration of therapeutic and diagnostic functions within a single platform. This dual functionality, often referred to as theranostics, allows for real-time monitoring of treatment efficacy and precise adjustments to therapy as needed.^24,25^

This review examines the antimicrobial and anticancer applications of PBNPs, focusing on their mechanisms of action, therapeutic advantages, and potential to tackle critical public health challenges. By delivering targeted, efficient, and safe treatment options, PBNPs present a promising pathway for advancing the management of multidrug-resistant infections and cancer. Their remarkable versatility and adaptability highlight their transformative potential, inspiring optimism for addressing the shortcomings of traditional therapies and enhancing global health outcomes.

## 2 Protein-based nanoparticles in antimicrobial therapy

### 2.1 Mechanisms of action

#### 2.1.1 Mechanisms of action of protein-based nanoparticles (PBNPs) in combating microbial infections

Protein-based nanoparticles (PBNPs) employ multiple mechanisms to address microbial infections effectively. These mechanisms leverage the intrinsic properties of the proteins used in their construction, as well as the functionalization and encapsulation strategies applied during their synthesis.^26,27^

##### 2.1.1.1 Disruption of microbial membranes

PBNPs functionalized with antimicrobial peptides or metallic agents exhibit potent membrane-disruptive activity, a critical mechanism underlying their antimicrobial efficacy. Antimicrobial peptides (AMPs) integrated into PBNPs can selectively target microbial cell membranes due to the electrostatic attraction between the positively charged peptides and the negatively charged components of bacterial surfaces, such as lipopolysaccharides in Gram-negative bacteria and teichoic acids in Gram-positive bacteria. This selective binding destabilizes the lipid bilayer, causing structural disorganization, membrane permeabilization, and leakage of essential intracellular components, ultimately resulting in cell death.^28,29^

Similarly, PBNPs functionalized with metallic agents, such as silver ions or zinc oxide nanoparticles, act synergistically to enhance antimicrobial potency. These metallic agents catalyze the production of reactive oxygen species (ROS), which oxidatively damage membrane lipids and proteins. This oxidative stress compromises the membrane's integrity and further disrupts cellular processes. Moreover, the ROS-mediated damage can extend beyond the membrane, affecting intracellular components such as DNA and enzymes, amplifying the antimicrobial effect.^30,31^

The combination of these mechanisms—AMP-mediated disruption and ROS generation by metallic agents—ensures broad-spectrum efficacy of PBNPs against a variety of pathogens, including Gram-positive and Gram-negative bacteria as well as fungal species. This dual-action strategy also reduces the likelihood of resistance development, making PBNPs a promising candidate for combating multidrug-resistant infections.

As highlighted by Park *et al.* (2022), AMPs selectively bind to bacterial surfaces, exploiting the negative charge of lipopolysaccharides and teichoic acids.^32^ This selective targeting is enhanced by the amphipathic structure of AMPs, which facilitates membrane insertion and destabilization through mechanisms such as the toroidal pore, barrel-stave, and carpet models. Upon binding, these peptides induce structural disorganization, leading to membrane permeabilization, leakage of intracellular contents, and microbial cell death.

**Fig. 1:**
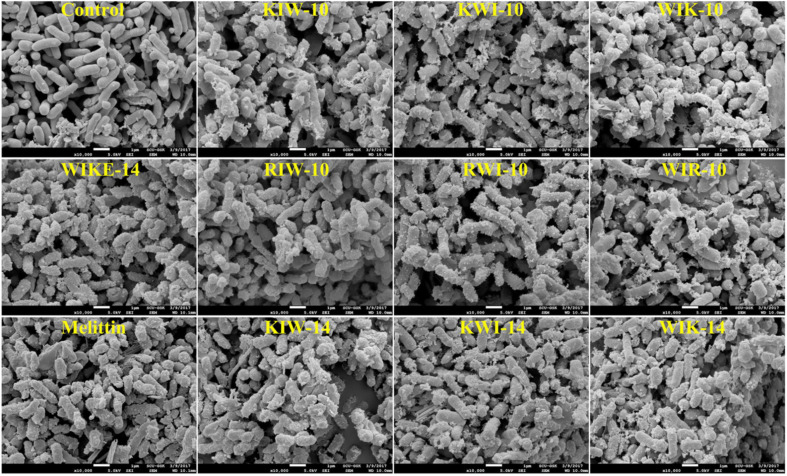
Peptide-induced morphological changes in *E. coli* cells. All peptides were treated at minimum inhibitory concentrations (MICs).^32^

Park *et al.* (2022) demonstrated that the antibacterial efficacy of AMPs depends on peptide length, sequence composition, and positional arrangement of amino acids such as tryptophan, lysine, and arginine.^32^ Peptides with 14 amino acids exhibited superior antibacterial activity compared to their 10-amino acid counterparts, particularly when tryptophan was positioned near the N-terminus. This arrangement increased the peptide's binding affinity to bacterial membranes and enhanced its membrane-disruptive effects. The study also revealed the importance of balancing cationic charge and amphiphilicity to optimize both efficacy and selectivity. In Fig. 1, the interaction of AMPs with bacterial membranes illustrates their ability to induce pore formation and subsequent structural collapse, emphasizing the potential of these peptides to combat drug-resistant strains.

**Fig. 2:**
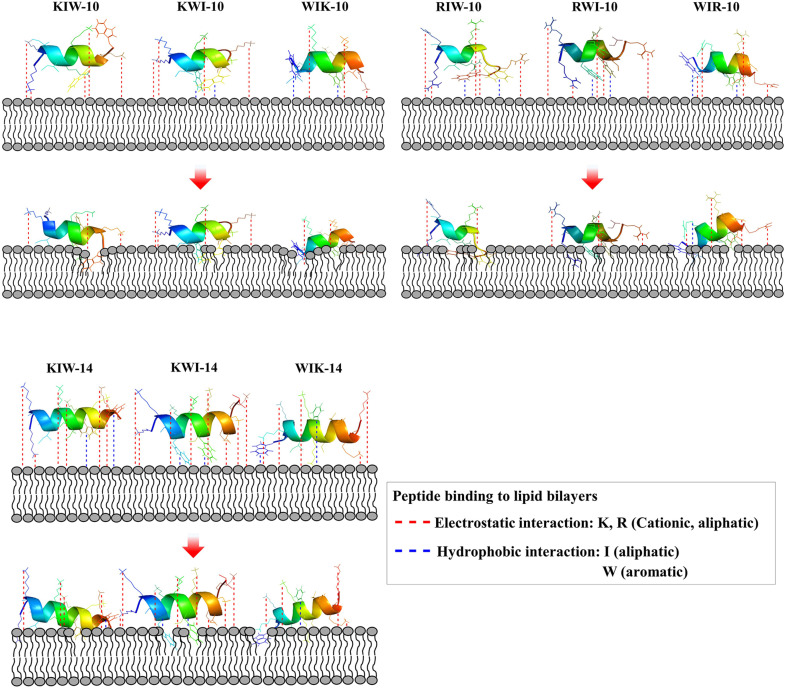
Schematic mode of action of the designed peptides in bacterial membranes. Red-dotted lines indicate electrostatic interaction between cationic amino acids and anionic head group of lipids. Hydrophobic interaction between hydrophobic amino acids and fatty acids of lipids. Hydrophobic interaction between hydrophobic amino acids and fatty acids of lipids.^32^

Similarly, PBNPs containing metallic agents such as silver ions provide a complementary mechanism through the generation of reactive oxygen species (ROS). These ROS compromise membrane integrity by oxidizing lipid bilayers, proteins, and other cellular components, leading to cellular dysfunction and death. The dual functionality of AMPs and metallic agents within PBNPs ensures their broad-spectrum efficacy against Gram-positive and Gram-negative bacteria, as well as fungal pathogens. Fig. 2 visually encapsulates the ROS-mediated damage and membrane disruption caused by metallic agents, highlighting their role in enhancing the antimicrobial efficacy of PBNPs.

The integration of these mechanisms into a single platform offers a promising alternative to conventional antibiotics, particularly in the context of drug-resistant pathogens. Unlike traditional antibiotics, which often target specific metabolic pathways, the physical and chemical disruptions induced by PBNPs are less likely to engender resistance. The findings of Park *et al.* (2022) provide critical insights into the rational design of AMPs and their integration into nanomaterials, paving the way for next-generation antimicrobial therapeutics.^32^

Furthermore, the cytotoxicity studies presented by Park *et al.* (2022) underline the importance of ensuring that AMPs are non-toxic to mammalian cells.^32^ Despite their potent activity, the peptides displayed minimal hemolytic and cytotoxic effects on human cells, as shown in Fig. 1. This selectivity underscores their potential as safe and effective alternatives to traditional antibiotics. In conclusion, the combination of AMP-mediated membrane disruption and ROS generation by metallic agents within PBNPs offers a robust strategy to address the growing challenge of antimicrobial resistance while maintaining safety profiles suitable for clinical applications.

The study by Zharkova *et al.* (2022) investigates the membrane-disruptive activity of silver nanoparticles (AgNPs) functionalized with antimicrobial peptides (AMPs) or small antimicrobial proteins (APs).^33^ This combination of AgNPs and AMPs/APs holds promise as an effective therapeutic approach for tackling the increasing number of drug-resistant pathogens. The study explores the synergy between these two platforms, focusing on how the functionalization of AgNPs with AMPs enhances their antimicrobial properties.

**Fig. 3:**
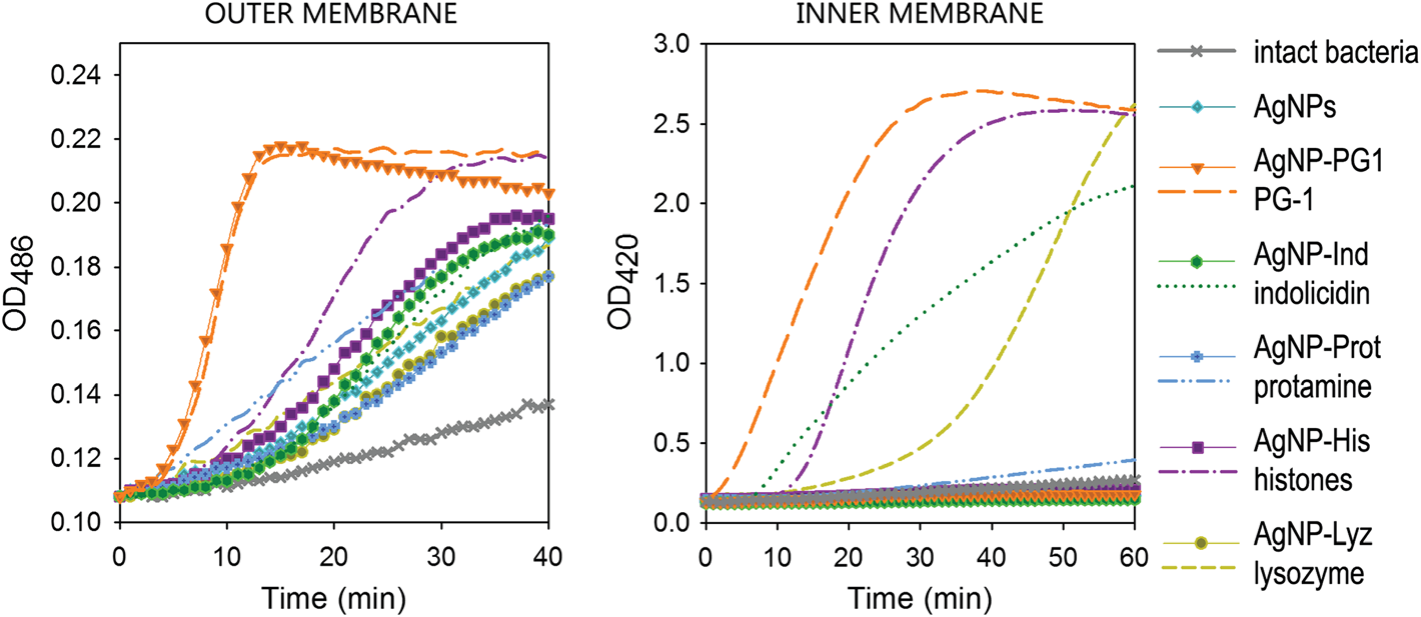
Permeabilizing effect of AgNP–AMP/AP conjugates on *Escherichia coli* ML-35p outer and inner membranes. The effects of the conjugates are compared to those of the corresponding antimicrobial peptides and proteins (AMPs and APs) and gelatin-only coated silver nanoparticles (AgNPs) alone. The optical density (OD) increase correlates to the hydrolysis of the chromogenic markers by bacterial enzymes. Permeabilization of the outer membrane gives the nitrocefin marker the access to periplasmic β-lactamase, while inner membrane permeabilization gives the *o*-nitrophenyl-β-d-galactoside marker (ONPG) the access to cytoplasmic β-galactosidase. Bacterial membranes are impenetrable to the markers under normal conditions; hence the dynamics of their degradation allow assessing the scale and velocity of membrane damage inflicted by the tested substances. The concentration of antimicrobials used was equal to 4 × MIC (minimal inhibitory concentration); typical curves are shown.^33^

A key finding of this study is the enhanced ability of AgNP–AMP/AP conjugates to disrupt bacterial membranes, a crucial mechanism in the antimicrobial action of AMPs. The results, shown in Fig. 3, demonstrate that the conjugates significantly increase the permeability of the *Escherichia coli* (*E. coli*) ML-35p outer membrane (panel A), with varying kinetics depending on the conjugate used. The optical density (OD) increase observed in these assays correlates with the hydrolysis of the chromogenic markers, nitrocefin and ONPG, which are used to assess membrane permeabilization. The nitrocefin marker accesses periplasmic β-lactamase upon outer membrane permeabilization, while ONPG is used to assess cytoplasmic membrane permeability by enabling access to β-galactosidase. These dynamics clearly demonstrate the membrane-disruptive potential of the AgNP–AMP/AP conjugates compared to free AMPs/APs and gelatin-only coated AgNPs.

Notably, the highly lytic protegrin-1 (PG-1), when conjugated to AgNPs, showed a membrane permeabilization pattern very similar to its free form. This is evident from the sharp rise and abrupt transition to a plateau in the outer membrane permeabilization curve (Fig. 3A), characteristic of PG-1's membranolytic activity. This suggests that PG-1 retains its potent membrane-disruptive capacity even when bound to AgNPs, highlighting that its rigid structure does not interfere with its activity.

However, the study also observed a marked reduction in the ability of AgNP–AMP/AP conjugates to permeabilize the inner bacterial membrane, as shown in Fig. 3B. The kinetics and extent of inner membrane permeabilization were significantly less for the conjugates compared to the free AMPs, with none of the conjugates causing substantial membrane damage to the cytoplasmic membrane. This finding is consistent with the observation that the AgNP–AMP/AP conjugates did not acquire the ability to disrupt the inner membrane in the same way as the free peptides.

Furthermore, the study found that functionalizing AgNPs with AMPs/APs reduced the hemolytic toxicity of the free peptides. All AgNP–AMP/AP conjugates exhibited low hemolytic activity toward human erythrocytes, which suggests that conjugation may mitigate the toxicity typically associated with membranolytic peptides. This property makes AgNP–AMP/AP conjugates a promising strategy not only for enhancing antimicrobial efficacy but also for reducing the risk of side effects in therapeutic applications.

In addition to their antibacterial properties, the functionalization of AgNPs with AMPs/APs may impart additional therapeutic benefits. The study suggests that AMPs/APs could also transfer immunomodulatory and wound-healing effects to the nanoparticles, enhancing their potential for broader clinical applications. Some conjugated AgNPs even showed selective toxicity toward tumor cells, though the authors caution that not all immunomodulatory effects will necessarily be beneficial to normal cells.

The study by Zharkova *et al.* (2022) provides compelling evidence for the potent membrane-disruptive activity of AgNPs functionalized with AMPs/Aps.^33^Fig. 3 highlights the differential effects of these conjugates on the outer and inner bacterial membranes, with AgNP–AMP/AP conjugates enhancing outer membrane permeability while showing reduced activity against the cytoplasmic membrane. This combination of antimicrobial potency and reduced toxicity underscores the potential of AgNP–AMP/AP conjugates as a promising platform for combating drug-resistant bacteria and potentially other therapeutic applications, including cancer treatment.

Sha *et al.* (2020) demonstrated how self-assembled peptide nanofibrils can encapsulate antimicrobial peptides, releasing them upon disassembly when exposed to bacteria.^34^ The resulting antimicrobial activity, which involved bacterial membrane disruption and calcium efflux, displayed good long-term stability and low cytotoxicity. This work highlights the potential of peptide-based nanomaterials in developing sustainable antimicrobial agents that balance efficacy with biocompatibility, addressing the stability issues that often plague free peptides.

In line with this, Lam *et al.* (2016) explored a class of peptide polymer nanoparticles, termed SNAPPs, which are star-shaped and engineered to tackle Gram-negative pathogens, including multidrug-resistant strains.^35^ The study revealed that the presence of physiological concentrations of divalent cations could reduce the antimicrobial efficacy of SNAPPs, but this effect could be reversed through chelation. Notably, SNAPPs demonstrated superior membrane-disrupting abilities, causing both outer and inner membrane destabilization in bacteria. This study emphasizes the importance of understanding the interactions between antimicrobial agents and their environments, such as the presence of salts and proteins *in vivo*, which can significantly affect their efficacy.

Further refining the application of AMPs in nanoparticle-based therapeutics, Chen *et al.* (2019) addressed the common issue of AMP toxicity to mammalian cells.^36^ By integrating a natural AMP with a β-sheet-forming synthetic peptide, they developed a self-assembled nanofiber that displayed selective bacterial membrane disruption. The unique arrangement of the AMPs on the nanofiber surface reduced the hydrophobic interactions between the AMP and mammalian cell membranes, leading to preferential bacterial cell membrane disruption. This strategy enhanced the therapeutic potential of AMPs, minimizing their cytotoxicity while retaining effective antimicrobial action. The study's success in controlling the conformation and presentation of AMPs highlights a novel approach to creating more selective antimicrobial therapies.

Chang *et al.* (2017) introduced another innovative approach with self-assembled peptide amphiphiles (PA), functionalized with a heparin-binding Cardin-motif peptide, to combat bacterial resistance.^37^ The self-assembly of these peptides into cylindrical nanostructures significantly improved their antibacterial potency, particularly against Gram-negative bacteria. The ACA-PA exhibited a dose-dependent antibacterial effect, inducing membrane disruption and cytoplasmic leakage in both Gram-positive and Gram-negative bacteria. The study illustrates the versatility of self-assembled peptide nanoparticles in overcoming antibiotic resistance and offers a new direction for non-antibiotic antimicrobial agents.

Wadhwani *et al.* (2017) took a different approach by tethering antimicrobial peptides to gold nanoparticles.^38^ This method not only preserved the biological activity of the peptides but also enhanced their stability against proteolytic degradation. The site-selective functionalization of gold nanoparticles with cationic peptides allowed the peptides to retain their α-helical conformation in the presence of bacterial membranes, thereby maintaining their antimicrobial properties. This work exemplifies how nanoparticle functionalization can improve the longevity and efficacy of antimicrobial peptides, making them more suitable for therapeutic applications.

Zharkova *et al.* (2021) expanded on the synergistic effects of combining silver nanoparticles (AgNPs) with AMPs.^39^ The study found that conjugating small antimicrobial proteins such as protegrin-1 to AgNPs not only enhanced their antibacterial activity but also reduced their toxicity towards eukaryotic cells. The AgNP–AMP conjugates were effective against both Gram-positive and Gram-negative bacteria, including resistant strains, while exhibiting low hemolytic activity. This dual action of nanoparticles and AMPs offers a promising strategy for developing novel antimicrobial agents with minimized side effects. The study also suggested that AgNP–AMP conjugates could have additional benefits, such as immunomodulatory effects and selective toxicity toward tumor cells.

The integration of antimicrobial peptides with protein-based nanoparticles or metallic agents represents a multifaceted strategy to combat bacterial resistance. These systems harness the potent membrane-disruptive capabilities of AMPs while overcoming the limitations of peptide stability and specificity. Whether through self-assembly, conjugation with gold nanoparticles, or the addition of silver ions, the functionalization of PBNPs ensures both antimicrobial efficacy and cytocompatibility. As these technologies advance, they hold great promise for addressing the growing global threat of antimicrobial resistance, offering safer, more effective alternatives to conventional antibiotics.

##### 2.1.1.2 Enhanced antibiotic delivery

PBNPs (polymetallic-based nanoparticles) have gained attention as highly effective carriers for encapsulating and delivering antibiotics, addressing several inherent limitations of conventional antimicrobial therapy. Traditional antibiotics often face challenges such as poor solubility, rapid enzymatic degradation, and short half-lives in the body, which can reduce their therapeutic efficacy.^40,41^ When antibiotics are encapsulated within PBNPs, they are protected from these challenges, improving their stability and bioavailability. PBNPs are particularly valuable for enhancing the solubility of hydrophobic antibiotics, which are otherwise poorly absorbed and often ineffective. By encapsulating these antibiotics in nanoparticles, the solubility and pharmacokinetics are significantly improved, leading to better therapeutic outcomes.^42–44^

Additionally, PBNPs offer protection to the antibiotics they carry, shielding them from enzymatic degradation and preventing premature inactivation. This is particularly important in the context of infections caused by multi-drug-resistant (MDR) pathogens, where antibiotic resistance is frequently exacerbated by enzymatic breakdown of drugs. Encapsulation within nanoparticles ensures that the antibiotic remains intact and active for a longer period, enhancing its overall effectiveness in combating infection.^45,46^

One of the major advantages of PBNPs as antibiotic carriers is their ability to facilitate targeted delivery. Infections often occur in challenging environments, such as biofilms or intracellular infection sites, where pathogens can evade conventional treatments. Biofilms, in particular, are known to provide a protective barrier that makes it difficult for antibiotics to penetrate and eliminate the bacteria. PBNPs, however, can be engineered to target these difficult-to-reach areas, ensuring that the encapsulated antibiotic is delivered precisely to the site of infection. For example, albumin-based nanoparticles loaded with ciprofloxacin have demonstrated significant improvement in penetrating biofilms, enabling the antibiotic to reach the bacteria that are typically shielded from treatment. This targeted delivery not only enhances the antibiotic's efficacy but also minimizes its effects on healthy tissues, reducing the risk of side effects.^47,48^

Furthermore, PBNPs can be designed with controlled release properties, allowing for the sustained release of antibiotics over an extended period. This sustained release ensures that therapeutic levels of the drug are maintained in the body for longer durations, improving treatment outcomes and reducing the frequency of dosing. The controlled release mechanism also reduces the likelihood of antibiotic resistance development, as it prevents the rapid fluctuations in drug concentration that can lead to the selection of resistant bacterial strains. By maintaining a constant therapeutic level of the antibiotic at the infection site, PBNPs provide a more efficient and longer-lasting treatment option.^49,50^

PBNPs represent a promising solution to the limitations of conventional antimicrobial therapies by improving the solubility, stability, and bioavailability of antibiotics, enhancing their ability to target hard-to-reach infection sites, and ensuring a controlled and sustained release. These properties make PBNPs highly effective carriers in the fight against antibiotic-resistant infections and in the development of more efficient and safer antimicrobial therapies.

For example, recent studies have shown significant potential in using nanoparticles as carriers to enhance the delivery of antibiotics, particularly in addressing the challenges posed by bacterial resistance and biofilm formation. Subramaniam *et al.* (2024) highlighted the difficulty in treating persistent intracellular infections due to bacteria's survival mechanisms within host cells. Conventional antibiotics often struggle to penetrate cell membranes, but antibiotic-loaded nanocarriers, such as liposomes and cubosomes, offer a promising solution.^27^ These nanocarriers improve the delivery of antibiotics to intracellular infection sites, enhancing bacterial eradication. However, the effectiveness of these nanoantibiotics is influenced by the formation of a protein corona when they interact with proteins in the biological environment. The study demonstrated that this protein corona can either enhance or hinder the cellular uptake of nanoparticles, depending on the nanoparticle type. Specifically, cubosomes showed improved internalization and bacterial killing ability within macrophages, illustrating the potential of using nanocarriers to improve the treatment of intracellular infections. This finding emphasizes the need for a better understanding of how protein coronas affect nanoparticle performance, which is crucial for translating these nanocarrier systems from *in vitro* to *in vivo* applications.

In a similar vein, Yong *et al.* (2024) explored the potential of polymeric nanoparticles with cationic shell surfaces for sustained antibiotic delivery.^40^ These nanoparticles not only possess intrinsic antimicrobial properties due to their high positive charge density but can also be loaded with biocidal agents like curcumin and terbinafine for dual antibacterial activity. By incorporating these agents into soft core–shell nanoparticles, the system facilitates both immediate bacterial killing upon contact and a sustained therapeutic effect through controlled drug release. This platform showed promising results against both Gram-negative and Gram-positive bacteria, demonstrating how charged nanoparticle coronas can enhance the therapeutic efficacy of antibiotics. The study emphasized the importance of combining direct antibacterial activity with controlled drug delivery to overcome the growing issue of antibiotic resistance, further supporting the potential of nanoparticles in enhancing the effectiveness of antimicrobial treatments.

Ahsan *et al.* (2024) focused on the challenge of biofilm-associated bacterial infections, which are particularly resistant to treatment due to the protective nature of biofilms.^41^ The study discussed how combining antibiotics with antimicrobial adjuvants, such as quorum sensing inhibitors and EPS-degrading enzymes, can enhance the effectiveness of existing treatments. Nanoparticulate systems, particularly lipid nanocarriers (LNCs), have shown great promise in this context, offering superior properties like better biocompatibility, high drug-loading capacity, and controlled drug release. The encapsulation of antibiotics within LNCs ensures better stability and targeted delivery, crucial for overcoming the biofilm barrier. The review highlighted various LNC platforms, such as liquid crystal nanoparticles (LCNPs), liposomes, solid lipid nanoparticles (SLNs), and nanostructured lipid carriers (NLCs), as effective means to co-deliver antibiotics and adjuvants. These systems not only protect the drugs from degradation but also enhance their penetration into biofilms, improving therapeutic outcomes and potentially reducing bacterial resistance. This approach paves the way for more effective antimicrobial treatments, contributing to the fight against antimicrobial resistance.

Together, these studies underscore the potential of nanoparticles as versatile carriers for antibiotic delivery. Their ability to encapsulate and protect antibiotics, improve solubility, and target specific infection sites—especially biofilms and intracellular pathogens—makes them valuable tools in enhancing the efficacy of antimicrobial therapies. By improving drug stability, prolonging systemic circulation, and enabling controlled release, nanoparticles help mitigate the development of resistance and offer promising alternatives to conventional antibiotic treatments. These advancements highlight the growing role of nanotechnology in revolutionizing the treatment of bacterial infections, particularly in the face of rising antimicrobial resistance.

##### 2.1.1.3 Immune system modulation

The modulation of the immune system using engineered materials has emerged as a promising strategy to combat infections, particularly in the context of bacterial infections, where pathogens often adopt immune evasion tactics to ensure their survival.^51^ This challenge has become more pronounced due to the rise of antimicrobial resistance, which limits the effectiveness of traditional antibiotics. As a result, there has been a growing interest in developing host-directed therapies that leverage immunomodulatory biomaterials. These materials aim to enhance the body's innate immune responses, providing a complementary approach to traditional antimicrobial treatments.^52,53^ In this context, biomaterials such as metal ion-releasing coatings, stimuli-responsive polymeric coatings, and interleukin-releasing surfaces have been explored for their ability to modulate immune activity. Additionally, various nanoparticle-based systems, including lipid-based nanoparticles, biomimetic nanoparticles, and inorganic nanocarriers, have shown promise in enhancing immune responses. These materials can not only serve as delivery systems for therapeutic agents but can also act as direct modulators of the immune system. Immunomodulatory hydrogels, particularly those used for wound infection treatment, have also been investigated for their potential to regulate immune cell activity at the site of infection, further enhancing the body's ability to clear pathogens and reduce the incidence of infection recurrence.^51,53^

In another innovative approach, the development of prolonged antigen delivery systems has been explored as a means to induce durable immune responses. This strategy aims to mimic the prolonged exposure to pathogens, which is key for developing lasting immunity. By assembling His-tagged proteins into supramolecular complexes, researchers have created protein-only nanoparticles and microparticles that serve as controlled-release systems for antigens. These materials act similarly to secretory granules in the mammalian hormonal system, progressively releasing the antigen over time, which can significantly enhance the immune response.^54^ In the study by Bosch-Camós *et al.* (2024), such systems were tested using a nanostructured version of the p30 protein, a major immunogen from the African swine fever virus.^54^ The results demonstrated potent pro-inflammatory activity in porcine macrophages and strong humoral and cellular immune responses *in vivo*. This suggests that these dynamic depot materials could serve as effective immunostimulants, potentially eliminating the need for traditional adjuvants and offering a novel approach to vaccine development.

In the context of respiratory infections, the development of self-adjuvanted protein nanoparticles has shown promise in enhancing immune responses against viral pathogens such as influenza. Kim *et al.* (2024) reported the creation of double-layered protein nanoparticles consisting of influenza nucleoprotein cores coated with hemagglutinin and a truncated form of bacterial flagellin.^55^ These nanoparticles, when administered intranasally, significantly amplified both humoral and cellular immune responses, enhancing antigen-specific IgA and IgG levels in mucosal washes and increasing the populations of lung-resident memory B cells. The slow-release strategy employed by splitting the prime dose into multiple applications over an extended period further boosted the immune response, improving protection against both homologous and heterologous influenza viral challenges. This approach underscores the potential of using self-adjuvanted protein nanoparticles for mucosal and systemic immune enhancement, providing a promising alternative to traditional vaccine formulations.

In aquaculture, the use of bacterial inclusion bodies (IBs) as immunostimulants has garnered attention for its potential to improve the immune function of farmed fish. These nanoparticles, which are formed during the recombinant protein production process, are highly stable and can be produced cost-effectively using bacterial systems. The study by Torrealba *et al.* (2024) explored the use of immune-related proteins produced as IBs, such as antimicrobial peptides and cytokines, and assessed their ability to stimulate immune responses in rainbow trout cells.^56^ The findings indicated that the inclusion bodies were able to enhance the expression of various immune-related genes, including those for pro-inflammatory cytokines such as TNFα and IL1β, which are crucial for mounting an effective immune response. Moreover, scaling up the production of IBs in bioreactors resulted in nanoparticles with improved immunomodulatory properties compared to those produced in smaller-scale systems. This research highlights the potential of using inclusion bodies as a tool for enhancing immune responses in aquaculture, providing a cost-effective and scalable method for improving disease resistance in farmed fish.

These studies collectively demonstrate the diverse ways in which nanoparticles can modulate the immune system. From enhancing phagocytic activity and promoting pro-inflammatory cytokine production to improving antigen delivery and supporting long-term immunity, these nanomaterials offer significant potential in combating infections. Whether through direct immune activation or as carriers for vaccines and therapeutic agents, their ability to elicit robust innate and adaptive immune responses positions them as valuable tools in the fight against infections, particularly in the face of increasing antimicrobial resistance.

##### 2.1.1.4 Biofilm disruption

Biofilms represent a major obstacle in the effective treatment of chronic and antibiotic-resistant infections due to their complex structure and inherent defence mechanisms. These microbial communities are encased in a self-produced extracellular polymeric substance (EPS) matrix, which serves as a physical barrier, limiting antibiotic penetration and protecting the bacteria within from host immune responses. Furthermore, biofilms promote a state of metabolic dormancy among embedded bacteria, reducing their susceptibility to antibiotics that target actively dividing cells. These characteristics make biofilm-associated infections notoriously difficult to treat and are a leading cause of persistent and recurrent infections.^57–59^

Polymetallic-based nanoparticles (PBNPs) have emerged as a transformative approach to biofilm disruption and eradication, employing a variety of mechanisms to overcome these challenges. Enzyme-loaded PBNPs have demonstrated exceptional efficacy in degrading the biofilm matrix. By incorporating enzymes such as DNase, which hydrolyzes extracellular DNA, or proteases, which break down protein components of the matrix, these nanoparticles weaken the structural integrity of biofilms. This enzymatic degradation not only disrupts the protective matrix but also facilitates the penetration of antibiotics and immune cells into the biofilm, enhancing pathogen clearance. The combination of matrix degradation and enhanced drug delivery makes enzyme-loaded PBNPs a promising strategy for targeting biofilm-associated infections.^60,61^

In addition to enzyme-based approaches, PBNPs functionalized with antimicrobial agents such as silver ions or other metal-based compounds exhibit synergistic effects in combating biofilms. Silver ions, for instance, are well-known for their broad-spectrum antimicrobial activity, which includes the ability to disrupt bacterial cell membranes, inhibit enzyme function, and induce oxidative stress. When incorporated into PBNPs, silver ions enhance the ability to not only target bacteria within biofilms but also destabilize the biofilm matrix itself. This dual mechanism of action is particularly effective against resilient biofilm-forming pathogens.^62–64^

Beyond direct antimicrobial activity, the functional versatility of PBNPs can be harnessed to deliver combination therapies targeting multiple aspects of biofilm resilience. For instance, PBNPs can be engineered to co-deliver antibiotics along with biofilm-disrupting agents, such as quorum-sensing inhibitors, to prevent bacterial communication and coordination essential for biofilm formation and maintenance. This multifaceted approach ensures that not only is the biofilm matrix dismantled, but the bacteria are also rendered more susceptible to treatment.

For example, Rajchakit *et al.* (2024) new antimicrobials are urgently needed to combat the rising global health concern of antibiotic resistance.^65^ Antimicrobial peptides (AMPs) are one of the leading candidates as new antimicrobials since they target bacterial membranes and are therefore less prone to bacterial resistance. However, poor enzymatic stability, high production costs, and toxicity are drawbacks that limit their clinical use. Conjugation of AMPs to gold nanoparticles (NPs) may help to improve enzymatic stability and, thus, their overall antimicrobial efficiency. We did a one-pot synthesis of size-controlled (10 nm) gold NPs selectively conjugated to lipopeptides and determined their antibacterial activity. The conjugates exhibited potent (0.13–1.25 μM) antimicrobial activity against clinical isolates, including Gram-positive methicillin-resistant *Staphylococcus aureus* (*S. aureus*) ATCC33593, Gram-negative *Escherichia coli* (*E. coli*) CTX-M-14, multidrug-resistant *Pseudomonas aeruginosa* LESB58 and Acinetobacter baumannii ATCC19606, and showed promising activity (90% inhibition of initial biofilms and 80% reduction of preformed biofilms) against *S. aureus* and *E. coli* DH5α biofilms at low micromolar concentrations. The conjugates were stable in rat serum and not toxic to representative mammalian cell lines *in vitro* (≤64 μM) and *in vivo* (≤100 μM).

Through these mechanisms, PBNPs not only enhance the efficacy of existing antimicrobial agents but also provide innovative solutions for overcoming microbial resistance and biofilm-associated challenges, positioning them as a transformative tool in modern antimicrobial therapy.

## 3 Key applications

### 3.1 Albumin based nanoparticles

Albumin-based nanoparticles (ABNPs) or albumin-incorporated nanoparticles have gained significant attention as effective antimicrobial agents due to their biocompatibility, non-toxicity, and versatile functionalization potential. Albumin serves as an excellent carrier for delivering antimicrobial agents, offering stability, controlled release, and enhanced bioavailability of the active compounds. These nanoparticles are typically synthesized using human serum albumin (HSA) or bovine serum albumin (BSA) and can encapsulate various antimicrobial agents, including antibiotics, antimicrobial peptides (AMPs), and metal nanoparticles like silver or zinc oxide.^66,67^

The antimicrobial efficacy of ABNPs lies in their ability to disrupt bacterial structures or inhibit essential metabolic pathways. When functionalized with AMPs or metallic nanoparticles, albumin provides a stable matrix that potentiates the activity of these agents. For example, silver nanoparticles embedded within an albumin matrix exhibit strong antibacterial activity by generating reactive oxygen species (ROS) and disrupting bacterial cell membranes. Similarly, the incorporation of AMPs into albumin nanoparticles enhances their stability and membrane-disruptive properties, enabling targeted action against Gram-positive and Gram-negative bacteria, including multidrug-resistant strains.^65^

In addition to their direct antimicrobial properties, albumin-based nanoparticles have been shown to mitigate the toxicity of potent antimicrobial agents. Encapsulation within an albumin matrix reduces systemic side effects while maintaining or enhancing the therapeutic effect of the antimicrobial agent. Furthermore, ABNPs facilitate targeted delivery to infection sites, improving the therapeutic index. This targeted approach also prevents the premature degradation of antimicrobial compounds, prolonging their efficacy.^68,69^

Albumin's inherent ability to bind various bioactive molecules and its biodegradability make it an ideal candidate for developing multifunctional antimicrobial platforms. Functionalizing ABNPs with surface ligands enables selective targeting of bacterial cells or biofilms, further enhancing their antimicrobial potency. Additionally, albumin's natural affinity for tumor microenvironments has inspired investigations into its use for simultaneous antimicrobial and anticancer therapies.^70,71^

Albumin-based nanoparticles and albumin-incorporated nanoparticles represent a versatile and effective strategy for combating microbial infections. Their ability to enhance the stability, bioavailability, and targeted delivery of antimicrobial agents, combined with their inherent biocompatibility and reduced toxicity, underscores their potential as a next-generation antimicrobial platform. Continued research into optimizing their synthesis, functionalization, and delivery mechanisms is likely to expand their applications in treating resistant infections and improving global health outcomes.

**Fig. 4:**
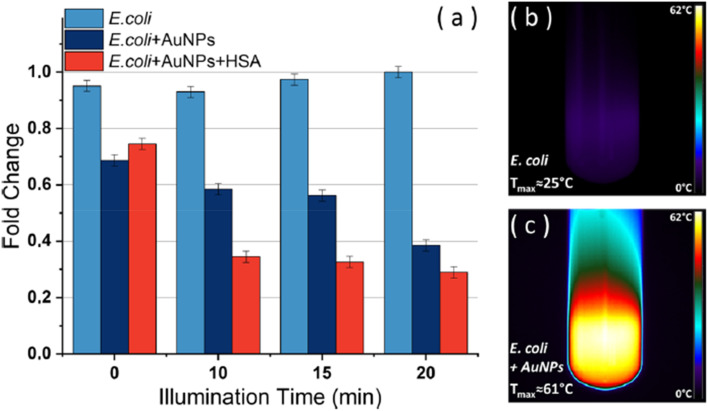
(a) Results of the viability experiments carried out with and without AuNPs and AuNPs + HSA at three different illumination times, along with representative infrared thermographic images of (b) *E. coli* and (c) *E. coli* + NPs samples after 20 min of laser irradiation. The values represent the mean of three independent triplicate experiments with standard error.^72^

For example, in the study by Guglielmelli *et al.* (2023), the antibacterial efficacy of PtNPs, AgNPs, and AuNPs, with and without HSA corona, was systematically evaluated against *Escherichia coli* (*E. coli*).^72^ Results indicated that the antibacterial effects were concentration-dependent and material-specific (Fig. 4). AgNPs demonstrated superior inhibition, achieving a 56% reduction in bacterial growth at 1.95 nM, while PtNPs induced a 62% reduction at 2.6 nM. AuNPs exhibited notable biocompatibility at lower concentrations, only achieving a 50% inhibition at the highest tested concentration of 6.4 nM. These observations align with previous reports highlighting AgNPs' pronounced antibacterial potency due to their high reactivity and ion release.

The addition of an HSA protein corona markedly altered the antibacterial outcomes. Across all nanoparticle types, the protein coating significantly reduced bacterial growth inhibition, with the effect not exceeding 20%, irrespective of the NP type or concentration. This protective effect likely stems from the biocompatible capping provided by HSA, which mitigates cytotoxicity through mechanisms such as decreased reactive oxygen species generation and reduced nanoparticle agglomeration. Fig. 4 illustrates the interaction between HSA-coated AuNPs and *E. coli*, highlighting the shielding effect of the protein corona on bacterial cells.

To further explore the potential of AuNPs, their plasmonic photothermal properties were investigated. When irradiated with a 532 nm laser, AuNPs exhibited efficient photothermal conversion, reaching temperatures above 60 °C, sufficient to induce bacterial cell death. Notably, when *E. coli* incubated with AuNPs was exposed to laser irradiation, significant antibacterial effects were observed, with bacterial growth reduction correlating with increased temperature and illumination time. Thermographic imaging, such as that depicted in Fig. 4c, confirmed the localized heating effect, demonstrating the synergistic potential of AuNPs and photothermal therapy.

**Fig. 5:**
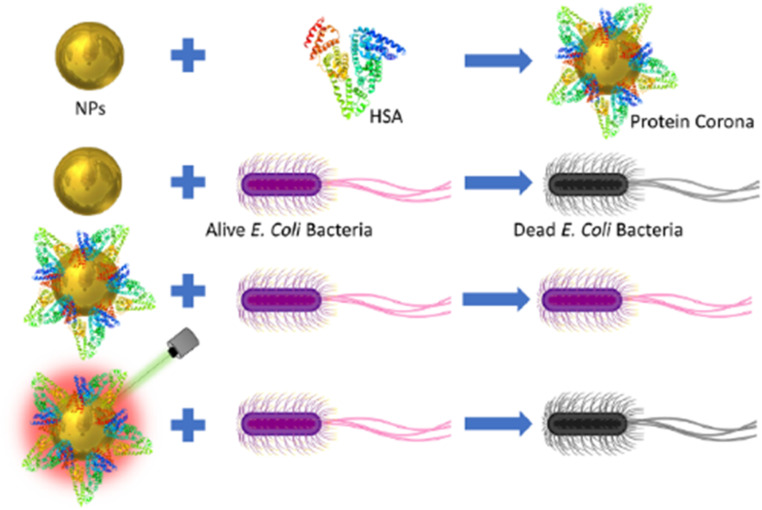
Schematic representation of the interaction between HSA and AuNPs, and the effect on *E. coli* bacteria with and without 532 nm laser irradiation.^72^

Scanning electron microscopy provided additional insights, revealing the localization of AuNPs near bacterial membranes, both with and without the HSA corona. These visual findings reinforced the conclusion that while HSA reduces the direct antibacterial efficacy of NPs, its presence does not inhibit the photothermal activity of AuNPs under laser irradiation. The schematic in Fig. 5 effectively encapsulates these dynamics, illustrating the contrasting effects of HSA-coated and uncoated AuNPs on *E. coli* with and without laser exposure.

Overall, this study underscores the nuanced role of the protein corona in dictating the antibacterial performance of noble metal nanoparticles. While HSA diminishes direct antibacterial effects, the photothermal properties of AuNPs remain unaffected, presenting a promising avenue for laser-assisted antibacterial therapies. These findings contribute valuable insights into the design of nanoparticle-based strategies to combat antibiotic resistance.

The development of innovative strategies to combat microbial infections has become crucial in light of the growing threat of bacterial resistance to conventional antibiotics. The research by Wang *et al.* (2022) exemplifies the potential of alternative antimicrobial approaches, focusing on the design and synthesis of antimicrobial peptides (AMPs). AMPs, which are known for their unique mechanisms of action, offer a promising solution as they are less likely to induce bacterial resistance compared to traditional antibiotics.^73^ Wang *et al.* introduced LP21, a lipopeptide that demonstrated potent antimicrobial activity, serum stability, low cytotoxicity, and high membrane-disruptive ability. These properties make LP21 an attractive candidate for antimicrobial treatment. Further enhancing the potential of this peptide, LP21 was able to self-assemble into spherical aggregates in aqueous solutions, encapsulating tetracycline (TC) to form the LP21@TC nanomedicine. This nanomedicine not only improved the bioavailability and stability of the antimicrobial agent but also exhibited synergistic effects, amplifying its therapeutic outcomes *in vivo*. This highlights the significance of incorporating advanced nanomedicine strategies, such as albumin-based nanoparticles, to enhance the stability and bioavailability of antimicrobial agents and target bacterial infections more effectively.

Similarly, Tarhini *et al.* (2020) explored the encapsulation of therapeutic proteins within human serum albumin (HSA) nanoparticles, presenting another compelling approach to antimicrobial therapy.^74^ HSA nanoparticles were successfully prepared using a nanoprecipitation method, resulting in particles with a size of 120 nm and a negative zeta potential of −25 mV. These nanoparticles encapsulated two distinct proteins, neutrophil elastase (NE) and secretory leukocyte protease inhibitor (SLPI), each of which displayed different behaviors upon encapsulation. While encapsulated NE lost its proteolytic activity, SLPI retained its inhibitory properties. This finding underscores the potential of albumin nanoparticles as carriers for both enzymes and antimicrobial agents, offering a platform for controlled drug delivery. In antibacterial studies, both NE- and SLPI-loaded HSA nanoparticles were able to significantly reduce bacterial growth of *Pseudomonas aeruginosa*, demonstrating the therapeutic value of protein encapsulation. The ability of albumin-based nanoparticles to enhance the stability and efficacy of encapsulated agents aligns with the growing body of research supporting their use in antimicrobial therapies. By incorporating antimicrobial peptides or proteins into albumin nanoparticles, it is possible to improve the delivery and targeted action of these agents, thus overcoming some of the limitations posed by traditional antibiotics.

Together, these studies contribute to the emerging field of albumin-based nanoparticle platforms for antimicrobial therapies. The ability of these nanoparticles to encapsulate a variety of therapeutic agents, including antimicrobial peptides and proteins, and enhance their stability, bioavailability, and targeted delivery is a promising approach to addressing the challenge of antibiotic resistance. Further optimization of the synthesis, functionalization, and delivery mechanisms of albumin-based nanoparticles could expand their applications in combating resistant infections and improving global health outcomes. By continuing to explore their potential, it is likely that albumin-incorporated nanoparticles will play an increasingly pivotal role in the development of next-generation antimicrobial treatments.

### 3.2 Lactoferrin nanoparticles

Lactoferrin nanoparticles (LF-NPs) are an innovative platform in nanotechnology, leveraging the intrinsic bioactivity of lactoferrin to deliver potent antimicrobial and anti-inflammatory effects. Lactoferrin, a glycoprotein naturally found in milk and other bodily fluids, possesses the remarkable ability to inhibit the growth of a broad spectrum of pathogens, including bacteria and fungi. By integrating lactoferrin into nanoparticulate systems, its therapeutic potential is significantly enhanced, allowing for targeted delivery and improved stability in biological environments. This approach has shown promise in addressing infections and inflammatory disorders, particularly where conventional treatments face limitations such as resistance or systemic toxicity.^75–77^

The antimicrobial properties of lactoferrin are attributed to its capacity to chelate iron, an essential nutrient for microbial growth, effectively starving pathogens of this critical resource. Additionally, lactoferrin directly disrupts microbial membranes, causing structural damage that leads to cell death. When formulated as nanoparticles, lactoferrin's surface charge and binding affinity are optimized to enhance its interaction with microbial cells. This design ensures superior efficacy against both Gram-positive and Gram-negative bacteria, as well as various fungal strains. LF-NPs also demonstrate unique biofilm-disrupting capabilities, addressing one of the major challenges in treating persistent infections.^75,76^

Equally important is lactoferrin's role in modulating inflammation, making LF-NPs a dual-action therapeutic tool. By inhibiting key pro-inflammatory pathways such as the NF-κB signalling cascade, lactoferrin reduces the production of cytokines like TNF-α, IL-1β, and IL-6, which are central mediators of inflammation. This anti-inflammatory action not only helps control infections but also protects tissues from collateral damage caused by an overactive immune response. The nanoparticulate form enhances these effects by ensuring that lactoferrin is delivered directly to inflamed or infected sites, where its therapeutic action is most needed.^78,79^

The broad-spectrum efficacy of LF-NPs is further augmented by their biocompatibility and safety profile, making them suitable for diverse applications. Studies have demonstrated that LF-NPs are non-toxic to mammalian cells, even at concentrations required for antimicrobial activity. This characteristic is particularly beneficial in treating infections in sensitive tissues, such as the gastrointestinal tract, lungs, and skin, where traditional antimicrobial agents may cause irritation or damage.^80,81^ Moreover, LF-NPs offer controlled and sustained release of their therapeutic payload, ensuring prolonged activity and reducing the frequency of administration. Emerging research underscores the versatility of LF-NPs in combating drug-resistant pathogens, which pose a growing threat to global health. By circumventing traditional resistance mechanisms, such as efflux pumps and enzymatic degradation, lactoferrin nanoparticles provide a robust alternative to conventional antibiotics and antifungal agents. Additionally, their synergistic potential with existing therapies offers a promising avenue for combination treatments, enhancing the overall effectiveness of antimicrobial regimens.^82,83^

In a nutshell, lactoferrin nanoparticles exemplify the convergence of nanotechnology and biology, harnessing the natural properties of lactoferrin to address pressing challenges in infectious disease management and inflammatory disorders. Their ability to target pathogens while simultaneously modulating the immune response positions them as a transformative solution in modern medicine. With ongoing advancements in nanoparticle engineering and a deeper understanding of lactoferrin's mechanisms, LF-NPs are poised to play a critical role in the next generation of therapeutic innovations.

For example, lactoferrin nanoparticles (LF-NPs) have garnered significant attention due to their multifunctional properties, including antimicrobial and anti-inflammatory effects. Luo *et al.* (2021) explored these properties comprehensively, focusing on their capacity to address inflammation and bacterial infections, particularly in the gastrointestinal system.^84^ Their study elucidates the unique targeting capabilities of LF-NPs, attributed to the inherent ligand properties of lactoferrin (LF) and its interaction with specific receptors in inflamed tissues.

**Scheme 1:**
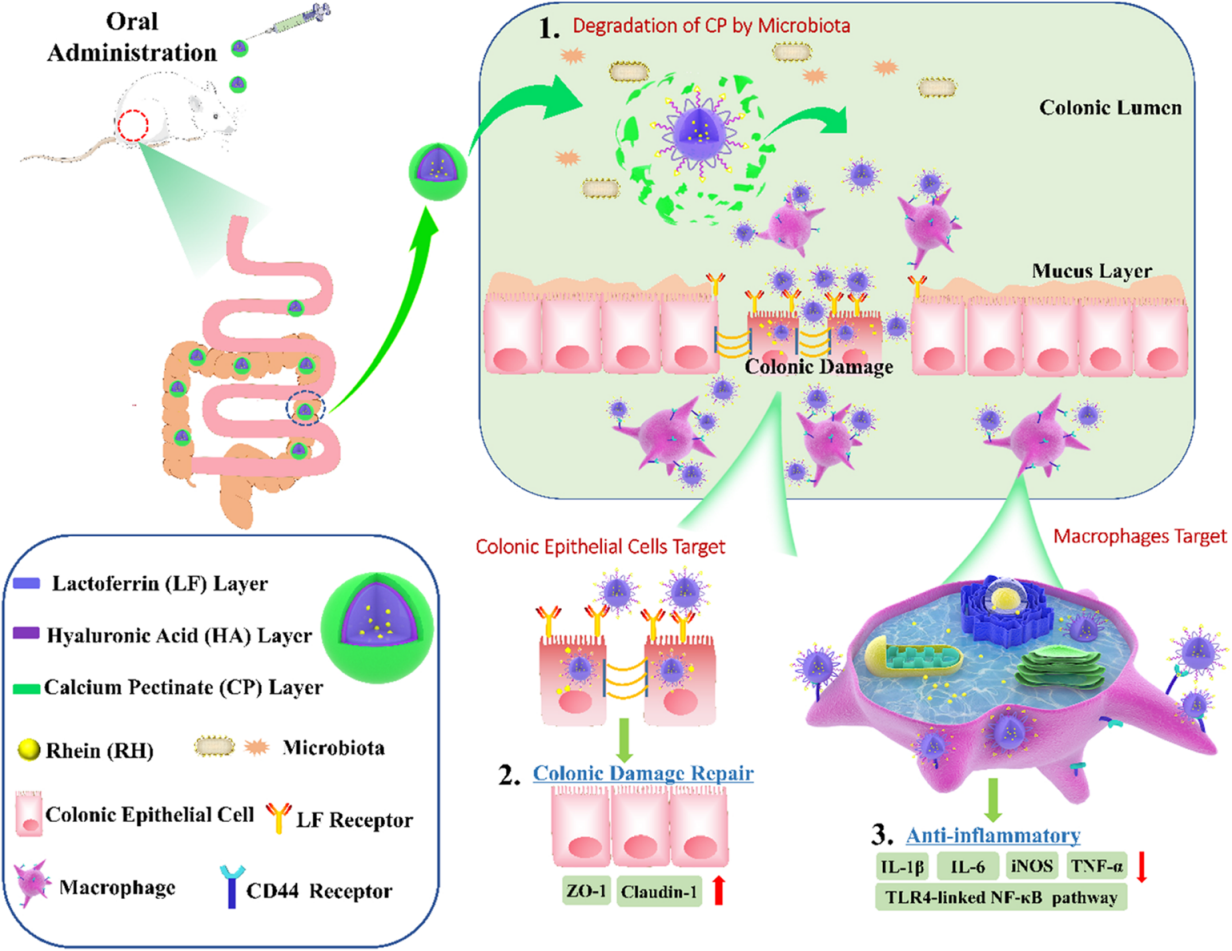
Schematic illustration of using CP/HA/RH-NPs in the treatment of UC in mice. (1) Schematic illustration of the passage of orally administered CP/HA/RH-NPs through GIT. CP could protect CP/HA/RH-NPs to pass through stomach and small intestine, and further release HA/RH-NPs into colonic lumen due to its degradation. (2) Schematic illustration of enhancing the effects of RH in repairing intestinal damage by adjusting ZO-1 and Claudin-1 expression in the UC mice model by colonic epithelial cell target. (3) Schematic illustration of targeting macrophage could effectively promote RH's anti-inflammatory effect through the TLR4/MyD88/NF-κB pathway in *in vivo* anti-UC therapeutic efficacy.^84^

In their experimental framework, Luo *et al.* synthesized core–shell structured nanoparticles incorporating IR780 and RH drugs, with lactoferrin as the primary ligand.^84^Scheme 1 illustrates the design and functional assembly of these nanoparticles. The study demonstrated that LF-NPs outperformed conventional drug delivery systems in selectively targeting inflamed areas in colitis models. This selectivity is achieved through the synergistic actions of lactoferrin's antimicrobial peptides and its anti-inflammatory mechanisms, which inhibit key inflammatory pathways, including the TLR4/NF-κB signaling cascade. Luo *et al.* further highlighted the dual advantage of LF-NPs: their ability to actively target pathogens while reducing inflammation through modulation of cytokine production. The fluorescence imaging results (Scheme 1) showed significant accumulation of LF-NPs in inflamed colon tissues, underlining their enhanced site-specific delivery compared to free drugs or untargeted nanoparticles. These findings substantiate the potential of LF-NPs in mitigating symptoms of diseases like ulcerative colitis by addressing both microbial infection and the associated inflammatory response.

Moreover, the study delves into the structural features of LF-NPs that contribute to their efficacy. The protective coating of chitosan prevents premature drug release, ensuring that lactoferrin's antimicrobial and anti-inflammatory functions remain intact until the nanoparticles reach the target site. Scheme 1 effectively captures the role of this chitosan shell in maintaining the functional integrity of the nanoparticles, which correlates directly with their prolonged therapeutic effect.

In DSS-induced colitis models, the study observed marked improvements in clinical parameters, including bodyweight recovery, reduced disease activity index (DAI) scores, and normalized colon length, upon treatment with LF-NPs. These therapeutic outcomes are a direct result of lactoferrin's innate ability to bind to bacterial lipopolysaccharides (LPS), neutralize reactive oxygen species (ROS), and suppress pro-inflammatory cytokines like TNF-α, IL-6, and IL-1β. Histopathological analyses also revealed that LF-NPs preserved intestinal tight junction proteins, such as ZO-1 and Claudin-1, which are crucial for maintaining the epithelial barrier's integrity. This protective effect highlights the nanoparticles' role in ameliorating the damage associated with intestinal inflammation, as depicted in Scheme 1.

In a nutshell, Luo *et al.* (2021) provide compelling evidence for the versatility of LF-NPs in therapeutic applications, particularly in combating microbial infections and reducing inflammation.^84^ Their innovative design, as illustrated in Scheme 1, offers a platform for further exploration of lactoferrin's therapeutic potential, paving the way for advanced treatments in gastrointestinal and other inflammation-mediated diseases.

The study by Abdel-Wahab *et al.* (2021) highlights the significant impact of lactoferrin in enhancing immunity and antioxidative capacity in aquaculture species when integrated into a dietary regime.^85^ This investigation examined the effects of bovine lactoferrin (BLF), chitosan nanoparticles (CHN), and their combinations on the health and disease resistance of Nile tilapia (*Oreochromis niloticus*) when challenged with *Aeromonas hydrophila*. The inclusion of BLF and CHN in the fish diet was associated with improvements in serum biochemical indices, enzymatic activities, transcriptomic responses, and non-specific immunity. Notably, the combined treatments showed the most pronounced effects, including elevated activities of antioxidative enzymes such as superoxide dismutase (SOD), catalase (CAT), and glutathione peroxidase (GSH-Px). The ability of lactoferrin, even when combined with other nanomaterials like CHN, to enhance immune functions and antioxidative responses aligns with the broad-spectrum activity and dual-action properties described for LF-NPs. Furthermore, the upregulation of immune-related genes, including IL-1β and IFN-γ, and the observed resistance against pathogenic challenges reflect lactoferrin's role as a critical immunomodulatory agent. These findings underscore the utility of lactoferrin-based formulations, particularly as nanoparticles, to bolster health and resilience in aquaculture species, showcasing a clear parallel with its application in advanced therapeutic contexts.

Similarly, López-Machado *et al.* (2021) explored the potential of lactoferrin-loaded nanoparticles for the treatment of ocular inflammation, a condition often exacerbated by the challenges of conventional treatments, including poor bioavailability and significant side effects.^86^ This study successfully encapsulated lactoferrin within biodegradable polymeric nanoparticles, achieving a monodisperse population with an average size of approximately 130 nm and a positive surface charge. The nanotechnological approach addressed the limitations associated with lactoferrin's aqueous instability and rapid clearance, resulting in a sustained release profile that enhanced its pharmacokinetics and pharmacodynamics. Importantly, these nanoparticles were shown to be non-cytotoxic and non-irritant in both *in vitro* and *in vivo* assays, reinforcing their safety profile. The anti-inflammatory efficacy of lactoferrin nanoparticles was significantly improved in cell culture and preclinical models, illustrating their potential to mitigate inflammation in ophthalmic conditions. This study exemplifies how nanotechnology amplifies the innate properties of lactoferrin, leveraging its natural anti-inflammatory and antimicrobial activities for targeted therapeutic applications. The findings mirror the broader utility of LF-NPs as a transformative tool in biomedical interventions, particularly where traditional formulations fall short.

In both studies, the incorporation of lactoferrin into nanoparticulate systems reveals its ability to extend beyond its natural bioactivities, enabling more effective, stable, and targeted applications. Abdel-Wahab *et al.* (2021)^85^ and López-Machado *et al.* (2021)^86^ collectively emphasize the potential of lactoferrin nanoparticles to address pressing challenges in both veterinary and human medicine.^86^ These findings further support the concept that LF-NPs represent a versatile and promising solution for enhancing health outcomes across diverse fields, from aquaculture to ophthalmology.

### 3.3 Gelatin nanoparticles

Gelatin nanoparticles have emerged as a versatile and effective platform for delivering antimicrobial agents, particularly silver ions and antimicrobial peptides, due to their biocompatibility, biodegradability, and ability to encapsulate bioactive compounds. Gelatin, a natural polymer derived from collagen, is widely used in biomedical applications because of its excellent compatibility with human tissues and its capacity to be easily processed into nanoparticles.^87,88^ When gelatin nanoparticles are loaded with silver ions or antimicrobial peptides, they acquire enhanced antimicrobial properties that make them particularly effective against biofilm-forming bacteria, which are often resistant to conventional antibiotics.^89^ Biofilms, which are clusters of bacteria encased in a protective extracellular matrix, are notoriously difficult to treat because they provide bacteria with a shield against immune responses and antibiotics. This makes infections involving biofilms chronic and resistant to treatment, posing significant challenges in clinical settings, particularly in wound healing, chronic infections, and medical device-related infections.^41,89^

The loading of silver ions into gelatin nanoparticles is particularly effective due to the intrinsic antimicrobial properties of silver. Silver ions are known to disrupt bacterial cell walls, interfere with DNA replication, and generate reactive oxygen species (ROS), which collectively contribute to the destruction of bacterial cells.^90^ When encapsulated in gelatin nanoparticles, silver ions are released in a controlled manner, allowing for sustained antimicrobial activity. This controlled release not only improves the efficacy of silver ions by maintaining therapeutic concentrations at the infection site for extended periods but also minimizes the risk of toxicity to surrounding healthy tissues.^91^ The combination of silver ions with gelatin nanoparticles creates a synergistic effect, enhancing the material's ability to combat both planktonic (free-floating) and biofilm-associated bacteria. Silver-loaded gelatin nanoparticles have shown promising results in the treatment of a variety of bacterial infections, including those caused by multi-drug-resistant organisms like methicillin-resistant *Staphylococcus aureus* (MRSA) and *Pseudomonas aeruginosa*, which are often associated with chronic wound infections and medical device-related infections.^90,91^

In addition to silver ions, antimicrobial peptides (AMPs) are another class of bioactive compounds that can be effectively incorporated into gelatin nanoparticles to combat biofilm-forming bacteria. AMPs are naturally occurring peptides that possess broad-spectrum antimicrobial activity against a wide range of pathogens, including bacteria, fungi, and viruses. They exert their antimicrobial effects by interacting with the bacterial cell membrane, disrupting its integrity, and inducing cellular damage.^92^ When loaded into gelatin nanoparticles, AMPs can be delivered to the infection site in a controlled and sustained manner, improving their effectiveness while reducing potential side effects. The encapsulation of AMPs in nanoparticles also protects them from degradation by proteases and ensures their stability in biological environments.^93^ Furthermore, because AMPs have a multifaceted mechanism of action, they are particularly useful in treating biofilm-associated infections, as they can penetrate the extracellular matrix of biofilms and directly target bacterial cells. This makes AMPs an ideal therapeutic agent for chronic infections, where biofilm formation is a major barrier to treatment.^94^

The application of gelatin nanoparticles loaded with silver ions or antimicrobial peptides extends beyond bacterial infections to include various medical and therapeutic contexts. For instance, in wound healing, the use of these nanoparticles can significantly improve the healing process by preventing infection, reducing inflammation, and promoting tissue regeneration. The nanoparticles' ability to deliver antimicrobial agents locally at the site of infection helps to maintain a sterile environment, reducing the risk of infection and promoting faster recovery. Furthermore, the biocompatibility and biodegradability of gelatin ensure that these nanoparticles do not accumulate in the body or cause adverse reactions, making them a safe option for long-term use in medical treatments.^90,95^

The combination of gelatin's natural properties with the antimicrobial efficacy of silver ions or AMPs in nanoparticle form provides a powerful strategy for combating biofilm-forming bacteria. The versatility and effectiveness of these nanoparticles in treating chronic infections, including those associated with wounds, implants, and medical devices, offer promising prospects for future therapeutic applications. As research progresses, the ability to fine-tune the size, surface properties, and drug-release profiles of these nanoparticles will further enhance their effectiveness, providing more targeted and efficient treatments for biofilm-related infections. Thus, gelatin nanoparticles loaded with silver ions or antimicrobial peptides represent a promising platform for developing novel antimicrobial therapies with the potential to address the growing problem of antibiotic resistance and biofilm-associated infections in clinical medicine.^89–98^

The study by Patarroyo *et al.* (2022) addresses the growing concern of antibiotic resistance, a critical public health issue resulting from the excessive and improper use of antibiotics.^99^ This resistance has made treating microbial infections, which were once manageable, increasingly challenging. To combat this, the study explored the development of innovative treatments combining antimicrobial peptides (AMPs) with nanomaterials to create a novel hydrogel-based therapy. This therapy consists of a polymeric network of gelatin, polyvinyl alcohol (PVA), and hyaluronic acid, encapsulating graphene oxide (GO) nanoconjugates loaded with silver nanoparticles (AgNPs). These hydrogels are designed to be stable, biocompatible, non-toxic, and capable of maintaining prolonged bioavailability of the active agents at the site of application, effectively inhibiting microbial growth.

The nanocomposite hydrogels were thoroughly characterized for their microstructure, thermal resistance, rheological behaviour, particle size distribution, texture profile, and stability. The satisfactory results from these evaluations allowed the researchers to fine-tune their formulation to encapsulate the GO–AgNP nanoconjugates effectively. Biological evaluations demonstrated the hydrogels' biocompatibility, with low hemolytic effects (less than 5%) and moderate platelet aggregation capacity (35–45%). The hydrogels showed a remarkable ability to inhibit bacterial growth entirely.

**Fig. 6:**
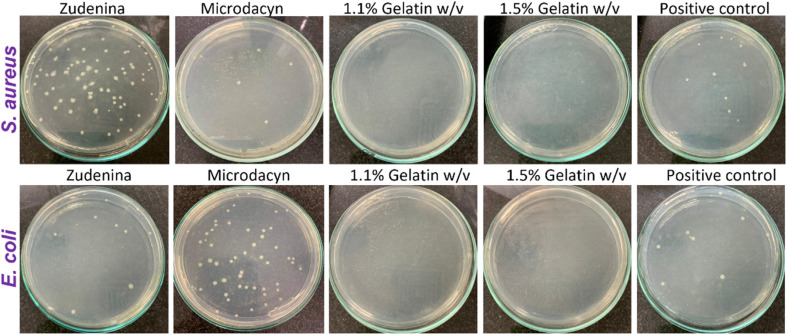
Representative photos of bacteria colonies counting on Mueller–Hinton agar plates after 18 h incubation at 37 °C and exposed to our topical formulations with 1.1% and 1.5% (w/v) gelatin with 0.7% (w/v) PVA and 20 μg per mL GO–Ag NPs, the commercial treatments, and the positive control (*n* = 15).^99^

As depicted in Fig. 6, the bacterial colony count analysis on Mueller–Hinton agar plates revealed that topical nanocomposite hydrogel treatments containing 1.1% and 1.5% (w/v) gelatin achieved a 100% reduction in bacterial growth, even at a low nanoconjugate dose of 20 μg mL^−1^. This antimicrobial efficacy significantly outperformed commercial treatments such as Zudenina® and Microdacyn®, which showed limited effectiveness. Zudenina® reduced bacterial growth by approximately 50% for *S. aureus* and *E. coli*, while Microdacyn® eliminated only about 30% of *S. aureus* and failed to eliminate *E. coli*. These results underscore the superior antimicrobial performance of the gelatin-based nanocomposite hydrogels, highlighting their potential for addressing biofilm-forming bacterial infections.

**Fig. 7:**
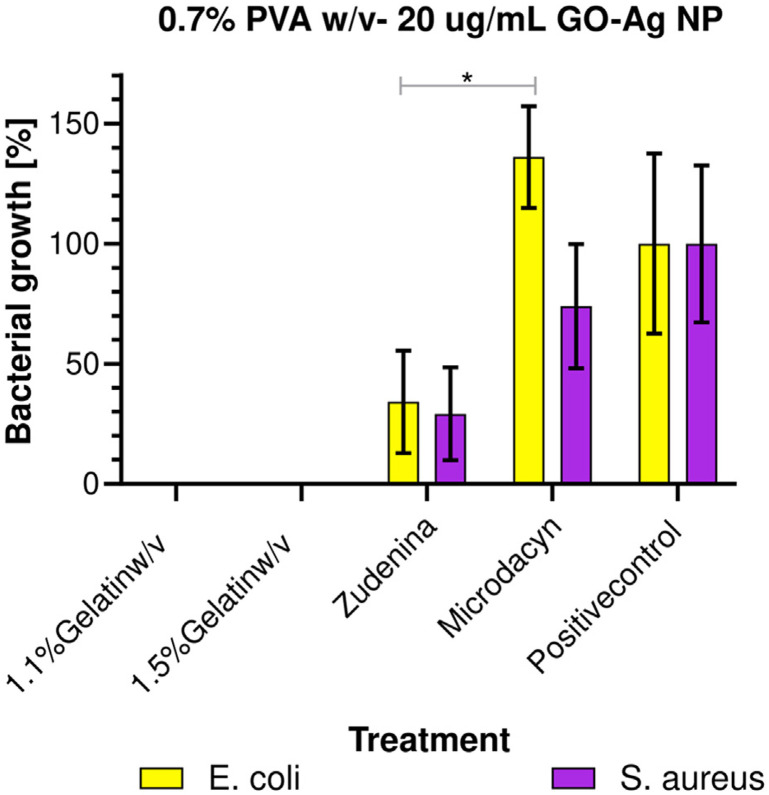
Bacterial growth for the 1.1% and 1.5% (w/v) gelatin with 0.7% (w/v) PVA and 20 μg per mL GO–Ag NPs treatment, the commercial treatments, and the positive control (*n* = 15). *, *p* < 0.05.^99^

The mechanism of action likely involves the ability of AgNPs to disrupt bacterial cell walls and penetrate cells, leading to irreversible damage to intracellular machinery and subsequent cell death. The findings, as illustrated in Fig. 7, demonstrate the complete bacterial elimination by the developed hydrogels, making them highly effective against pathogens like *E. coli* and *S. aureus*, which are common culprits of healthcare-associated infections (HAIs).

Moreover, the cytotoxicity of the hydrogels was assessed *via* an MTT assay. The results revealed high biocompatibility, with cell viability exceeding 80% at extract concentrations below 12.5% (v/v). At higher concentrations, cytotoxicity increased in a concentration-dependent manner. The 1.5% (w/v) gelatin hydrogel exhibited lower toxicity compared to the 1.1% (w/v) formulation, which could be attributed to the more efficient encapsulation and controlled release of the GO–AgNP nanoconjugates in the higher gelatin concentration. This observation highlights the importance of optimizing gelatin concentrations to enhance encapsulation efficiency and minimize potential cytotoxic effects.

In summary, the study demonstrates the exceptional potential of gelatin-based hydrogels loaded with silver nanoparticles and antimicrobial peptides for combating biofilm-forming bacteria. Their high efficacy, stability, and biocompatibility position these hydrogels as promising candidates for advanced antimicrobial treatments, wound healing, bioadhesives, and tissue engineering applications. Further developments, such as incorporating anti-inflammatory or immunomodulatory agents, could expand their utility in addressing complex biomedical challenges.

The study by Zharkova *et al.* (2021) delves into the synergistic antibacterial activity of silver nanoparticles (AgNPs) conjugated with antimicrobial peptides (AMPs) or proteins (APs), focusing on their enhanced efficacy against biofilm-forming bacteria and drug-resistant pathogens.^100^ This work aligns closely with the theme of gelatin nanoparticles as effective vehicles for antimicrobial agents, showcasing how gelatin-based formulations can be tailored to amplify the therapeutic potential of silver ions and peptides.

The research highlights the conjugation of AgNPs with protegrin-1 (PG-1), indolicidin, protamine, histones, and lysozyme to explore their antibacterial properties. The conjugates demonstrated superior efficacy compared to their individual components, with PG-1 emerging as the most potent due to its small and rigid membranolytic structure. Notably, PG-1 retains its activity when conjugated, as evidenced by the characteristic sharp rise and plateau in the permeabilization curve, indicative of its specific interaction with bacterial membranes. Conversely, histones and protamine exhibit reduced permeabilizing capacity in the conjugated form but still outperform non-conjugated AgNPs.

The study also examines the impact of these conjugates on the cytoplasmic membrane, finding that the effects are significantly diminished compared to free peptides. This selective permeabilization suggests that the conjugation strategy not only enhances the antimicrobial action but also mitigates the toxicity associated with free peptides.

**Fig. 8:**
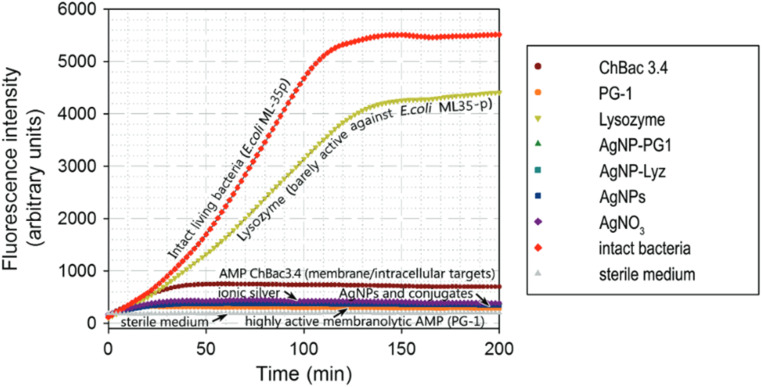
Inhibition dynamics of metabolic activity in *E. coli* ML-35p by AgNP–AMP/AP conjugates. The metabolic inhibitory effects of AgNP–AMP/AP conjugates are compared with those of free antimicrobial peptides and proteins (AMPs/APs), gelatin-only coated silver nanoparticles (AgNPs), and ionic silver (AgNO_3_). Actively metabolizing bacterial cells reduce the redox-sensitive marker resazurin to its fluorescent product, resorufin. An increase in fluorescence intensity reflects active bacterial metabolism, while a decline indicates metabolic inhibition. The antimicrobials were tested at a concentration of 4× the minimal inhibitory concentration (MIC). Representative response curves illustrate the rapid and potent metabolic suppression induced by the conjugates and their components.^100^

The ability of AgNP–AMP/AP conjugates to inhibit bacterial metabolism was assessed using a resazurin-based fluorometric assay. As shown in Fig. 8, the conjugates rapidly impeded bacterial respiratory activity, with resorufin fluorescence plateauing within 30 minutes. This rapid metabolic inhibition parallels the effects of free PG-1, gelatinized AgNPs, and ionic silver, suggesting a direct impact on the bacterial respiratory chain. While the exact mechanism remains uncertain, the lesser effect of the partially membranolytic ChBac3.4 and the absence of significant cytoplasmic membrane disruption point toward a targeted action on the respiratory machinery rather than collateral membrane damage.

The dual functionality of the AgNP–AMP/AP conjugates extends beyond antibacterial activity. The study notes that certain conjugates selectively target tumor cells while maintaining low hemolytic activity against normal eukaryotic cells. These findings underscore the potential of gelatin-based nanoparticle systems as versatile platforms for antimicrobial and anticancer therapies. Furthermore, the partial transfer of immunomodulatory and wound-healing properties from AMPs to conjugated nanoparticles suggests broader biomedical applications.

In summary, Zharkova *et al.* (2021) provide compelling evidence that functionalizing gelatin nanoparticles with silver ions and antimicrobial peptides significantly enhances their therapeutic efficacy.^100^Fig. 8 vividly illustrate the mechanisms underlying their antibacterial and metabolic inhibitory effects, affirming the utility of such conjugates in addressing biofilm-associated infections and drug-resistant pathogens while minimizing cytotoxicity.

Huang *et al.* (2022) developed a biodegradable cryogel incorporating gelatin and Ag NPs, which demonstrated exceptional properties for treating burn wounds.^101^ The cryogel's high water absorption capacity, swelling ratio of up to 4000%, and effective antibacterial activity, particularly against methicillin-resistant *Staphylococcus aureus* (MRSA) and *Pseudomonas aeruginosa* (PA), made it highly effective in treating burn wounds. The cryogel not only exhibited antibacterial properties but also showed excellent antibiofilm activity, a key feature in wound healing. Biofilm formation is a significant challenge in treating chronic wounds, as it provides a protective barrier for bacteria, making infections difficult to eradicate. The inclusion of Ag NPs in the cryogel was particularly beneficial in disrupting bacterial cell walls and inhibiting biofilm formation, aligning with the effectiveness of gelatin-based nanoparticles loaded with antimicrobial agents in preventing biofilm-related infections. Moreover, the cryogel's ability to absorb exudates from wounds while promoting gas exchange further enhances its suitability as a wound dressing. The biocompatibility and the effective degradation of the cryogel within four weeks also highlight its potential for long-term use in clinical applications.

In a similar vein, Alven *et al.* (2024) explored the potential of gelatin-based sponges loaded with silver nanoparticles and metronidazole for wound healing.^102^ These sponges exhibited a significant degree of cross-linking, which influenced their porosity and drug release properties. The sponges displayed an initial burst release of metronidazole followed by a sustained release, a critical feature for continuous antibacterial activity. The antibacterial efficacy of the sponges was evident against both Gram-positive and Gram-negative bacteria, further emphasizing the potential of gelatin-based materials combined with silver nanoparticles for treating infected wounds. The high degree of biocompatibility (cell viability ranging from 71.17–86.10%) and promising results from *in vitro* experiments, where the sponge loaded with metronidazole showed significant reduction in the wound area, suggest that these sponges are well-suited for clinical applications. The ability of these sponges to deliver antimicrobial agents in a controlled manner, alongside the presence of silver nanoparticles, positions them as an effective treatment for bacteria-infected wounds, particularly in addressing the issues of bacterial resistance and biofilm formation.

All these studies underline the growing interest in gelatin-based nanoparticles and sponges, particularly those loaded with silver nanoparticles or antimicrobial peptides, as a promising solution for treating bacteria-infected wounds. The ability of these materials to inhibit biofilm formation, release antimicrobial agents in a controlled manner, and promote wound healing through enhanced antibacterial and anti-inflammatory activity makes them highly effective in addressing the challenges posed by chronic and burn wounds. The promising results from *in vitro* experiments in these studies suggest that gelatin-based materials hold great promise for future clinical applications, offering a combination of antibacterial efficacy, biocompatibility, and effective wound healing.

### 3.4 Peptide-based nanoparticles as innovative antibacterial agents

Peptide-based nanoparticles (PBNPs) have emerged as a groundbreaking solution in the search for effective antibacterial agents, especially in light of the growing concern over antibiotic-resistant bacteria. Conventional antibiotics, while effective in many cases, are becoming less useful as bacteria evolve mechanisms to resist their effects. This has led to an urgent need for novel therapeutic strategies that can target bacterial infections in new and more efficient ways. PBNPs offer a promising alternative, combining the intrinsic antimicrobial properties of peptides with the enhanced stability and controlled release capabilities afforded by nanotechnology. These systems hold significant potential for overcoming many of the challenges associated with traditional antibiotics, including the development of resistance, toxicity, and poor pharmacokinetics.^103,104^

One of the key advantages of PBNPs is their ability to exploit the natural antimicrobial properties of peptides, which are short chains of amino acids that can disrupt bacterial membranes, inhibit cell wall synthesis, or interfere with essential cellular processes. Peptides can be designed with high specificity to target bacterial pathogens, and their activity is often more potent against drug-resistant strains compared to conventional antibiotics. By incorporating these peptides into nanoparticle formulations, the overall stability and bioavailability of the peptides can be significantly improved, making them more effective in treating infections. Furthermore, nanoparticles provide a means of protecting the peptides from degradation, ensuring they reach their target site in the body intact and with sustained release over time.^105,106^

The use of nanotechnology in PBNPs allows for the fine-tuning of their properties, including size, surface charge, and morphology, which can be optimized for specific applications. These characteristics are crucial for determining how well the nanoparticles interact with bacterial cells and tissues, as well as their ability to penetrate biological barriers. For example, nanoparticles can be engineered to enhance their uptake by bacterial cells, ensuring that the peptide payload is delivered efficiently to the site of infection. Additionally, the surface of the nanoparticles can be modified with targeting ligands that allow for selective binding to specific bacterial strains or tissues, further improving their therapeutic efficacy while minimizing side effects.^107,108^

Another significant benefit of PBNPs is their potential to mitigate the problem of antibiotic resistance. Traditional antibiotics often become less effective as bacteria develop resistance mechanisms, such as altering their cell wall structure or producing enzymes that degrade the drug. In contrast, the antimicrobial peptides used in PBNPs generally have multiple mechanisms of action, making it more difficult for bacteria to develop resistance. Furthermore, the nanoparticles can be engineered to release the peptides in a controlled manner, ensuring that the antimicrobial agents remain active at the site of infection for longer periods, reducing the chances of resistance development.^109,110^

In addition to their antibacterial properties, PBNPs have demonstrated low toxicity to human cells, making them highly biocompatible. This is an important consideration in the development of new antibacterial therapies, as many traditional antibiotics can cause adverse side effects, particularly with prolonged use. The biocompatibility of PBNPs makes them suitable for clinical applications, including the treatment of both localized and systemic infections. Moreover, the ability to modify the surface properties of the nanoparticles also allows for the incorporation of additional functionalities, such as anti-inflammatory or immunomodulatory effects, which could further enhance the therapeutic potential of these systems.^110,111^

The use of PBNPs is not limited to bacterial infections alone. Their versatility also extends to addressing fungal, viral, and even parasitic infections, expanding their potential therapeutic applications. Additionally, peptide-based nanoparticles can be used in combination with other treatment modalities, such as photothermal therapy or immunotherapy, to create multifunctional systems that are more effective against complex infections. As research into PBNPs continues to grow, it is likely that these systems will play a central role in the development of next-generation antibacterial therapies, providing a powerful tool to combat the global challenge of antibiotic resistance.^112,113^

In a nutshell, peptide-based nanoparticles represent a promising and innovative approach to antibacterial therapy. Their ability to combine the antimicrobial power of peptides with the advantages of nanotechnology, such as improved stability, targeted delivery, and controlled release, makes them an exciting alternative to traditional antibiotics. With ongoing advancements in nanoparticle design and peptide engineering, PBNPs have the potential to revolutionize the treatment of bacterial infections, offering new hope in the fight against antibiotic-resistant pathogens.

**Fig. 9:**
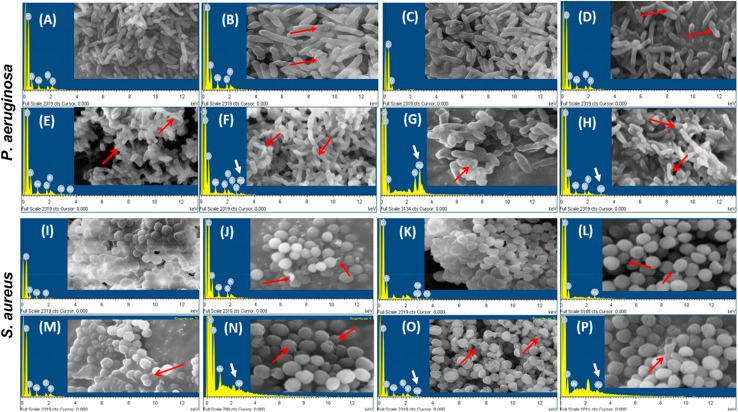
SEM analysis of bacterial cell damage and EDX spectra of metal content in the test samples. *P. aeruginosa* with different treatments (A–H) and *S. aureus* with different treatments (I–P). *P. aeruginosa* and *S. aureus* cells with untreated (A & I), D2A21 treated (B & J), D2A10 treated (C & K), D4E1 treated (D & L), positive control (heat treatment in boiling water bath for 30 min) (E & M), nanocomposites D2A21–AgNPs (F & N), D2A10–AgNPs (G & O), D4E1–AgNPs (H & P). The red coloured arrows indicating the damage of cell wall and white coloured arrows indicating the presence of silver.^114^

A prime example of this is the study conducted by Muchintala *et al.* (2020), which highlights the development of cecropin peptide-based silver nanocomposites (P–AgNPs).^114^ These composites exhibited significantly enhanced antibacterial activity compared to native peptides and silver nanoparticles alone. The study demonstrated that the P–AgNPs had a minimum inhibitory concentration (MIC) as low as 1 μg mL^−1^, showing potent activity against both Gram-positive and Gram-negative bacteria. The size of the nanoparticles, which ranged from 3 ± 0.4 to 20 ± 5 nm, played a critical role in enhancing their antimicrobial efficacy. Smaller particles increase cellular contact, resulting in higher bioavailability and more effective antimicrobial action. Furthermore, the P–AgNPs exerted their antibacterial effect through a membrane disruption mechanism. The study utilized scanning electron microscopy (SEM) and membrane potential analyses, which revealed that the nanocomposites compromised the integrity of bacterial cell membranes, leading to the leakage of cellular contents and ultimately causing bacterial cell lysis. Fig. 9, further illustrate the extent of bacterial membrane damage induced by the P–AgNPs, emphasizing the potential of these composites as effective antibacterial agents.

The mode of action of the cecropin peptide-based silver nanocomposites involves direct interactions with the bacterial cell membrane, where the peptides target the lipopolysaccharide layer, leading to increased membrane permeability. This disruption results in the loss of essential ions like K^+^ and PO_4_^3−^, and the leakage of larger cellular components such as DNA and RNA, contributing to cell death. In particular, the P–AgNPs significantly altered the potassium ion gradient, a hallmark of membrane damage. The study by Muchintala *et al.*^114^ also emphasizes the role of the outer membrane permeabilization, a critical feature of cationic antimicrobial peptides, in the antibacterial activity of P–AgNPs. This was confirmed by the enhanced uptake of NPN dye, indicating membrane disruption, and the subsequent diffusion of silver nanoparticles into bacterial cells, which ultimately resulted in their death.

Further corroborating the antimicrobial potential of peptide-based nanoparticles, Cresti *et al.* (2022) reported on the development of an aerosolized nanosystem encapsulating the antimicrobial peptide SET-M33 within poly(lactide-*co*-glycolide) (PLGA) nanoparticles conjugated with polyethylene glycol (PEG).^115^ This system was designed for enhanced delivery to the lungs and for prolonged antibacterial activity. The study demonstrated that the encapsulated peptide exhibited controlled release, maintaining its antibacterial activity for up to 72 hours after application, with reduced toxicity compared to the free peptide. Importantly, the system showed significant antibacterial efficacy against *Pseudomonas aeruginosa*, both in its planktonic and biofilm forms, showcasing the potential of peptide-loaded nanoparticles in combating persistent infections. These findings underscore the growing significance of peptide-based nanoparticles as versatile and effective antibacterial agents, especially in the context of treating biofilm-associated infections and reducing the risks of toxicity.

The combined antibacterial action of peptides and nanoparticles is thus a powerful approach, with nanoparticles not only enhancing the stability and controlled release of peptides but also amplifying their antimicrobial activity through targeted interactions with bacterial membranes. As evidenced by the findings of Muchintala *et al.* (2020), the synergy between peptides like cecropins and silver nanoparticles offers a promising strategy to tackle multidrug-resistant bacterial infections, potentially overcoming the limitations of traditional antibiotics.^114^

Cresti *et al.* (2022) also developed a biocompatible nanosystem aimed at improving the delivery of the antimicrobial peptide SET-M33 to the lungs *via* aerosol, highlighting its potential for addressing bacterial infections in pulmonary environments.^115^ By encapsulating SET-M33 within poly(lactide-*co*-glycolide) (PLGA) nanoparticles conjugated with polyethylene glycol (PEG), the study achieved a formulation with prolonged antibacterial activity and reduced toxicity compared to the free peptide. The encapsulated peptide displayed robust bactericidal effects against *Pseudomonas aeruginosa*, both in planktonic and sessile forms, and remained active for up to 72 hours post-application. Importantly, the nanosystem exhibited no cytotoxicity when tested on human bronchial epithelial cells, including cells derived from cystic fibrosis patients, whereas the free peptide demonstrated a significantly lower safety threshold. *In vivo* acute toxicity studies further reinforced the nanosystem's safety profile, demonstrating its ability to mitigate the toxic effects commonly associated with free SET-M33. This study underscores the potential of peptide-encapsulated nanoparticles to address respiratory infections effectively while ensuring biocompatibility and controlled peptide release.

Complementing these findings, Serna *et al.* (2017) introduced an innovative protein-only nanoparticle system leveraging the antimicrobial properties of the GWH1 peptide.^116^ By engineering GWH1 to self-assemble into nanoparticles of approximately 50 nm, this study demonstrated broad-spectrum antibacterial activity against Gram-positive and Gram-negative bacteria, without detectable toxicity to mammalian cells. This approach integrated the functional versatility of AMPs with the advantageous properties of nanoscale materials, including biocompatibility, biodegradability, and environmental safety. Unlike traditional AMPs, which often face challenges such as instability, short half-life, and potential toxicity, the GWH1-based nanoparticles exhibited enhanced structural stability and intrinsic microbicidal activity. The single-step biofabrication method employed for these nanoparticles not only streamlined their production but also expanded the potential for scalable manufacturing, paving the way for wider clinical applications. Serna *et al.*'s findings emphasize the potential of protein-only PBNPs as a sustainable and highly effective platform for combating bacterial infections, especially in scenarios where traditional antibiotics fail.

The study by Water *et al.* (2015) explores a novel approach to addressing intracellular *Staphylococcus aureus* infections using plectasin-loaded poly(lactic-*co*-glycolic acid) (PLGA) nanoparticles.^117^ Plectasin, a cationic antimicrobial peptide of the defensin class, was encapsulated in PLGA nanoparticles through the double emulsion solvent evaporation method. This process achieved high encapsulation efficiencies between 71% and 90%, with the nanoparticles providing sustained release of plectasin over 24 hours. The encapsulated formulation demonstrated enhanced antimicrobial efficacy compared to free plectasin when tested on bronchial epithelial Calu-3 cell monolayers infected with *S. aureus*. Importantly, the nanoparticles were shown to maintain eukaryotic cell viability, highlighting their biocompatibility at the concentrations studied.

The intracellular delivery of the nanoparticles was assessed across various cell lines, revealing distinct patterns of uptake. In Calu-3 epithelial cells and THP-1 macrophages, the nanoparticles exhibited significant intracellular localization in punctate patterns, whereas uptake in A549 alveolar epithelial cells was limited. This difference is attributed to variations in cellular endocytosis mechanisms, with clathrin-mediated endocytosis being more prominent in Calu-3 cells. Additionally, confocal microscopy demonstrated that the nanoparticles successfully escaped the endo-lysosomal pathway, enabling direct access to intracellular bacteria. This ability to evade lysosomal degradation is particularly advantageous for combating intracellular pathogens like *S. aureus*, which often reside in the cytoplasm of host cells.

The study also highlighted the optimization of drug loading for effective delivery. While a theoretical drug loading of 2.5% was investigated, a maximum actual loading of 1.8% was achieved, likely due to saturation effects at the oil–water interface and the localization of plectasin on the nanoparticle surface. This surface localization contributed to a burst release profile, which played a role in the initial antimicrobial activity. However, prolonged release formulations with lower burst effects showed reduced overall bacterial killing over 24 hours. The relationship between drug loading, release kinetics, and therapeutic efficacy underscores the need for careful optimization to achieve balanced and sustained intracellular drug delivery.

Overall, the encapsulation of plectasin into PLGA nanoparticles represents a promising strategy for enhancing the intracellular delivery and efficacy of antimicrobial peptides in treating bacterial infections within epithelial tissues. The findings suggest that optimized nanoparticle formulations can improve therapeutic outcomes while maintaining cell safety. Further studies, particularly *in vivo*, are necessary to confirm these results and to explore potential applications in clinical settings.

He *et al.* (2018) designed TAT-modified cationic peptide PA-28 nanoparticles, approximately 150 nm in size, which demonstrated potent antimicrobial activity against both Gram-positive and Gram-negative bacteria, including drug-resistant strains.^118^ These nanoparticles surpassed the efficacy of their unassembled peptide counterpart, nonalysine (K9), by directly disrupting bacterial membranes, thereby minimizing the likelihood of resistance development. Notably, *in vivo* studies revealed the nanoparticles' ability to cross the blood–brain barrier and inhibit bacterial growth in infected rat brains while inducing minimal hemolysis at therapeutic concentrations, highlighting their safety and potential as effective antibacterial agents.

Pal *et al.* (2016) investigated the conjugation of antimicrobial peptides (AMPs) with nanoparticles to enhance antimicrobial efficacy.^119^ They studied cysteine-containing AMPs conjugated with silver nanoparticles, observing dynamic interactions between the components that enhanced stability and biological activity. Using NMR spectroscopy and other biophysical methods, the study elucidated molecular-level interactions, providing insights into the design of AMP-nanoparticle conjugates with superior functionality. This approach highlights the potential for synergy between nanoparticles and peptides in combating resistant bacteria.

Choudhury *et al.* (2024) synthesized chitosan-functionalized gold nanoparticles (ChAuNPs) modified with a lipopolysaccharide-targeting peptide, exhibiting strong antibacterial activity against *Escherichia coli*.^120^ The combination of peptides and laser irradiation at 450 nm yielded synergistic antibacterial effects. The study emphasized the role of advanced nanomaterials, such as chitosan and gold nanoparticles, in enhancing antibacterial functions, even at low concentrations. These findings underscore the potential of targeted modifications in creating robust antibacterial agents.

Chen *et al.* (2024) addressed the challenge of poor cellular permeability in peptide nucleic acids (PNA) by developing extracellular vesicle (EV)-coated mesoporous silicon nanoparticles (MSNs) for enhanced delivery.^121^ The EV-coated nanoparticles showed high encapsulation efficiency and selective uptake by *Staphylococcus aureus*, resulting in superior antibacterial activity compared to free PNA. This biomimetic strategy demonstrated the potential of EV-based delivery systems for improving the therapeutic efficacy of traditionally ineffective or resistance-prone antibiotics.

Dai *et al.* (2023) introduced host-defense peptide-mimicking polymer nanoparticles (TPE-Parg) designed to target intracellular bacterial infections.^122^ These nanoparticles combined antibacterial activity with pro-inflammatory immunomodulatory effects, effectively eradicating intracellular *S. aureus*. By leveraging cell-penetrating peptide-inspired mechanisms, the nanoparticles exhibited low cytotoxicity, induced macrophages to produce nitric oxide, and activated the immune system. *In vivo* studies further confirmed their efficacy in a mouse model of intracellular bacterial infection, positioning them as promising candidates for innovative intracellular antibacterial therapies.

Collectively, these studies highlight the diverse strategies being employed to overcome antibiotic resistance, from designing self-assembling peptides and AMP-nanoparticle conjugates to developing biomimetic and polymer-based nanoparticles. These advancements offer significant promise for addressing the global challenge of drug-resistant bacterial infections.

## 4 Public health implications of peptide-based nanoparticles in antimicrobial therapy

The application of peptide-based nanoparticles (PBNPs) in antimicrobial therapy has significant implications for public health, addressing a variety of pressing concerns that impact healthcare systems worldwide. One of the most critical public health challenges today is the rise of antibiotic-resistant infections. The increasing prevalence of bacteria resistant to multiple drugs has become a global epidemic, threatening to undermine decades of progress in treating infectious diseases.^103^ This growing resistance not only jeopardizes the treatment of common infections but also complicates the management of diseases that were previously considered easily treatable. In this context, PBNPs present a novel solution that could revolutionize antimicrobial therapy by enhancing the effectiveness of existing antibiotics, offering new mechanisms of action, and potentially reducing the emergence of resistance. The versatility of PBNPs allows them to target and treat infections in ways that conventional antibiotics cannot, leading to more effective strategies for managing bacterial infections and reducing the likelihood of resistance development.^105,106^

PBNPs work through various mechanisms that make them effective against resistant bacteria. Traditional antibiotics often target specific bacterial structures or processes, such as cell wall synthesis or protein synthesis, which bacteria can evade through mutation or acquisition of resistance genes. However, PBNPs, which combine the antimicrobial properties of peptides with the enhanced stability and controlled release properties of nanoparticles, offer a different approach. Peptides can exert broad-spectrum antimicrobial activity, disrupting bacterial membranes, interfering with vital processes such as protein and DNA synthesis, and even targeting specific bacterial proteins.^92,97^ When these peptides are encapsulated in nanoparticles, they not only benefit from the nanoparticles' ability to enhance stability and bioavailability but also gain the advantage of controlled release. This control allows the antimicrobial agents to be delivered more effectively to the infection site, reducing the chances of resistance development. Furthermore, nanoparticles themselves can often directly interact with bacterial cells, potentially rendering bacteria more susceptible to the peptide's antimicrobial effects. By providing these alternative mechanisms of action, PBNPs can circumvent traditional antibiotic resistance pathways, making them a valuable addition to the arsenal against resistant infections.^83^

The ability of PBNPs to mitigate antibiotic resistance extends beyond simply enhancing the effectiveness of existing antibiotics. In some cases, PBNPs can function as stand-alone antimicrobial agents, independent of antibiotics. This characteristic is especially important given the alarming rates of multidrug-resistant (MDR) infections. For example, certain peptide-based nanoparticles have been shown to be effective against a wide range of bacteria, including those resistant to multiple classes of antibiotics. This broad-spectrum activity is crucial in a time when the number of infections caused by multidrug-resistant organisms is on the rise. By reducing the reliance on traditional antibiotics and offering a powerful alternative that bacteria are less likely to develop resistance against, PBNPs can contribute to preserving the efficacy of antibiotics and help combat the growing problem of antibiotic resistance.^72,73^

Beyond mitigating antibiotic resistance, PBNPs also improve treatment outcomes in clinical settings. One of the challenges of antimicrobial therapy is ensuring that drugs reach their intended site of action in sufficient concentrations to be effective. Many antimicrobial agents suffer from poor bioavailability, rapid degradation in the body, or difficulty in penetrating bacterial biofilms. These limitations can result in suboptimal dosing and treatment failure, particularly in severe infections or infections caused by multidrug-resistant bacteria. PBNPs can address these challenges by improving the pharmacokinetics of antimicrobial agents. Nanoparticles can be engineered to enhance the solubility and stability of the peptides they carry, allowing for better distribution within the body and sustained drug release over time. This sustained release not only ensures that the antimicrobial agent maintains effective concentrations over a longer period but also reduces the frequency of administration, improving patient compliance.^51,63^

Moreover, PBNPs can be designed to target specific tissues or organs, enhancing the precision of the treatment. This targeted delivery is particularly important in infections that are difficult to treat with conventional antibiotics, such as those affecting the lungs, brain, or other hard-to-reach areas. The surface properties of nanoparticles can be modified with ligands or antibodies that recognize and bind to specific receptors on the surface of infected cells, allowing the PBNPs to accumulate at the infection site. This targeted approach minimizes systemic side effects and maximizes the therapeutic effects at the infection site. In addition to improving treatment efficacy, PBNPs can also help reduce the toxicity associated with conventional antibiotics, which can often cause significant side effects, especially when used over long periods.^67–69^

By enhancing the delivery and potency of antimicrobial agents, PBNPs have the potential to significantly improve clinical outcomes for patients. Infections that were once considered difficult to treat or even untreatable due to resistance could become more manageable, leading to faster recovery times and fewer complications. In patients with chronic or recurrent infections, PBNPs could provide a long-term solution, reducing the need for repeated courses of antibiotics and lowering the risk of adverse effects. For individuals with weakened immune systems, such as those undergoing chemotherapy or organ transplants, PBNPs could offer a safer and more effective means of combating infections, improving their quality of life and overall health outcomes.^34,35^

In addition to improving individual patient outcomes, the use of PBNPs in antimicrobial therapy can also have a profound impact on the healthcare system as a whole. The global healthcare burden posed by antibiotic-resistant infections is immense. The World Health Organization has warned that if antibiotic resistance continues to rise unchecked, by 2050, infections from drug-resistant bacteria could result in 10 million deaths annually, more than cancer. Resistant infections lead to longer hospital stays, more intensive care, and the need for more expensive treatments, significantly increasing healthcare costs. The development and implementation of effective therapies, such as PBNPs, could help alleviate some of this burden. By reducing the incidence of treatment failure and preventing the spread of resistant infections, PBNPs could lower hospitalization rates and reduce the need for expensive interventions. In addition, the use of PBNPs could shorten the duration of hospital stays for patients who require antimicrobial treatment, further reducing the strain on healthcare resources.^108,109^

Reducing the healthcare burden is particularly important in resource-limited settings, where access to advanced medical treatments may be limited. In these areas, the ability to effectively treat infections without relying on expensive antibiotics or complex medical interventions could have a significant impact on public health outcomes. PBNPs, with their potential for broad-spectrum activity, low toxicity, and cost-effective production, could be particularly beneficial in these settings. By providing an alternative to traditional antibiotics, PBNPs could help bridge the gap in access to effective antimicrobial treatments, ensuring that people in all regions of the world can receive the care they need to recover from infections.^25,27^

The implementation of PBNPs in antimicrobial therapy could also have far-reaching implications for global health policies and strategies. As antibiotic resistance continues to rise, many countries are exploring alternative approaches to combat resistant infections. The World Health Organization and other global health bodies have called for urgent action to address the growing threat of antimicrobial resistance, including the development of new antibiotics and alternative therapies. PBNPs align with these goals by offering a novel strategy for managing resistant infections. By integrating PBNPs into national and international healthcare guidelines, public health systems could provide more comprehensive and effective solutions to the problem of antibiotic resistance.^122^

Furthermore, the application of PBNPs in antimicrobial therapy could contribute to a more sustainable approach to public health. The overuse and misuse of antibiotics have played a significant role in the development of resistance. By offering alternative treatments that reduce the need for traditional antibiotics, PBNPs could help slow the spread of resistance and preserve the effectiveness of existing antibiotics for future generations. This aligns with the principles of antimicrobial stewardship, which aims to optimize the use of antimicrobial agents to preserve their effectiveness while minimizing the risk of resistance.^26,56^

In general, the application of PBNPs in antimicrobial therapy holds significant promise for addressing some of the most critical public health challenges of our time. By mitigating antibiotic resistance, improving treatment outcomes, and reducing the healthcare burden associated with multidrug-resistant infections, PBNPs offer a multifaceted solution to the growing crisis of antimicrobial resistance. As research in this field continues to advance, the potential of PBNPs to revolutionize antimicrobial therapy and improve public health outcomes becomes increasingly clear. With continued innovation and investment in the development of PBNPs, the future of antimicrobial therapy could be transformed, providing better outcomes for patients and reducing the global healthcare burden caused by resistant infections.

## 5 Protein-based nanoparticles in cancer therapy

### 5.1 Mechanisms of action

#### 5.1.1 Targeted drug delivery

Peptide-based nanoparticles (PBNPs) offer a novel approach to cancer therapy through targeted drug delivery. Cancer cells often express specific receptors or antigens that are either overexpressed or uniquely present on their surface, which are distinct from those found on normal cells. This characteristic allows for the design of PBNPs with peptides or ligands that specifically bind to these tumor-specific markers, ensuring that the nanoparticles selectively accumulate in cancerous tissues. Such specificity significantly reduces the systemic distribution of drugs and minimizes the toxicity to healthy cells, a common side effect associated with conventional chemotherapy.^123,124^

The selective targeting of cancer cells by PBNPs enhances the overall efficacy of the drug being delivered, as the concentration of the therapeutic agent is higher in the tumor tissue compared to healthy tissues. Additionally, PBNPs can be designed to carry a variety of therapeutic agents, including small molecules, proteins, or RNA-based therapeutics. This flexibility allows for a tailored approach to treatment based on the specific needs of the patient and the characteristics of the tumor. Furthermore, the use of PBNPs can reduce the amount of drug required for effective treatment, lowering the risk of side effects and potentially improving the patient's quality of life during therapy.^21,125^

Cheng *et al.* (2018) combination therapeutic regimen is becoming a primary direction for current cancer immunotherapy to broad the antitumor response.^126^ Functional nanomaterials offer great potential for steady codelivery of various drugs, especially small molecules, therapeutic peptides, and nucleic acids, thereby realizing controllable drug release, increase of drug bioavailability, and reduction of adverse effects. Herein, a therapeutic peptide assembling nanoparticle that can sequentially respond to dual stimuli in the tumor extracellular matrix was designed for tumor-targeted delivery and on-demand release of a short d-peptide antagonist of programmed cell death-ligand 1 (DPPA-1) and an inhibitor of indoleamine 2,3-dioxygenase (NLG919). By concurrent blockade of immune checkpoints and tryptophan metabolism, the nanoformulation increased the level of tumor-infiltrated cytotoxic T cells and in turn effectively inhibited melanoma growth. To achieve this, an amphiphilic peptide, consisting of a functional 3-diethylaminopropyl isothiocyanate (DEAP) molecule, a peptide substrate of matrix metalloproteinase-2 (MMP-2), and DPPA-1, was synthesized and coassembled with NLG919. The nanostructure swelled when it encountered the weakly acidic tumor niche where DEAP molecules were protonated and further collapsed due to the cleavage of the peptide substrate by MMP-2 that is highly expressed in tumor stroma. The localized release of DPPA-1 and NLG919 created an environment which favored the survival and activation of cytotoxic T lymphocytes, leading to the slowdown of melanoma growth and increase of overall survival. Together, this study offers new opportunities for dual-targeted cancer immunotherapy through functional peptide assembling nanoparticles with design features that are sequentially responsive to the multiple hallmarks of the tumor microenvironment.

#### 5.1.2 Controlled release

Another significant advantage of PBNPs in cancer treatment is their ability to facilitate controlled release of therapeutic agents. Traditional chemotherapy often involves administering large doses of drugs over short periods, which can lead to harmful peaks in drug concentration, causing toxicity in healthy tissues. In contrast, PBNPs can be engineered to release their payloads in a controlled manner, maintaining therapeutic drug levels over extended periods. This sustained release ensures that the drug remains effective throughout the treatment cycle, potentially increasing its therapeutic efficacy.^24,127^

The release of the therapeutic agent from PBNPs can be controlled through various mechanisms, such as changes in pH, temperature, or enzymatic activity. These triggers are often associated with the tumor microenvironment, allowing for the release of drugs specifically in the cancerous area. For example, the acidic pH commonly found in tumors can be used to trigger the release of drugs encapsulated within PBNPs. By designing nanoparticles that respond to these specific environmental factors, PBNPs provide a more efficient and effective way to deliver drugs to tumor cells, improving the overall treatment outcome. Additionally, controlled release allows for fewer doses to be administered, which can lead to better patient compliance and a reduced burden on healthcare systems.^128,129^

For instance, Cao *et al.* (2019) demonstrated the use of a smart drug carrier based on a surfactant-like peptide, Nap-FFGPLGLARKRK, for enzyme-triggered cancer-targeted drug delivery.^130^ This peptide, designed with three functional motifs—an aromatic motif for self-assembly, an enzyme-cleavable segment for responsiveness to cancer-overexpressed matrix metalloproteinase-7 (MMP7), and a positively charged segment for cell membrane interaction—showcases how nanostructures can encapsulate high amounts of anticancer drugs such as doxorubicin (DOX). Upon exposure to MMP7 at tumor sites, the peptide nanostructures transition morphologically, enabling the sustained release of DOX and ensuring selective cancer targeting. This approach not only suppresses tumor growth and metastasis but also significantly reduces the side effects of chemotherapy.

**Fig. 10:**
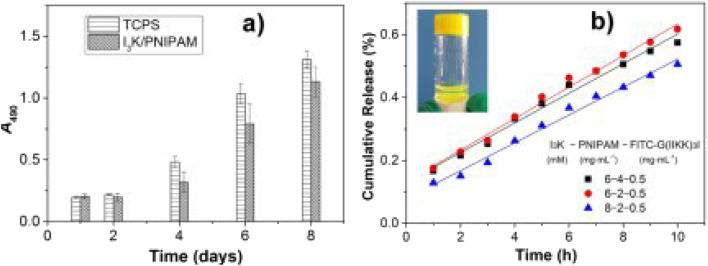
(a) Cytotoxicity of the I_3_K/PNIPAM hydrogel against NIH 3T3 cells as determined by the MTT assay. (b) The cumulative release of FITC-G(IIKK)_3_I-NH_2_ as a function of time.^130^

The effectiveness of such systems is further highlighted by their resemblance to extracellular matrix structures, which enhances biocompatibility. Temperature-responsive hydrogels, for example, offer additional advantages by facilitating *in situ* gelation triggered by body temperature. As shown in Fig. 10, G(IIKK)_3_I-NH_2_, an antibacterial peptide with high selectivity against microbial and tumor cells, was successfully loaded into a PNIPAM/I3K hydrogel system. The hydrogel transitioned from a freely flowing solution at 25 °C to a stable gel at 40 °C. This transformation, depicted in Fig. 10a and b, underscores the potential of these systems for minimally invasive drug delivery.

The release profiles of therapeutic agents, such as G(IIKK)_3_I-NH_2_, further validate the utility of these hydrogels. Fig. 10b illustrates that the cumulative release of the drug exhibited a nearly linear dependence on time after an initial burst, consistent with other PNIPAM-derived hydrogels. The stability of the hydrogel matrix, even after extended exposure to aqueous environments (as seen in the inset of Fig. 10b), ensures prolonged drug release and minimizes the risk of premature degradation.

Moreover, the antibacterial efficacy of the released G(IIKK)_3_I-NH_2_ was confirmed against several bacterial strains, including *Bacillus subtilis*, *Pseudomonas aeruginosa*, *Staphylococcus aureus*, and *Escherichia coli*. Fig. 10c–f presents the survival ratios of these bacterial strains as a function of peptide concentration, highlighting the system's potent antimicrobial properties. These results, combined with MTT assay outcomes indicating high cell viability on the hydrogels (Fig. 10a), establish the biocompatibility and efficacy of PBNPs in drug delivery applications.

In addition to enzyme-triggered systems, interactions between the hydrogel matrix and therapeutic agents play a pivotal role in controlling drug release. Small-angle neutron scattering (SANS) and atomic force microscopy (AFM) results, confirm the incorporation of G(IIKK)_3_I-NH_2_ into I3K nanofibrils, which contributed to enhanced stability and controlled release. These findings demonstrate that by fine-tuning the physicochemical properties of PBNPs, it is possible to achieve precise control over drug delivery dynamics, paving the way for advanced therapeutic strategies in cancer treatment.

By harnessing the unique properties of PBNPs, including their responsiveness to environmental triggers and compatibility with biological systems, researchers are advancing toward more efficient and targeted approaches to cancer therapy. The reduction in dosing frequency, improved patient compliance, and minimized systemic toxicity collectively highlight the transformative potential of controlled release systems in modern oncology.

Quazi and Park (2023) highlight the innovation of a peptide drug delivery platform utilizing nanoscale DNA hydrogel (Dgel) functionalized with gold nanoparticles (AuNPs).^131^ This system ensures cancer-specific targeting and minimizes off-target effects by employing a light-triggered mechanism for the precise release of anticancer peptide drugs. The engineered Dgel-PD-AuNP-YNGRT complex demonstrated exceptional efficacy, achieving up to 90% peptide drug release and significantly higher cancer cell mortality with negligible impact on healthy tissues. This advancement underscores the potential of PBNPs to mitigate the challenges of enzymatic degradation and unwanted leakage associated with peptide drugs.

Guo *et al.* (2024) further expanded on the versatility of PBNPs by introducing a nanoparticle system incorporating heptamethine cyanine molecules, such as IR808, which can indiscriminately target a wide array of tumor types.^132^ By integrating tumor-targeted delivery, photothermal therapy, and near-infrared imaging, the DSPE-PEG-IR808-based nanoparticles encapsulating paclitaxel (PTX) and an immunomodulator (R848) exhibited remarkable efficacy. The DP-IR808@PTX-R848 nanoparticles achieved a tumor inhibition rate of 94% and prolonged survival in treated models, showcasing their multifaceted role in enhancing therapeutic outcomes while minimizing systemic toxicity. This study emphasizes the strategic design of nanoparticles to leverage inherent tumor microenvironment properties, enabling controlled release and precise targeting.

Similarly, Wu *et al.* (2024) demonstrated the effectiveness of PBNPs in hepatocellular carcinoma (HCC) therapy through the development of SP94-PDA-PHBV-TAX nanoparticles.^133^ This system incorporated a pH-responsive shell that enabled targeted drug release in the acidic tumor microenvironment, while the surface-grafted SP94 peptide facilitated specific binding to HCC cells. The SP94-PDA-PHBV-TAX nanoparticles outperformed free taxol (TAX) in both *in vitro* and *in vivo* models, exhibiting superior antitumor efficacy with reduced systemic toxicity. This underscores the role of PBNPs in providing a biocompatible, stimuli-responsive drug delivery system tailored to specific cancer types.

Collectively, these studies exemplify the transformative impact of PBNPs in cancer therapy by addressing critical challenges such as off-target toxicity, enzymatic degradation, and uncontrolled drug release. Through strategic functionalization and the incorporation of stimuli-responsive mechanisms, PBNPs enable precise and efficient delivery of therapeutic agents, paving the way for safer and more effective cancer treatments.

#### 5.1.3 Tumor microenvironment modulation

The tumor microenvironment (TME) plays a crucial role in the resistance of tumors to treatment. Features such as poor blood supply, low oxygen levels, and an acidic pH make it difficult for conventional therapies to reach and effectively treat cancer cells. PBNPs have the potential to modulate the TME in ways that enhance the efficacy of cancer treatments. Functionalized PBNPs can improve the permeability of the blood-tumor barrier, which typically limits the delivery of drugs to tumor cells. By increasing the permeability of blood vessels in the tumor, PBNPs enable better penetration of chemotherapeutic agents, improving drug delivery and effectiveness.^134,135^

Furthermore, PBNPs can be engineered to target specific receptors or biomarkers on tumor-associated blood vessels, which can help normalize the vasculature within the tumor. This normalization can improve the blood flow within the tumor, making it easier for drugs to reach the cancer cells. Additionally, PBNPs can be designed to carry imaging agents, allowing for real-time monitoring of the tumor and the treatment's progress. This capability not only improves the targeting and delivery of drugs but also enables better treatment planning and optimization, which can ultimately lead to improved patient outcomes.^136^

For example, Zhang and Xu (2023) emphasize that the interactions between tumor cells and stromal cells within the TME significantly influence cancer progression and treatment resistance.^137^ Stromal cells in the TME, including angiogenic vascular cells, infiltrating immune cells, and fibrosis-related cells, not only support tumor growth but also contribute to the inefficiency of drug delivery by forming barriers and creating a hostile microenvironment. Therapeutic peptides, which can be self-assembled or combined with polymeric molecules to form nanoparticles, have demonstrated remarkable capabilities in targeting these stromal cells and modulating the TME.

PBNPs can improve the permeability of the blood-tumor barrier, a major obstacle in cancer therapy. Functionalized PBNPs are engineered to target specific receptors or biomarkers on tumor-associated blood vessels, leading to normalization of the tumor vasculature. This normalization enhances blood flow within the tumor, ensuring improved delivery of chemotherapeutic agents. By increasing vascular permeability, PBNPs enable more effective penetration of drugs into the tumor, addressing one of the critical limitations of traditional therapies.

Additionally, PBNPs can be designed to respond to the unique conditions of the TME, such as pH, hypoxia, and specific enzymatic activities. These stimuli-responsive properties allow for the controlled and localized release of therapeutic agents, minimizing off-target effects and reducing toxicity. For instance, peptides integrated within PBNPs can recognize cancer-specific markers overexpressed in tumor cells and stromal components, ensuring targeted delivery. This targeted approach not only enhances the therapeutic efficacy of the encapsulated drugs but also mitigates the adverse effects on normal tissues.

**Fig. 11:**
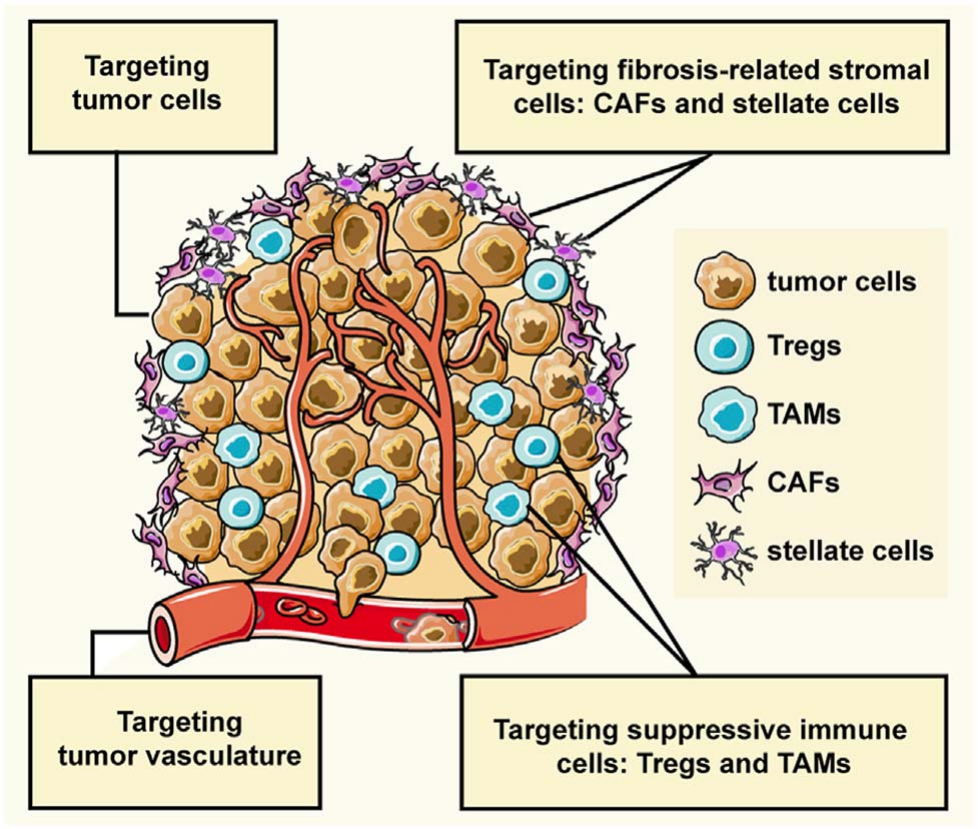
Interactions within the tumor microenvironment (TME), comprising tumor cells and three major classes of stromal cells—angiogenic vascular cells, infiltrating immune cells, and tumor fibrosis-related cells—significantly influence cancer progression. This figure illustrates the peptide-assembled nanoparticles designed to target tumor cells and stromal cells for enhanced cancer therapy.^137^

Fig. 11 provides a visual representation of the intricate interactions within the TME. It highlights how tumor cells interact with the three main classes of stromal cells—angiogenic vascular cells, infiltrating immune cells, and tumor fibrosis-related cells—to sustain the tumor's growth and survival. PBNPs are designed to disrupt these interactions by targeting each stromal cell type. For example, they can inhibit the pro-tumor activities of suppressive immune cells, such as tumor-associated macrophages, while normalizing the vasculature to facilitate improved drug delivery.

Another critical advantage of PBNPs is their ability to integrate diagnostic and therapeutic functionalities. By carrying imaging agents, these nanoparticles enable real-time monitoring of drug distribution and therapeutic progress. This theranostic capability allows clinicians to adapt treatment strategies dynamically, optimizing outcomes for patients. The ability to visualize and track PBNP behavior within the TME further enhances the precision of cancer therapy.

In summary, Zhang and Xu (2023) underscore the transformative potential of PBNPs in cancer treatment by focusing on their ability to modulate the TME.^137^ By targeting tumor cells and the associated stromal cells, these nanoparticles overcome the barriers posed by the TME, improve drug delivery, and enable real-time monitoring.

Gong *et al.* (2021) demonstrated how functionalized PBNPs can be utilized to improve drug delivery and therapeutic outcomes by specifically targeting and responding to the unique features of the TME.^138^

In this study, a pH-responsive drug-loading system was developed, leveraging target peptide ligands to achieve precise drug delivery and release at the tumor site. The system utilized the enhanced permeability and retention (EPR) effect for passive targeting of tumor tissue and incorporated active targeting mechanisms *via* RGD motifs. These motifs allowed the nanoparticles to bind integrins, such as ανβ3, which are overexpressed on the surface of tumor cells, thus enabling specific and efficient tumor cell targeting. By combining passive and active targeting approaches, these PBNPs overcame the challenges posed by the blood-tumor barrier and facilitated improved penetration of chemotherapeutic agents into the tumor.

**Fig. 12:**
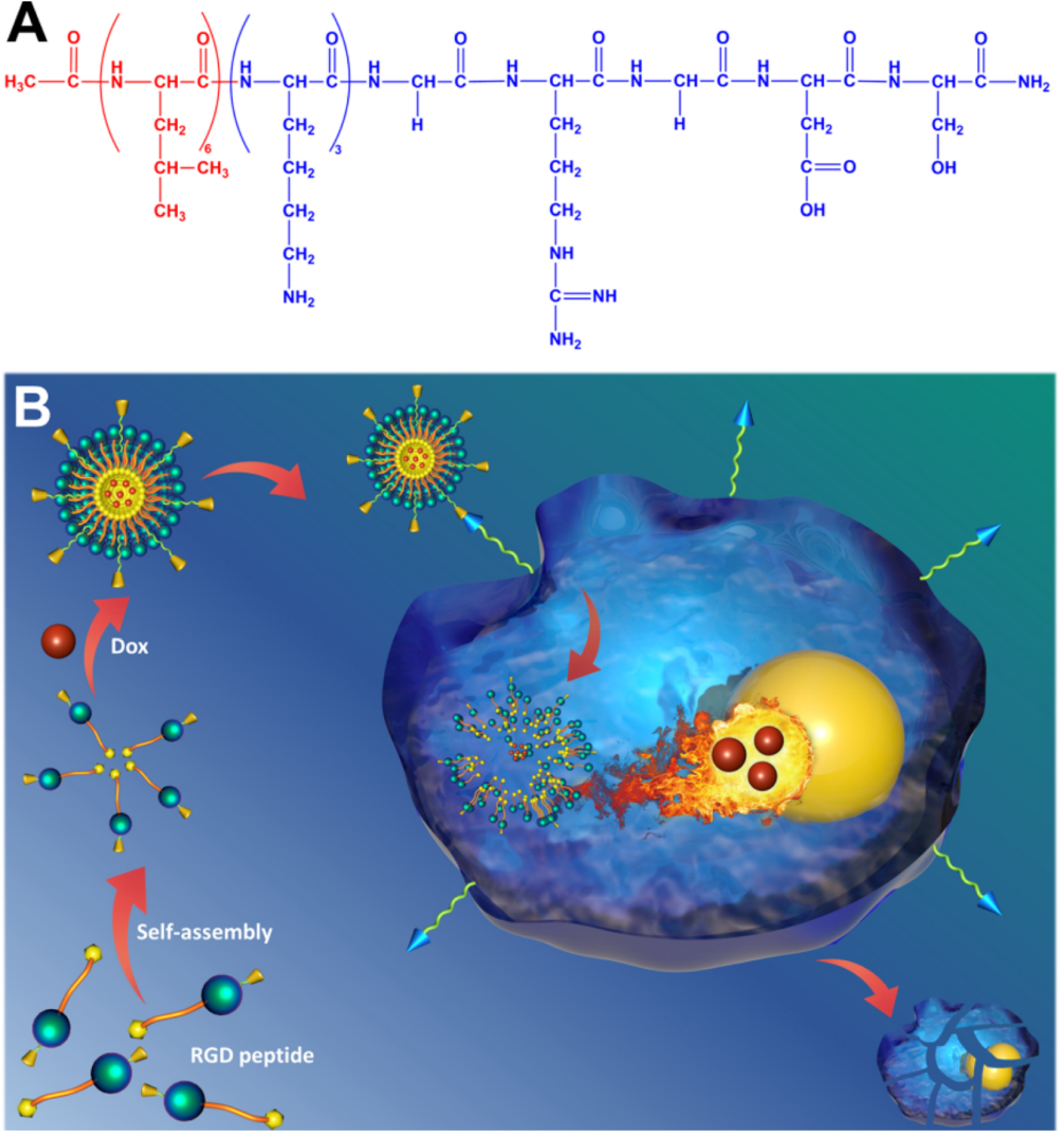
(A) Molecular structures of the RGD peptide LKR. The red represents hydrophobic areas, and the blue represents hydrophilic areas. (B) Self-assembly behavior and acid-responsive morphological transformation of LKR. After the targeted nanoparticles reached the tumor site, the slightly acidic tumor environment triggered the rupture of the spherical nanoparticles, resulting in the release of the encapsulated antitumor drug and achieving a combined antitumor effect.^138^

Self-assembling peptides, such as the amphiphilic peptide LKR used in this study, were shown to have significant structural advantages. As depicted in Fig. 12, panel (A) illustrates the molecular structure of the RGD peptide LKR, with hydrophobic areas represented in red and hydrophilic areas in blue. These structural features enabled the peptide to self-assemble into spherical nanoparticles in neutral conditions, encapsulating the fat-soluble antitumor drug doxorubicin (Dox). The nanoparticles were designed to respond specifically to the acidic microenvironment of the TME, as illustrated in Fig. 12B. Upon reaching the tumor site, the slightly acidic environment triggered the swelling and rupture of the spherical nanoparticles, resulting in the controlled release of the encapsulated drug. This acid-responsive transformation allowed for a targeted therapeutic effect, ensuring the drug was delivered locally to the tumor site while minimizing off-target effects and systemic toxicity.

Flow cytometry and fluorescence detection further demonstrated that the self-assembled LKR nanoparticles significantly enhanced drug accumulation in tumor cells compared to normal mammalian cells. This targeted delivery approach increased the local concentration of Dox in the tumor, improving its therapeutic efficacy. Moreover, this strategy addressed a key limitation of traditional chemotherapy by reducing systemic exposure and associated side effects.

*In vivo* experiments reinforced the antitumor efficacy of the Dox-encapsulating peptide nanoparticles. By exploiting the acidic pH of the TME, the nanoparticles not only achieved localized drug release but also facilitated the normalization of tumor blood flow. This normalization improved the distribution of the therapeutic agents within the tumor, further enhancing their effectiveness. The modulation of the TME through this mechanism highlights the potential of PBNPs to optimize drug delivery and therapeutic outcomes.

The integration of passive and active targeting mechanisms, combined with the pH-responsive behavior of the nanoparticles, underscores the transformative potential of PBNPs in cancer therapy. Gong *et al.* (2021) provided a compelling example of how these nanoparticles can address the limitations of conventional treatments, particularly in challenging cancers such as hepatoma.^138^ By enhancing drug delivery, improving tumor penetration, and enabling localized drug release, PBNPs hold significant promise for modulating the TME and achieving better therapeutic outcomes. This study aligns with the broader potential of PBNPs to revolutionize cancer treatment, offering a platform for more effective, targeted, and patient-centered therapeutic strategies.

#### 5.1.4 Photothermal and photodynamic therapy

Peptide-based nanoparticles (PBNPs) offer significant potential for combination therapies in cancer treatment, particularly when integrated with advanced modalities like photothermal therapy (PTT) and photodynamic therapy (PDT). These approaches leverage the unique properties of light-activated processes to achieve precise, localized tumor destruction while minimizing harm to surrounding healthy tissues.^139,140^

Photothermal therapy relies on the conversion of light energy into heat, which raises the temperature of tumor tissues to induce cell death. PBNPs, including those composed of materials such as ferritin, excel in this application due to their ability to absorb specific wavelengths of light and convert it into localized hyperthermia. This targeted heating effect is critical in selectively damaging cancer cells while sparing normal tissues, a hallmark of precision cancer therapy.^140,141^ When combined with tumor microenvironment-responsive systems, such as the tumor acidity-responsive lipid membrane-enclosed nanoparticles described by Kv *et al.* (2020), the efficacy of PTT is further enhanced.^142^ These nanoparticles, PFOB@IMHNPs, are engineered to deliver the photothermal agent IR780 into tumor tissues, ensuring effective light-triggered heating and tumor suppression under near-infrared (808 nm) irradiation.

Similarly, photodynamic therapy capitalizes on light-induced activation of photosensitizing agents to produce reactive oxygen species (ROS), which can induce oxidative damage and apoptosis in cancer cells. PBNPs can be functionalized to carry photosensitizers like mTHPC, which, upon exposure to specific light wavelengths, generate ROS in the hypoxic tumor microenvironment. As Kv *et al.* (2020) demonstrated, the PFOB@IMHNPs effectively delivered mTHPC into hypoxic TRAMP-C1 cells and, upon 660 nm irradiation, facilitated significant ROS production, leading to effective cell killing *in vitro*.^142^ This mechanism addresses one of the key limitations of PDT—the oxygen-deficient nature of tumors—by ensuring a self-sufficient oxygen supply through the perfluorooctyl bromide (PFOB) core of the nanoparticles.

The study by Kv *et al.* (2020) further highlights the synergistic potential of combining PTT and PDT using these innovative nanoparticles.^142^ By co-delivering IR780 and mTHPC, along with oxygen transportation capabilities, PFOB@IMHNPs enable dual therapeutic actions in hypoxic tumor environments. This combination therapy demonstrated dramatic suppression of TRAMP-C1 tumor growth *in vivo*, achieving effective tumor-targeted delivery, hypoxia alleviation, and enhanced therapeutic outcomes. The dual-wavelength irradiation strategy (808 nm for PTT and 660 nm for PDT) enabled precise activation of the respective agents, underscoring the versatility of this approach.

**Fig. 13:**
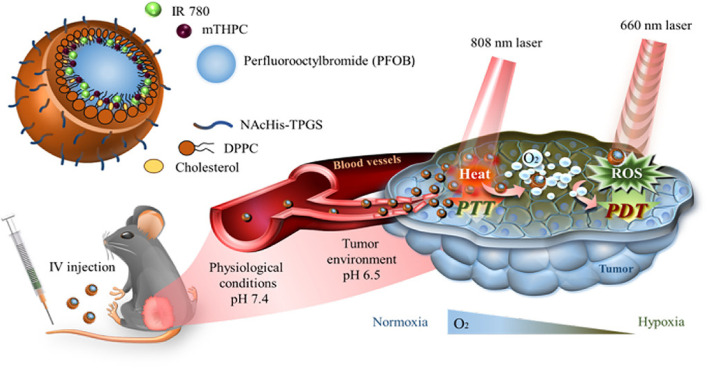
Illustration of tumor microenvironment-responsive, oxygen self-sufficient oil droplet nanoparticles designed for enhanced photothermal and photodynamic combination therapy, effectively addressing hypoxia in tumor treatment.^142^

Fig. 13, which illustrates the design and function of tumor microenvironment-responsive, oxygen self-sufficient oil droplet nanoparticles, effectively contextualizes the mechanisms discussed in the study. The nanoparticles' acid-responsive lipid membrane enhances cellular uptake by generating a surface positive charge in acidic tumor environments, further facilitating the delivery of IR780 and mTHPC. This design ensures efficient activation of both photothermal and photodynamic therapies, directly addressing the hypoxic limitations of conventional approaches and maximizing therapeutic efficacy.

In summary, PBNPs represent a promising platform for integrating photothermal and photodynamic therapies into cancer treatment strategies. The innovative design of PFOB@IMHNPs, as presented in Kv *et al.* (2020)^142^, exemplifies how these nanoparticles can overcome the challenges of tumor-targeted delivery and hypoxia, paving the way for more effective and personalized cancer therapies.

**Scheme 2:**
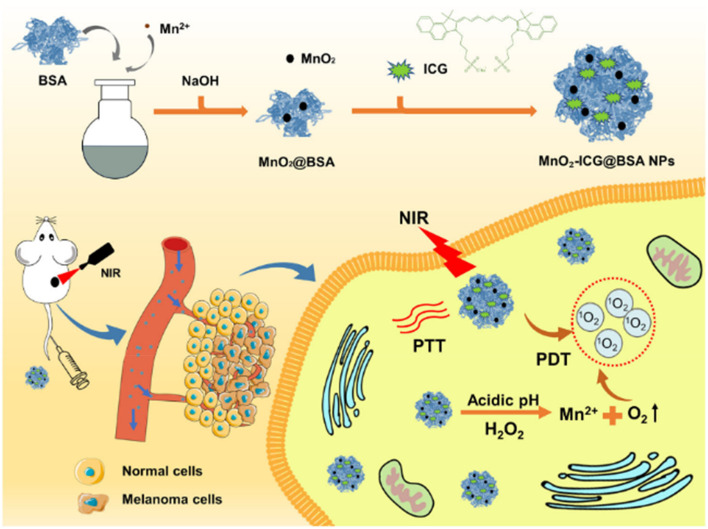
A preparation and application schematic illustrating the PTT–PDT of melanoma mediated by MnO_2_-ICG@BSA under NIR irradiation. NIR, near-infrared; PTT–PDT, photothermal–photodynamic combination therapy.^143^

The integration of PBNPs into PTT–PDT systems offers a synergistic approach, as illustrated in the study by Wen *et al.* (2020).^143^ This research detailed the development of tumor-microenvironment-responsive hydrogen-peroxide-activated protein biomimetic nanoparticles (MnO_2_-ICG@BSA) for treating melanoma. Scheme 2 depicts the preparation and application of these nanoparticles under near-infrared (NIR) irradiation. The MnO_2_-ICG@BSA nanoparticles not only displayed high photothermal stability and a photothermal conversion efficiency of 24.7% but also exhibited robust singlet oxygen production upon NIR laser exposure. This dual functionality enabled a significant reduction in melanoma growth through the combined action of PTT and PDT.

The study also highlighted the ability of MnO_2_-ICG@BSA to alleviate tumor hypoxia by catalyzing hydrogen peroxide into oxygen, a critical aspect of enhancing PDT efficacy. *In vivo* experiments demonstrated the nanoparticles' capacity to improve the tumor microenvironment while achieving a statistically significant reduction in tumor size. Tumor growth was entirely inhibited within eight days post-treatment with MnO_2_-ICG@BSA and NIR irradiation, showcasing its potential as a robust therapeutic candidate.

This multifunctionality of PBNPs aligns with their broader therapeutic potential. By integrating photothermal and photodynamic effects, PBNPs offer a platform for highly targeted and effective cancer treatments. Furthermore, these nanoparticles can be engineered to combine other therapeutic functions, such as targeted drug delivery and tumor microenvironment modulation, creating a holistic approach to cancer therapy.

Overall, the unique properties of PBNPs, including their tunable photothermal and photodynamic characteristics, position them as a cornerstone for future advancements in personalized cancer treatments. With continued refinement and integration into multimodal therapies, PBNPs provide a promising pathway to significantly enhance the efficacy of cancer treatment while reducing systemic side effects.

One remarkable study by Jiang *et al.* (2021) highlights the multifunctional capabilities of PBNPs engineered with aggregation-induced-emission luminogens (AIEgens), specifically DDTB, which exhibit near-infrared fluorescence, photothermal effects, and photodynamic properties.^144^ These nanoparticles demonstrated excellent utility in intraoperative imaging and therapy, allowing real-time tumor visualization during surgical procedures. After tumor resection, residual cancer cells were targeted with combined PTT and PDT. Additionally, by conjugating these nanoparticles with programmed death-ligand 1 (PD-L1) antibodies, the immune system's response to cancer cells was amplified. This combinatory approach significantly enhanced treatment outcomes, with treated animal models achieving a survival rate of up to 90%, showcasing the versatility of PBNPs in enhancing both direct and immune-mediated cancer therapies.

Another innovative development by Liu *et al.* (2021) tackled the challenges posed by the hypoxic microenvironment of tumors, which often undermines the effectiveness of photodynamic therapy.^145^ By developing liquid-metal-based nanoparticles combined with metronidazole and RGD peptides, the researchers engineered a system capable of ROS generation and radiation enhancement under both near-infrared and X-ray activation. The RGD peptides facilitated specific tumor targeting, while the liquid-metal core ensured efficient photothermal conversion. This innovative approach not only overcame the limitations posed by hypoxia but also achieved remarkable tumor volume reductions in experimental models, emphasizing the adaptability of PBNPs in addressing tumor-specific barriers.

The potential of natural compounds incorporated into PBNPs was explored by Ashkbar *et al.* (2020), who synthesized Fe_3_O_4_/SiO_2_–curcumin nanocomposites for breast cancer therapy.^146^ Curcumin, a naturally occurring anticancer agent, acted as a photosensitizer to enhance ROS generation, while Fe_3_O_4_/SiO_2_ nanoparticles provided robust photothermal properties. This dual-function system led to enhanced apoptosis of cancer cells, as evidenced by increased expression of pro-apoptotic markers like Bax and Caspase-3. Significant tumor volume reduction *in vivo* confirmed the efficacy of these nanoparticles, highlighting their ability to synergistically combine oxidative and thermal mechanisms for targeted cancer therapy.

Surface functionalization of PBNPs also plays a pivotal role in optimizing their therapeutic efficacy, as demonstrated by Liu *et al.* (2022).^147^ The researchers designed polydopamine nanoparticles loaded with indocyanine green (ICG), a widely used photosensitizer, and coated them with hyaluronic acid for biocompatibility and tumor-specific targeting. These nanoparticles achieved high photothermal conversion efficiency and significant singlet oxygen generation under light activation. The hyaluronic acid coating enhanced their uptake by cancer cells through receptor-mediated pathways, resulting in effective tumor suppression with minimal off-target effects. This study underscores the importance of functionalizing PBNPs to improve their specificity and safety profiles in clinical applications.

Overcoming inherent resistance mechanisms within tumors is another critical area addressed by PBNPs. Wen *et al.* (2021) focused on mitigating thermoresistance, a common obstacle in photothermal therapy caused by the upregulation of heat shock proteins like Hsp-90.^143^ By developing bovine serum albumin-based nanoparticles loaded with geldanamycin, Cy7, and squaraine dye, the researchers effectively inhibited Hsp-90 activity while delivering robust photothermal and photodynamic effects. This innovative strategy not only enhanced tumor ablation but also addressed a major limitation of PTT, paving the way for more reliable and effective cancer treatments.

Furthermore, Liu *et al.* (2021) introduced diketopyrrolopyrrole-based polymer nanoparticles that combined high photothermal conversion efficiency with moderate ROS production.^147^ The simplicity of their design, requiring activation by a single laser wavelength, made them highly effective in preclinical models. These nanoparticles achieved precise tumor ablation with minimal damage to surrounding healthy tissues, demonstrating the practicality of single-wavelength activation systems in simplifying treatment protocols while maintaining high therapeutic efficacy.

In a nutshell, peptide-based nanoparticles represent a versatile and transformative platform for integrating photothermal and photodynamic therapies in cancer treatment. By leveraging light-activated processes and addressing tumor-specific challenges such as hypoxia and thermoresistance, PBNPs provide a promising avenue for precise and effective cancer therapy. Their ability to combine imaging, therapy, and immune modulation within a single platform highlights their potential to revolutionize oncology, offering new hope for personalized and minimally invasive treatments.

## 6 Key applications

### 6.1 Abraxane®: a paradigm shift in nanoparticle-based chemotherapy for cancer treatment

Abraxane® represents a groundbreaking innovation in cancer treatment, specifically designed to address the limitations of conventional paclitaxel delivery. As an FDA-approved formulation, Abraxane® employs albumin nanoparticles to encapsulate paclitaxel, a widely used chemotherapeutic agent, and has demonstrated efficacy in treating breast, lung, and pancreatic cancers. This approach addresses significant challenges associated with traditional paclitaxel formulations, particularly its poor water solubility and reliance on toxic solvents like Cremophor EL, which are known to cause severe hypersensitivity reactions in patients. By replacing these solvents with biocompatible albumin, Abraxane® enhances patient safety and tolerability, thereby expanding its clinical application.^148,149^

One of the key advantages of Abraxane® lies in its ability to improve the pharmacokinetic and pharmacodynamic profiles of paclitaxel. The use of albumin, a naturally occurring protein, as a nanoparticle carrier facilitates the formation of a stable, water-soluble complex, overcoming paclitaxel's inherent hydrophobicity. This enhances the drug's bioavailability and allows for higher doses to be administered safely. Moreover, the albumin nanoparticles leverage the enhanced permeability and retention (EPR) effect—a hallmark of tumor vasculature—to selectively accumulate in tumor tissues. This targeted delivery minimizes off-target toxicity and maximizes the therapeutic impact on cancer cells, providing a more effective and patient-friendly treatment option.^150–152^

Abraxane®'s mechanism of action also involves albumin's natural interaction with specific receptors, such as gp60 and SPARC (secreted protein acidic and rich in cysteine), which are overexpressed in many tumor cells. This receptor-mediated transcytosis enables efficient transport of the drug across endothelial cells, further enhancing tumor penetration. These attributes collectively contribute to the drug's ability to achieve higher intratumoral concentrations of paclitaxel compared to traditional formulations, leading to improved clinical outcomes in terms of tumor shrinkage, progression-free survival, and overall response rates.^153,154^

The success of Abraxane® not only underscores the potential of protein-based nanoparticles (PBNPs) in oncology but also serves as a model for the development of next-generation nanoparticle therapeutics. Its formulation exemplifies the integration of nanotechnology with molecular biology to overcome the pharmacological challenges of chemotherapeutic agents. Furthermore, the albumin-based platform used in Abraxane® has opened new avenues for designing nanoparticle systems capable of delivering a wide range of hydrophobic drugs with improved efficacy and safety profiles.^155,156^

In addition to its clinical benefits, Abraxane® has set a precedent for the regulatory approval of nanoparticle-based therapies, demonstrating their feasibility and scalability for widespread use. This has paved the way for ongoing research and innovation in nanoparticle formulations, emphasizing the versatility of albumin as a carrier material. As a result, the success of Abraxane® continues to inspire the development of novel therapeutic platforms aimed at addressing unmet needs in cancer treatment, making it a cornerstone in the field of nanomedicine.^152^

For example, the study by Song *et al.* (2022) builds on this paradigm by designing a dual-immunomodulator albumin nanoparticle, Nano-PI, containing PI3Kγ inhibitor (IPI-549) and paclitaxel (PTX).^157^ This innovative approach addresses the immunosuppressive tumor microenvironment (TME) that hinders the efficacy of current immunotherapies, such as α-PD1 or α-PDL1, particularly in triple-negative breast cancer (TNBC). Unlike conventional therapies that primarily target the tumor site, Nano-PI simultaneously modulates the immune microenvironment in both tumors and lymph nodes. This dual-site remodeling is critical for long-term remission, as demonstrated in MMTV-PyMT mouse models. Nano-PI in combination with α-PD1 achieved complete tumor remission and eradicated lung metastases, a feat rarely accomplished in aggressive metastatic breast cancer.

**Fig. 14:**
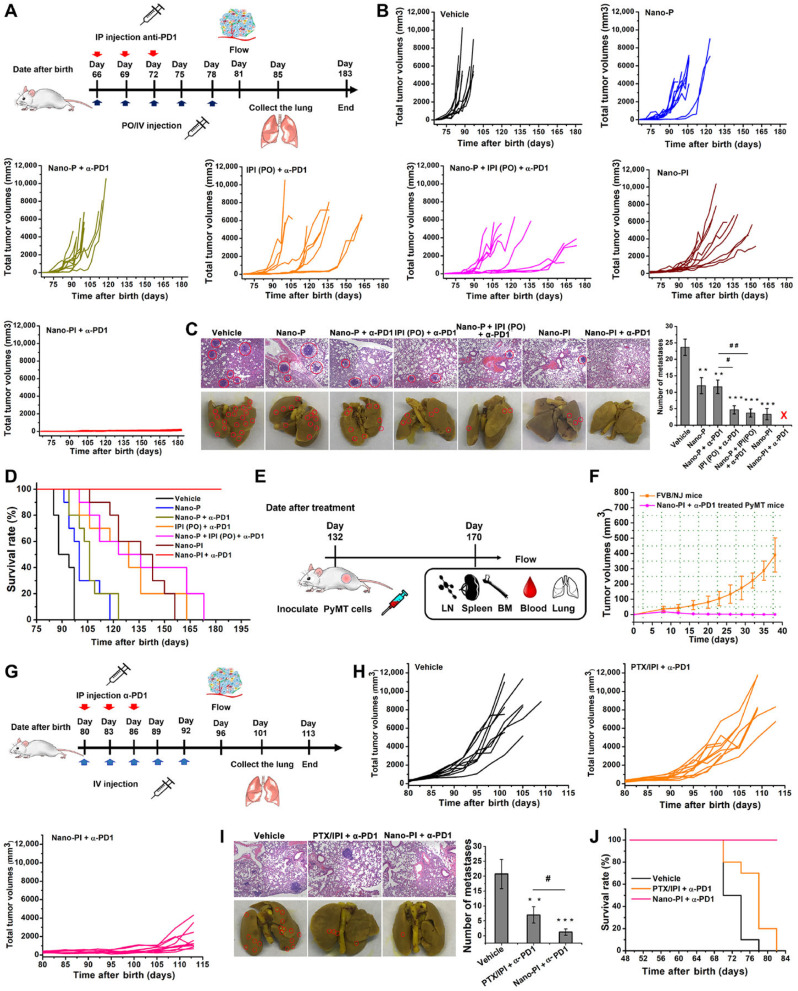
Enhanced antitumor efficacy and survival through combination therapy using Nano-PI and anti–PD-1 in a mouse model of metastatic breast cancer. (A) Schematic representation of the experimental design for treatment of MMTV-PyMT mice with Nano-PI (nanoparticle-loaded PI3K inhibitor), anti–PD-1, or their combination. Mice received intraperitoneal (IP) anti–PD-1 and intravenous (IV) Nano-PI or control treatments starting on day 105 after birth. Lungs were collected at the end of treatment to evaluate tumor burden. (B) Tumor burden progression over time in individual mice treated with vehicle, Nano-P (nanoparticles without drug), anti–PD-1 alone, Nano-PI, or combinations thereof. The combination of Nano-PI + anti–PD-1 showed the most significant inhibition of tumor growth. (C) Representative hematoxylin and eosin (H&E)-stained lung tissue sections and gross lung images showing metastatic tumor nodules across treatment groups. Quantification of metastatic nodules per lung reveals significantly reduced tumor burden in the Nano-PI + anti–PD-1 group. (D) Kaplan–Meier survival curves of treated mice indicate improved survival in the Nano-PI + anti–PD-1 group compared to monotherapies and control. (E) Diagram showing the experimental timeline for immune profiling of Nano-PI + anti–PD-1–treated PyMT tumor-bearing mice. Tumors were inoculated subcutaneously on day 132, and immune organs (lymph nodes, spleen, bone marrow, blood, and lungs) were collected on day 170. (F) Quantification of immune cell populations from flow cytometry in various tissues of treated mice. The combination therapy group exhibited enhanced T-cell infiltration and activation compared to other groups. (G) Schematic of another treatment model and timeline for validation using IV injection of Nano-PI in late-stage tumor-bearing MMTV-PyMT mice. (H) Tumor burden progression in mice treated with vehicle, PTX/PI (paclitaxel and PI3K inhibitor combination), or Nano-PI + anti–PD-1. (I) Representative H&E lung sections and gross lung images show reduced metastasis in Nano-PI + anti–PD-1–treated mice. Bar graphs quantify metastatic nodules. (J) Kaplan–Meier survival curve from validation experiment confirming extended survival in the combination treatment group. Statistical significance is indicated as follows: *p* < 0.05, *p* < 0.01, and *p* < 0.001. Data are presented as mean ± SEM.^157^

Fig. 14 highlights the comprehensive efficacy of this approach, illustrating the significant reduction in tumor volumes and the elimination of metastatic nodules following the combined treatment. The schematic dosing regimen and survival analysis emphasize the sustained therapeutic benefit of Nano-PI and α-PD1 over 180 days. Enhanced drug delivery and immune modulation were evident, with Nano-PI facilitating superior accumulation of IPI-549 and PTX in macrophages within both lymph nodes and tumors. Immune cell profiling further revealed substantial shifts in the TME, including the repolarization of immunosuppressive M2 macrophages to pro-inflammatory M1 macrophages, increased infiltration of effector T cells, and reduced regulatory T cells.

Comparatively, the MARIO-3 clinical trial, which evaluated a PI3Kγ inhibitor with Abraxane® and atezolizumab (α-PDL1), reported promising preliminary results in TNBC patients. However, the outcomes in preclinical and clinical studies suggest that Nano-PI may surpass these combinations due to its enhanced biodistribution and immune remodeling capabilities. The synergy between IPI-549 and PTX in Nano-PI represents a significant improvement over earlier formulations, such as Abraxane®, by addressing both local and systemic immunosuppression, thereby improving therapeutic outcomes.

The manufacturing feasibility of Nano-PI, using established processes similar to those for Abraxane®, further enhances its clinical translational potential. As Nano-PI demonstrated complete tumor remission and metastasis prevention in a spontaneous breast cancer mouse model, its clinical relevance appears robust, warranting further investigation in human trials. This study exemplifies the next generation of PBNPs, where dual-functional nanoparticles like Nano-PI hold the promise of redefining cancer treatment by integrating chemotherapeutic and immunomodulatory strategies into a single, highly effective platform.

O'Connell *et al.* (2024) explores a combination of the PI3K-γ inhibitor eganelisib with atezolizumab and nab-paclitaxel for metastatic triple-negative breast cancer (mTNBC), a notoriously aggressive subtype.^158^ This study underscores the critical role of tumor-associated macrophages (TAMs) in immune evasion within the tumor microenvironment (TME). The inclusion of nab-paclitaxel in the MARIO-3 trial emphasizes its capacity not only as a cytotoxic agent but as a key component in reprogramming the TME to favor immune activation, potentially overcoming TAM-mediated resistance. The detailed translational analyses from MARIO-3 reveal that nab-paclitaxel, in synergy with immune checkpoint inhibitors, achieves immune activation and extracellular matrix reorganization, suggesting a broader mechanistic applicability of albumin-bound nanoparticle formulations in enhancing immunotherapy outcomes.

Building on this, Futamura *et al.* (2021) focuses on nanoparticle albumin-bound paclitaxel (nab-PTX) in neoadjuvant settings for operable breast cancer.^159^ Their meta-analysis demonstrated robust pathological complete response (pCR) rates across various breast cancer subtypes, particularly HER2-rich and TNBC. The superior efficacy observed in aggressive cancers aligns with the findings of O'Connell *et al.*^158^ which highlight nab-PTX's potential to enhance immune responses even in tumors characterized by baseline immunosuppression. Furthermore, the tolerability profile observed by Futamura *et al.*, with manageable adverse effects and recovery in most patients, reinforces nab-paclitaxel's suitability as a backbone for combinatory regimens, bridging its established chemotherapeutic benefits with immunomodulatory applications.

Macarulla *et al.* (2019) extends the discussion to metastatic pancreatic ductal adenocarcinoma (PDAC), another challenging malignancy.^160^ In this context, nab-paclitaxel combined with gemcitabine demonstrated significant survival benefits, even in patients with poor performance status (PS). The tolerability of varied dosing regimens, as demonstrated in this study, mirrors the observations in breast cancer studies, reinforcing the versatility of albumin-bound formulations across diverse cancer types. Moreover, Macarulla *et al.*'s findings about manageable hematological toxicities and durable efficacy provide a foundation for integrating nab-paclitaxel into more complex treatment paradigms, such as those investigated by O'Connell *et al.*^158^

Peralta *et al.* (2015) expands the horizon by demonstrating the encapsulation of gold nanorods (AuNRs) alongside paclitaxel within human serum albumin nanoparticles (HSAPs).^161^ This study highlights the feasibility of creating multifunctional nanoparticles combining chemotherapy with photothermal therapy, thereby addressing limitations in single-modal treatments. While primarily preclinical, the approach offers a potential trajectory for Abraxane®-like formulations, incorporating synergistic mechanisms to enhance therapeutic outcomes. The concept of leveraging the albumin matrix for dual-functional delivery aligns with the clinical strategies discussed by Futamura and Macarulla, where nab-paclitaxel serves as both a chemotherapeutic agent and an enabler of enhanced therapeutic efficacy through its delivery platform.

Liu *et al.* (2021)^162^ and Fu *et al.* (2021)^163^ contributed significantly to the development of novel nanoparticle-based chemotherapies with remarkable potential to address the challenges of non-specific biodistribution and drug resistance in cancer treatment, as well as metastatic recurrence. Liu *et al.* (2021) developed a highly innovative nanomedicine aimed at improving the therapeutic efficacy for breast cancer treatment.^162^ The approach involved copper-doped layered double hydroxide (Cu-LDH) nanoparticles, which were co-loaded with two FDA-approved anticancer agents—5-fluorouracil (5-FU) and albumin-bound paclitaxel (nAb-PTX). This combination offers synergistic chemotherapeutic actions, addressing limitations associated with single-modality therapies. The Cu-LDH nanoparticles were designed to be pH-sensitive, enabling an on-demand, heat-facilitated release of the drugs when exposed to mild 808 nm laser irradiation. This combination of photothermal therapy and chemotherapy demonstrated enhanced apoptosis induction in breast cancer cells, while minimizing side effects. The novel formulation exhibited excellent colloidal stability in saline and serum, as well as efficient tumor tissue accumulation. Importantly, this system dramatically reduced tumor size with significantly lower drug doses compared to other formulations, illustrating the potential for more cost-effective treatments with fewer adverse effects.

Fu *et al.* (2021) focused on the critical issue of metastasis and recurrence in cancer, which are the leading causes of cancer-related mortality.^163^ They developed a targeted nano-drug delivery system (NDDS) based on albumin nanoparticles modified with l-cysteine to carry hydrophobic paclitaxel. The self-assembled nanoparticles showed enhanced therapeutic efficacy against both tumor growth and lung metastasis, specifically in 4T1-bearing nude mice. The albumin nanoparticles effectively inhibited tumor growth and prevented the development of metastasis in a mouse model, offering a promising approach for tackling both primary tumors and metastatic disease. This work highlighted the potential of albumin nanoparticles as a delivery vehicle for hydrophobic drugs, demonstrating their ability to overcome solubility issues while providing effective chemotherapy with potential for metastasis prevention.

Li *et al.* (2024) explored a novel strategy to overcome the growing challenge of acquired resistance in lung adenocarcinoma (LUAD) patients receiving immune checkpoint inhibitors (ICBs), such as anti-PD1 therapies.^164^ Resistance to ICB therapy has been associated with activation of the Wnt/β-catenin signalling pathway, which hampers the efficacy of immunotherapies. To address this issue, Li *et al.* developed a supramolecular albumin system, named ABCA, which combined albumin with carnosic acid (CA), a Wnt pathway inhibitor. This system successfully suppressed the Wnt/β-catenin cascade *in vitro*, inhibiting cancer cell proliferation and promoting apoptosis.^164^*In vivo*, ABCA reactivated the anti-cancer immune response in immunotherapy-resistant LUAD models, significantly enhancing the efficacy of anti-PD1 treatment. This system's ability to overcome drug resistance and restore immune system sensitivity to ICB therapy, while minimizing adverse effects, presents a promising avenue for the clinical application of albumin-based nanomedicines in overcoming immunotherapy resistance.

Together, these studies showcase the immense potential of albumin-based nanoparticle systems, not only in improving traditional chemotherapy but also in enhancing immunotherapy outcomes. The unique characteristics of albumin, including its natural biocompatibility, stability, and ability to carry hydrophobic drugs, make it an ideal candidate for advanced nanoparticle formulations. These studies underscore the paradigm shift in chemotherapy that nanoparticle-based systems, like Abraxane® and other albumin-based formulations, represent, offering more targeted, effective, and less toxic therapies. The integration of multiple treatment modalities, such as chemotherapy, photothermal therapy, and immunotherapy, within a single nanoparticle-based system holds great promise for improving clinical outcomes, particularly in difficult-to-treat cancers with high metastatic potential.^165^

### 6.2 Advancements in engineering ferritin nanocages for tumor-targeted drug delivery and diagnostics

Advancements in engineering ferritin nanocages have revolutionized the field of drug delivery and diagnostics, particularly in the context of cancer treatment. Ferritin, a natural iron-storage protein, has a unique architecture that makes it an ideal candidate for nanocarrier development. Its spherical structure, self-assembling ability, and hollow interior enable it to encapsulate a variety of therapeutic agents, including chemotherapeutic drugs and imaging molecules. This versatility, combined with its biocompatibility and low immunogenicity, has positioned ferritin nanocages at the forefront of innovative nanomedicine.^73,166^

One of the most remarkable features of ferritin nanocages is their ability to be engineered for precise targeting of tumor tissues. By modifying the outer surface of the nanocages with tumor-specific ligands or peptides, researchers have enhanced their affinity for cancer cell receptors such as transferrin receptor 1 (TfR1). This receptor is overexpressed in many cancer cells, making it a valuable target for drug delivery.^167,168^ Moreover, advancements in the encapsulation of both hydrophilic and hydrophobic drugs within ferritin nanocages have addressed challenges associated with drug solubility and loading efficiency. Through innovative techniques such as the urea-dependent disassembly/reassembly process, researchers have successfully loaded hydrophobic drugs like camptothecin alongside hydrophilic agents like epirubicin. This dual-drug system has demonstrated synergistic therapeutic effects, with sequential drug release patterns optimizing treatment efficacy. The ability to control the release kinetics of encapsulated drugs, triggered by the acidic tumor microenvironment, further enhances the therapeutic potential of ferritin nanocages. This feature is particularly beneficial in targeting aggressive cancers such as gliomas and chemo-resistant tumors, where precise drug delivery is paramount.^169,170^

The diagnostic capabilities of ferritin nanocages add another dimension to their utility in cancer management. By integrating imaging agents within their structure, ferritin nanocages enable simultaneous therapy and diagnostics, a concept known as theranostics. *In vivo* imaging studies have shown that ferritin nanocarriers can efficiently cross the blood–brain barrier and accumulate in tumor tissues, providing real-time visualization of drug distribution and tumor response. This dual functionality not only improves treatment monitoring but also aids in early detection and personalized therapy planning.^171^

Economic and scalability considerations have also been addressed in recent advancements. The production of ferritin nanocages using cost-effective and scalable methods, such as plant-based expression systems, has made this technology more accessible for clinical applications. For example, the use of Nicotiana benthamiana plants to produce human ferritin heavy chains has significantly reduced production costs while maintaining high purity and functionality. This progress bridges the gap between laboratory research and real-world applications, paving the way for widespread clinical adoption.^172,173^

Furthermore, ferritin nanocages have demonstrated potential beyond traditional chemotherapeutics. Researchers have explored their use in delivering small cytotoxic proteins and novel biomolecules, broadening the scope of therapeutic possibilities. By engineering ferritin to recognize specific human receptors, these nanocarriers offer unparalleled precision in targeting cancer cells. This modularity, combined with their ability to encapsulate a diverse range of therapeutic agents, underscores their potential as a platform for advanced drug delivery systems.^174–176^

The advancements in engineering ferritin nanocages represent a significant leap forward in tumor-targeted drug delivery and diagnostics. The integration of precise targeting mechanisms, controlled drug release, and theranostic capabilities highlights their transformative potential in cancer nanomedicine. As research continues to refine their design and production, ferritin nanocages are poised to become a cornerstone technology in the fight against cancer, offering hope for more effective and personalized treatments.

For example, the study by Incocciati *et al.* (2023) presents a comprehensive investigation into the use of ferritin-based nanoparticles, particularly the HFt-W4 mutant, as a potential drug delivery system.^170^ The authors explore the efficient encapsulation of two chemotherapy agents, ellipticine and doxorubicin, in the ferritin protein cage, a strategy that is shown to enhance the loading capacity significantly. Specifically, they demonstrate that the HFt-W4 mutant can encapsulate an average of 98 ellipticine molecules, nearly an order of magnitude higher than the 8 molecules encapsulated by the wild-type HFt. This finding is further substantiated by the fluorescence contour maps, which indicate that the presence of ellipticine leads to quenching of the tryptophan fluorescence signal, a characteristic that confirms the successful encapsulation within the protein cavity. To verify that the encapsulation process does not interfere with the nanoparticle's functional properties, the study proceeds to assess the ability of the ellipticine-loaded HFt-W4 nanoparticle to bind to the transferrin receptor (TfR1), which is essential for targeted drug delivery.

The use of biolayer interferometry (BLI) in the study provides valuable real-time insights into the binding affinity of the HFt-W4 nanoparticles with TfR1. The experiment reveals that the ellipticine-loaded HFt-W4 retains its ability to bind to TfR1 with a dissociation constant (*K*_d_) of 25 nM, which is comparable to that of the wild-type HFt, further supporting the potential of this nanoparticle for targeted drug delivery. The authors also expand their study to include doxorubicin, a widely used chemotherapy drug, and demonstrate that the HFt-W4 mutant can encapsulate up to 41 molecules of this more hydrophilic drug, surpassing previous expectations for ferritin's ability to load such molecules. The study provides a valuable comparison with the encapsulation efficiency of HFt, which traditionally accommodates 20–30 molecules of doxorubicin.

The stability of these ferritin-based nanoparticles, particularly in the context of their use as drug carriers, is critically important for their effectiveness. To this end, the authors assess the stability of the ellipticine- and doxorubicin-loaded nanoparticles through high-performance size-exclusion chromatography (HP-SEC) and regular diafiltration. These analyses confirm that both types of nanoparticles remain stable for up to 30 days when stored at 4 °C, with no release of encapsulated drugs. This is a crucial finding as it suggests that the nanoparticles are capable of maintaining drug retention over extended periods, which enhances their practicality as controlled drug delivery systems. Furthermore, the study notes that the ferritin nanoparticles exhibited excellent stability even in cell culture media at 37 °C for up to 72 hours, indicating that they are well-suited for *in vitro* applications.

*In vitro* testing of the ferritin-based drug delivery system was conducted using HL60 promyelocytic cells, which overexpress the TfR1 receptor, making them a suitable model for evaluating targeted delivery. The study demonstrates that the encapsulated drugs, both ellipticine and doxorubicin, are efficiently delivered to the cells, as evidenced by the intracellular fluorescence observed after 24 hours of incubation. This fluorescence is attributed exclusively to the drugs carried by the ferritin nanoparticles, confirming that the observed cellular uptake is due to the drug-loaded nanoparticle and not to free drug molecules. In subsequent cytotoxicity assays, the MTT assay reveals that both ellipticine and doxorubicin, when encapsulated in HFt-W4, exhibit dose-dependent cytotoxic effects on HL60 cells, a finding that underscores the therapeutic potential of these ferritin-based nanoparticles.

**Fig. 15:**
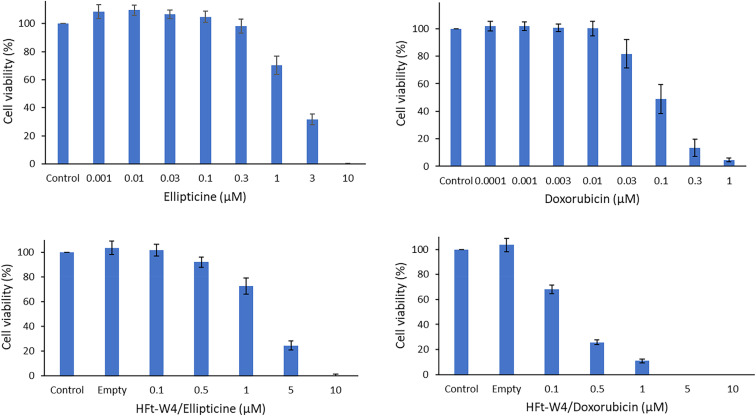
Viability of HL60 cells treated with free ellipticine/doxorubicin or with HFt-W4 loaded with ellipticine or doxorubicin (mean ± SD, *n* = 4). When conducting experiments with HFt-W4 loaded with ellipticine or doxorubicin, the specific concentration of the encapsulated drug was taken into account.^170^

The data presented in Fig. 15 illustrates the viability of HL60 cells treated with various concentrations of free ellipticine or doxorubicin, compared to cells treated with HFt-W4 loaded with these drugs. The results show that the drug-loaded nanoparticles significantly reduce cell viability, highlighting their potential as effective drug delivery systems. Importantly, the lack of cytotoxicity observed with empty ferritin at similar concentrations further supports the notion that the observed therapeutic effects are primarily due to the drug payload rather than the carrier itself. These findings collectively reinforce the promise of ferritin-based nanoparticles as targeted drug delivery vehicles, particularly in the context of cancer therapy.

Wang *et al.* (2022) provided an innovative perspective on the utilization of engineered ferritin nanocages as versatile drug carriers, particularly for anticancer applications.^73^ With their tumor-targeting capability, pH-responsive drug release, and high biocompatibility, ferritin nanocages hold immense promise in addressing critical challenges in drug delivery. However, the study acknowledged a significant limitation—low loading efficiency for hydrophobic drugs. To overcome this, the researchers redesigned the inner surface of ferritin drug carriers (ins-FDC) by integrating optimized hydrophobic peptides at the C-terminus of human H ferritin (HFn) subunits. By employing a urea-dependent disassembly/reassembly method and leveraging the nanocage's natural drug entry channels, both hydrophobic and hydrophilic drugs were efficiently encapsulated, as demonstrated by the successful loading of 39 hydrophobic Camptothecin (Cpt) and 150 hydrophilic Epirubicin (Epi) molecules per ins-FDC nanocage.

**Fig. 16:**
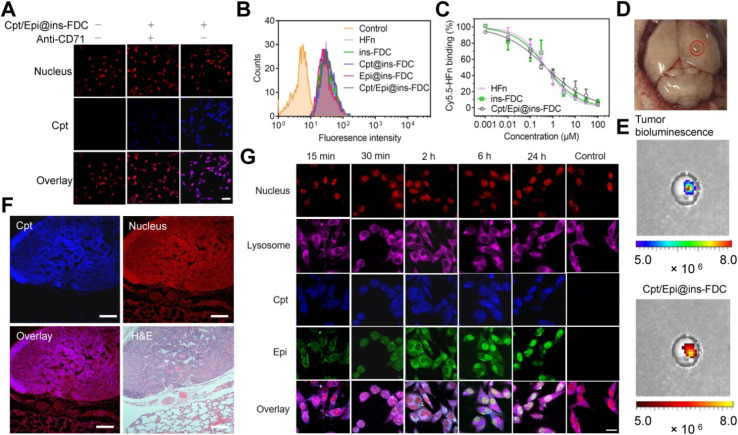
Tumor-targeting and cellular internalization of Cpt/Epi@ins-FDC. (A) Cpt/Epi@ins-FDC binds to CD71 receptors on tumor cells. Confocal laser scanning microscopy (CLSM) images of U87MG cells treated with Cpt/Epi@ins-FDC in the presence or absence of anti-CD71 monoclonal antibodies (mAbs). Red: propidium iodide (PI) (*λ*_ex_/*λ*_em_ = 535 nm/615 nm); blue: Cpt (*λ*_ex_/*λ*_em_ = 365 nm/500 nm). Scale bar: 20 μm. (B) Flow cytometry histograms showing the interaction of U87MG cells with different formulations after 2 hours of incubation. (C) Competitive binding assay comparing the binding efficiency of Cpt/Epi@ins-FDC, ins-FDC, and HFn. (D) Digital image of the brain from a U87MG tumor xenograft mouse model. (E) *In vivo* near-infrared fluorescence (NIRF) imaging demonstrating specific tumor accumulation of Cpt/Epi@ins-FDC following intravenous injection (*λ*_ex_/*λ*_em_ = 365 nm/500 nm). (F) Fluorescence staining using Cpt/Epi@ins-FDC and H&E staining of paraffin-embedded lung slices from HepG2 lung metastasis mouse models. Red: PI (*λ*_ex_/*λ*_em_ = 535 nm/615 nm); blue: Cpt (*λ*_ex_/*λ*_em_ = 365 nm/500 nm). Scale bar: 200 μm. (G) CLSM visualization of Cpt/Epi@ins-FDC internalization by U87MG cells and the sequential release of Cpt and Epi at specified time points. Purple: Cy5-labeled lysosomal-associated membrane protein 1 (LAMP1) (*λ*_ex_/*λ*_em_ = 650 nm/700 nm); red: PI (*λ*_ex_/*λ*_em_ = 535 nm/615 nm); blue: Cpt (*λ*_ex_/*λ*_em_ = 365 nm/500 nm); green: Epi (*λ*_ex_/*λ*_em_ = 485 nm/575 nm). Scale bar: 20 μm.^73^

The enhanced drug delivery system exhibited a programmed drug release pattern, improved stability, and biocompatibility of the encapsulated compounds. Importantly, the intrinsic CD71-binding ability of HFn facilitated tumor-specific targeting, enabling the nanocarrier to efficiently cross the blood–brain barrier (BBB) and accumulate in tumor tissues. Fig. 16 vividly illustrates these findings, showcasing how pre-treatment with an anti-CD71 antibody significantly inhibited the accumulation of Cpt/Epi@ins-FDC in tumor cells, confirming the specificity of the nanocarrier's interaction with CD71 receptors (Fig. 16A). Flow cytometry analyses further demonstrated that the drug-loading process did not compromise the tumor-targeting ability of the HFn nanocages (Fig. 16B and C). *In vivo* imaging of an orthotopic murine glioma model reinforced these results, with clear colocalization of fluorescence signals from the nanocarriers and bioluminescent tumor markers, confirming precise tumor targeting (Fig. 16D–F).

The study also explored the intracellular dynamics of the drug release mechanism. Cpt/Epi@ins-FDC rapidly adhered to tumor cell surfaces within minutes, and subsequent imaging revealed lysosomal localization of the nanocarriers, followed by drug release triggered by the acidic environment. The sequential release of hydrophilic Epi and hydrophobic Cpt ensured a sustained therapeutic effect, with Epi predominantly released into the nucleus at earlier stages and Cpt showing prolonged release over 24 hours (Fig. 16G). This design not only optimized drug release kinetics but also enhanced the therapeutic efficacy of the dual-drug system.

The dual-loaded ins-FDC nanocarriers exhibited potent synergistic cytotoxicity against cancer cells, including those resistant to conventional chemotherapy. The study reported significantly improved outcomes for glioma, metastatic liver cancer, and chemo-resistant breast tumors. Moreover, the nanocarriers demonstrated minimal systemic toxicity, as evidenced by high cell viability in non-cancerous human aortic smooth muscle cells (hASMC). Notably, colony formation assays and 3D spheroid models underscored the superior antitumor effects of Cpt/Epi@ins-FDC compared to single-drug formulations or free drugs. The engineered nanocages effectively reduced tumor spheroid volumes and inhibited clonogenic potential, highlighting their potential in advanced cancer therapies.

Wang *et al.*'s work exemplifies the transformative potential of engineered ferritin nanocages in theranostic applications, offering a platform for the co-delivery of hydrophobic and hydrophilic drugs while ensuring tumor specificity and controlled release.^73^ By integrating therapy and diagnostics, this research provides a robust framework for developing multifunctional nanocarriers for personalized cancer treatment.

Another striking example of the therapeutic application of ferritin nanoparticles is the work of Dong *et al.* (2022), which demonstrates the modification of ferritin nanocarriers with CendR peptides to overcome the limited permeability of ferritin in tumor tissues.^177^ By attaching tumor-penetrating peptides such as D(RGERPPR) to the outer surface of ferritin nanocages, Dong *et al.* achieved dual-targeting capabilities, enhancing both the uptake and cytotoxicity of paclitaxel-loaded ferritin nanoparticles in cancer cells expressing TfR1 and NRP-1 receptors. This study not only underscores the versatility of ferritin as a drug carrier but also highlights the importance of engineering its surface to optimize tumor penetration and therapeutic efficacy.

The encapsulation efficiency and tumor-targeting precision of ferritin-based systems were further advanced in the research by Chen *et al.* (2020), where doxorubicin, a potent yet cardiotoxic chemotherapeutic, was loaded into ferritin nanocages to mitigate off-target effects.^178^ The high biocompatibility and nanoscale uniformity of ferritin carriers enabled effective delivery of doxorubicin directly to tumor cells, reducing systemic toxicity and enhancing therapeutic outcomes. These findings resonate with Zhang *et al.*'s (2021) protocols,^176^ which provide detailed methodologies for loading chemotherapeutics like doxorubicin into ferritin nanocages, offering researchers a replicable framework for optimizing drug encapsulation and release kinetics.

Knödler *et al.* (2022) addressed the economic and scalability challenges associated with ferritin nanoparticle production.^173^ By developing a cost-effective method to produce human ferritin heavy chains in *Nicotiana benthamiana* plants, they significantly reduced production costs while maintaining high purity and functionality. These advance lays the groundwork for making ferritin nanomedicine more accessible for large-scale clinical applications, bridging the gap between experimental research and real-world use.

The therapeutic potential of ferritin nanocages extends beyond traditional chemotherapeutics, as highlighted by Palombarini *et al.* (2020).^168^ Their research explored the encapsulation of small cytotoxic proteins within ferritin nanocages, showcasing the modularity of ferritin as a drug carrier. By engineering ferritin to recognize specific human receptors, they created a delivery system capable of targeting cancer cells with high precision, paving the way for novel protein-based therapies.

Together, these studies illustrate the transformative potential of ferritin nanoparticles as a platform for combining anticancer drug delivery with imaging diagnostics. The synergistic integration of engineering innovations, biocompatibility enhancements, and cost-efficient production processes propels ferritin nanocages toward becoming a cornerstone in cancer nanomedicine.

### 6.3 Silk fibroin nanoparticles: a biodegradable platform for targeted cancer therapy

Silk fibroin nanoparticles (SFNPs) represent a highly versatile and promising platform in the field of drug delivery, particularly for cancer therapy. Derived from the natural silk protein produced by the silkworm *Bombyx mori*, silk fibroin (SF) is well-known for its exceptional biocompatibility, biodegradability, and mechanical robustness. The unique properties of SF, combined with its ability to form nanoparticles, make it an excellent candidate for the sustained delivery of chemotherapeutic agents, addressing several critical challenges in oncology.^179–181^

A significant advantage of SFNPs is their ability to deliver chemotherapeutic drugs in a controlled and sustained manner. By encapsulating therapeutic agents within their matrix, SFNPs allow for a gradual and steady release of the drug over time. This release profile is highly beneficial in reducing the frequency of drug administration, which can be burdensome for patients undergoing cancer treatment. Sustained drug delivery also helps maintain therapeutic drug levels within the target tissue, avoiding the peaks and troughs associated with conventional drug administration. This stability ensures that cancer cells are exposed to consistent concentrations of the drug, enhancing its cytotoxic effects on tumor cells while minimizing the potential for resistance development.^180,181^

Another critical benefit of SFNPs lies in their ability to reduce systemic toxicity. Many chemotherapeutic agents, while effective at killing cancer cells, also affect healthy tissues, leading to severe side effects such as nausea, hair loss, and immune suppression. SFNPs can be engineered to target tumor tissues specifically, leveraging the enhanced permeability and retention (EPR) effect commonly observed in cancerous tissues. Tumors often have leaky vasculature and poor lymphatic drainage, allowing nanoparticles like SFNPs to accumulate preferentially within the tumor microenvironment. Furthermore, SFNPs can be functionalized with targeting ligands such as antibodies, peptides, or aptamers, which bind specifically to receptors overexpressed on cancer cells, thereby enhancing their selectivity. This targeted delivery reduces off-target effects, sparing healthy tissues from unnecessary exposure to toxic chemotherapeutic agents and improving the overall quality of life for patients.^182,183^

The biodegradability of SFNPs is another vital feature that contributes to their therapeutic appeal. Once their role in drug delivery is fulfilled, SFNPs are broken down into amino acids by the body's natural enzymatic processes. These amino acids can be readily absorbed and utilized by the body, ensuring that SFNPs do not accumulate or cause long-term toxicity. This biodegradability is particularly advantageous in comparison to non-biodegradable materials, which can lead to complications such as inflammation or the need for surgical removal.^184^ Moreover, the structure of silk fibroin provides significant flexibility for modification and optimization. By altering the processing conditions, such as pH, temperature, or ionic strength, SFNPs can be tailored to achieve specific sizes, surface charges, and drug-loading capacities. These tunable properties are critical for optimizing drug delivery performance, as they influence factors such as circulation time, cellular uptake, and release kinetics. SFNPs can also encapsulate a wide range of chemotherapeutic agents, including hydrophobic drugs, which are often challenging to deliver using conventional systems. The hydrophobic regions of silk fibroin provide an ideal environment for encapsulating poorly water-soluble drugs, enhancing their bioavailability and therapeutic efficacy.^184,185^

In addition to their functional attributes, SFNPs exhibit excellent stability under physiological conditions. Unlike many synthetic polymers, which can degrade prematurely or release their payload unpredictably, SFNPs maintain their structural integrity in the bloodstream, ensuring that the encapsulated drug remains protected until it reaches the target site. This stability is particularly important for drugs that are sensitive to degradation or inactivation by enzymes, pH changes, or other factors in the biological environment.^185,186^

Beyond their role in drug delivery, SFNPs also offer opportunities for multifunctional applications in cancer therapy. By incorporating imaging agents or therapeutic molecules such as siRNA or immune modulators, SFNPs can serve as theranostic platforms, combining therapeutic and diagnostic capabilities within a single system. This integration enables real-time monitoring of drug delivery and treatment response, providing valuable insights for personalized medicine. Despite their numerous advantages, there are still challenges to address before SFNPs can achieve widespread clinical adoption. The scalability of SFNP production, the need for standardized protocols, and the cost of silk fibroin extraction and processing remain areas for further development. However, ongoing advancements in material science and nanotechnology are steadily overcoming these obstacles, paving the way for the broader application of SFNPs in cancer therapy.^186,187^

Silk fibroin nanoparticles present a biodegradable, biocompatible, and highly versatile platform for the sustained delivery of chemotherapeutic agents. Their unique ability to minimize systemic toxicity, enhance drug efficacy, and offer multifunctional capabilities highlights their transformative potential in the field of oncology. As ongoing research addresses existing challenges, SFNPs are poised to revolutionize cancer treatment and improve patient outcomes on a global scale.^188^ For example, the study by Pirota *et al.* (2023) demonstrated the innovative application of SFNPs engineered to encapsulate a cytotoxic compound, naphthalene diimide derivative (NDI-1), designed to interact with DNA secondary structures and disrupt the cancer cell cycle. Functionalizing SFNPs with cyclic pentapeptides containing the Arg-Gly-Asp (cRGD) sequence facilitated selective targeting of glioma cells, particularly U373 cell lines overexpressing integrin receptors such as ανβ3 and ανβ5.^188^ This targeted approach significantly reduced off-target effects, exemplifying the potential of SFNPs to enhance chemotherapy efficacy while mitigating adverse effects.

The study found that SFNs exhibited ideal characteristics for cellular uptake, including a nanometric size of less than 100 nm, a round shape with a smooth surface, and a negative zeta potential. These attributes facilitated effective internalization by cells. The encapsulation efficiency of NDI-1 within SFNs was notably high, ensuring that the active compound was not prematurely released but only after nanoparticle degradation within the cellular environment. Functionalization with cRGD further enhanced the selective uptake of SFNs by U373 cells, a glioma cell line with high integrin expression, as opposed to the D384 cell line, which exhibits lower integrin levels.

**Fig. 17:**
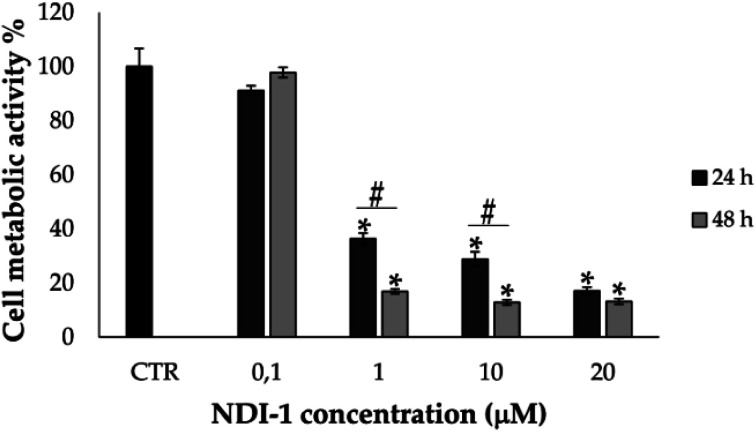
Cell metabolic activity % of U373 cells treated with increasing doses of NDI-1 for 24 and 48 h. Untreated cells at 24 hours were considered the control (CTR), representing 100% of metabolic activity. Multifactor ANOVA, mean values ± least significant difference (LSD), *n* = 4. *p < 0.01 with respect to CTR-; #*p* < 0.05.^188^

Fig. 17 highlights the dose-dependent cytotoxicity of NDI-1 on U373 cells over 24 and 48 hours. A concentration of 1 μM caused a significant reduction in cell metabolic activity, with a pronounced effect observed after 48 hours. These results were corroborated by further experiments on cRGD-functionalized SFNs. The functionalized nanoparticles demonstrated a marked cytotoxicity in U373 cells, with metabolic activity reduced to 22%, compared to 58% in D384 cells, as a result of the targeted delivery mechanism. This selectivity underscores the importance of cRGD functionalization, which enhances receptor-mediated uptake, even against the backdrop of electrostatic repulsion between the negatively charged nanoparticles and cell membranes.

Moreover, cytocompatibility assessments of unloaded SFNs revealed that both cRGD-functionalized and non-functionalized variants-maintained cell viability, with metabolic activity nearing 100%. This finding confirms the biocompatibility of silk fibroin as a nanoparticle material. However, upon loading with NDI-1, SFNs exhibited potent cytotoxic effects in both glioma cell lines. The enhanced selectivity provided by cRGD functionalization was attributed to the high expression of integrin receptors in U373 cells, which facilitated active targeting and improved therapeutic outcomes.

This study establishes a robust proof-of-concept for cRGD-functionalized silk fibroin nanoparticles as a targeted drug delivery system. The promising *in vitro* results, particularly the observed efficacy and reduced off-target cytotoxicity, pave the way for further *in vivo* investigations. The work exemplifies the role of biodegradable nanoparticles in oncology, offering sustained drug release, reduced systemic toxicity, and enhanced efficacy, as is characteristic of advanced protein-based nanocarriers like SFNs.

**Scheme 3:**
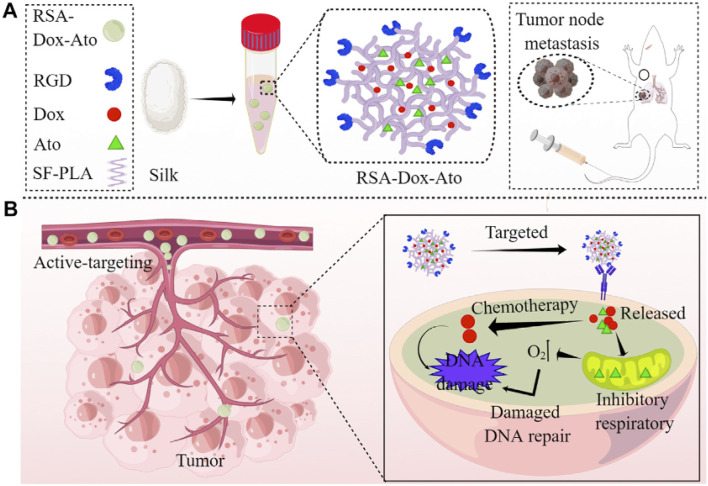
Schematics demonstration the RSA-Dox-Ato alleviating tumor hypoxia and improving the antitumor efficacy. (A) Scheme illustrating the synthesis of RSA-Dox-Ato NPs; (B) the mechanism of RSA-Dox-Ato NPs alleviate tumor hypoxia *in vivo*.^189^

Bin *et al.* (2022) demonstrated the application of SF-based nanocarriers in designing RSA-Dox-Ato nanoparticles, which integrate doxorubicin (Dox), atovaquone (Ato), and a tumor-targeting RGD peptide.^189^Scheme 3 illustrates the synthesis process and micellar-like structure of RSA-Dox-Ato NPs, emphasizing the functional role of the RGD peptide in promoting tumor-specific accumulation. These biopolymer nanoparticles exhibit tunable biodegradability, exceptional biocompatibility, and targeted drug delivery capabilities.

*In vitro* studies revealed that RSA-Dox-Ato NPs significantly enhance the cytotoxic effects on 4T1 cells compared to free Dox or RSA-Dox nanoparticles alone. The cytotoxicity assays, highlight the dose- and time-dependent inhibition of tumor cell viability. At a concentration of 1 μg mL^−1^, the RSA-Dox-Ato group exhibited a markedly lower cell survival rate (59.9%) compared to the RSA-Dox (62.1%) and Dox (82.6%) groups. This enhanced efficacy is attributed to the synergistic action of Dox and Ato, with the latter mitigating the hypoxic tumor microenvironment by inhibiting mitochondrial complex III activity, thereby increasing reactive oxygen species (ROS) levels.

The cellular uptake efficiency of the nanoparticles was assessed using fluorescence microscopy. The RSA-Dox NPs demonstrated superior endocytosis in 4T1 cells, underscoring the critical role of RGD in facilitating targeted drug internalization. Furthermore, the wound-healing assays demonstrated that RSA-Dox-Ato NPs effectively inhibited tumor cell migration, a key factor in cancer metastasis. This supports the notion that SF-based nanocarriers are capable of not only delivering chemotherapeutics but also suppressing tumor progression.

Intracellular ROS levels were evaluated to confirm the alleviation of hypoxia. The fluorescence imaging showed significantly higher ROS production in the RSA-Dox-Ato + Rosup group compared to other treatment groups, further validating the role of Ato in reversing the hypoxic tumor microenvironment. This alleviation enhances the susceptibility of tumor cells to chemotherapy and promotes apoptosis.

**Fig. 18:**
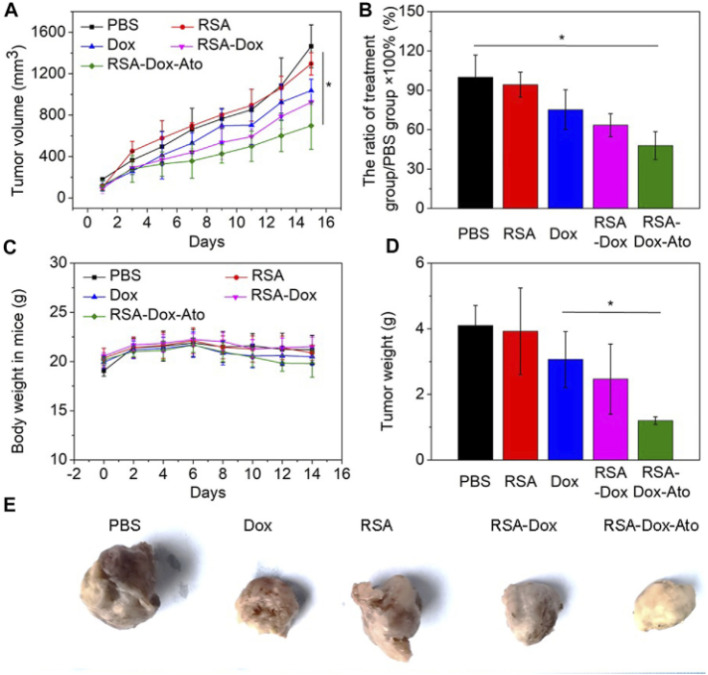
The behavior of RSA-Dox-Ato NPs post-i.v. injection *in vivo*. (A) The tumor volume growth curves of 4T1 tumor-bearing mice injected with nanodrugs for 14 days. The data were represented as the mean ± SD (*n* = 5, pp < 0.05, ppp < 0.01); (B) the tumor growth inhibition ratio of each group; (C) the body weight curve of 4T1 tumor-bearing mice upon the period of treatment; (D) the tumor weight curve of 4T1 tumor-bearing mice upon the period of treatment; (E) representative photos of tumor-bearing mice from different treatment groups at the end of the study.^189^

The *in vivo* antitumor efficacy of RSA-Dox-Ato NPs was assessed using a 4T1 tumor-bearing mouse model, with the results presented in Fig. 18. The tumor volume growth curves and tumor growth inhibition ratios demonstrate the superior performance of RSA-Dox-Ato nanoparticles compared to other formulations. These nanoparticles not only suppressed tumor growth effectively but also minimized off-target toxicity, as evidenced by the lack of adverse effects in non-tumor tissues.

Overall, the study by Bin *et al.* (2022) underscores the potential of silk fibroin nanoparticles as a biodegradable and biocompatible platform for the sustained delivery of chemotherapeutic agents.^189^ By targeting the hypoxic tumor microenvironment and improving drug accumulation at tumor sites, RSA-Dox-Ato nanoparticles exemplify an innovative strategy for enhancing the efficacy and safety of cancer therapies.

Pandey *et al.* (2020) explored silk fibroin nanoparticles as carriers for doxorubicin (DOX), a potent anti-cancer drug, demonstrating their potential for targeting glioblastoma cells.^190^ The nanoparticles were fabricated by a desolvation method and coated with Tween-80 to enhance their circulation time and facilitate drug delivery across the blood–brain barrier (BBB). The physicochemical characterization revealed that the particles were less than 150 nm in diameter, with a negative surface charge and an entrapment efficiency exceeding 85%. These nanoparticles exhibited sustained drug release profiles and enhanced cytotoxicity compared to free DOX, suggesting their effectiveness as drug delivery systems. Moreover, the nanoparticles induced no significant toxicity on their own, while their pro-inflammatory response, indicated by cytokine levels, was minimal. This study highlights the ability of silk fibroin nanoparticles to improve the efficacy of cancer treatment by enabling prolonged drug release and enhanced cellular uptake.

Horo *et al.* (2021) further demonstrated the potential of silk fibroin in drug delivery by developing doxorubicin-loaded chitosan–gold nanoparticles coated with functionalized silk fibroin.^191^ The nanoparticles exhibited a size of 8 ± 3 nm and were found to possess sustained drug release properties when coated with silk fibroin. These particles showed a significant improvement in cellular uptake in HeLa cells, with a seven-fold increase in uptake compared to uncoated particles. The cytotoxicity of the particles was also evaluated, with the silk fibroin-coated nanoparticles demonstrating a dose-dependent decrease in cell viability. These findings suggest that functionalized silk fibroin-coated materials can be highly effective for targeted and sustained drug release, offering a promising approach for cancer therapy. Furthermore, the study's live-cell imaging revealed the enhanced cellular uptake of the coated nanoparticles, indicating their potential for precise drug delivery to cancer cells.

Mitra *et al.* (2022) introduced a novel pH-induced self-assembly method to create silk fibroin nanoparticles with enhanced drug loading capacity and cellular uptake.^192^ The nanoparticles, which encapsulated rifampicin, exhibited a size of 165 ± 38 nm and displayed improved aerosol properties. These particles demonstrated selective endocytosis by macrophages, with higher uptake efficiency and sustained drug release in the presence of intracellular lysates. Additionally, the SFNs exhibited immunomodulatory effects by polarizing macrophages from the M1 phase to the M2 phase, highlighting their potential for both drug delivery and immune modulation. The enhanced antibacterial activity of the silk fibroin nanoparticles against *Mycobacterium smegmatis*-infected macrophages further emphasizes their dual function as both therapeutic agents and immune system modulators, making them a promising candidate for pulmonary drug delivery systems.

Pham *et al.* (2022) explored silk fibroin nanoparticles (FNPs) as a versatile oral drug delivery system for a range of pharmaceutical agents.^193^ The study tested FNPs loaded with various drugs, including ascorbic acid, dextromethorphan, paracetamol, and paclitaxel, which belong to different Biopharmaceutics Classification System (BCS) classes. The FNPs exhibited spherical nanosized particles (300–420 nm) with dominant silk-II structures and a negative zeta potential. These particles enhanced the solubility of class II and IV drugs and sustained their release for over 72 hours, while for class I and III drugs, the release was prolonged up to 6 hours. The silk fibroin nanoparticles also facilitated drug permeability across the intestinal membrane, improving cellular internalization and demonstrating their potential for oral drug delivery applications.

Hudita *et al.* (2021) focused on using silk fibroin-based nanoparticles as a strategy to reduce gastrointestinal (GI) toxicity associated with chemotherapy, specifically addressing 5-fluorouracil (5-FU)-induced GI mucositis.^194^ Silk fibroin nanoparticles were developed to efficiently deliver 5-FU to colorectal cancer cells, with the added benefit of protecting the intestines from the adverse effects of chemotherapy. *In vitro*, the nanoparticles exhibited significant antitumoral activity, inducing apoptosis and DNA fragmentation in cancer cells. *In vivo*, the nanoparticles effectively alleviated GI mucositis in a mouse model, providing a potential solution to one of the most common and debilitating side effects of chemotherapy. This study underscores the dual utility of silk fibroin nanoparticles in both enhancing cancer treatment efficacy and mitigating the side effects of chemotherapy.

Eivazzadeh-Keihan *et al.* (2021) synthesized a novel nanobiocomposite scaffold consisting of cross-linked pectin–cellulose hydrogel, silk fibroin, and magnesium hydroxide nanoparticles.^195^ This composite material demonstrated favorable cell viability and anti-biofilm properties, suggesting its potential for tissue engineering and infection prevention in cancer therapy. The presence of silk fibroin enhanced the scaffold's mechanical properties and biocompatibility, while magnesium hydroxide nanoparticles contributed to its antimicrobial activity. This study highlights the versatility of silk fibroin in creating multifunctional nanomaterials that can be used not only in drug delivery but also in tissue regeneration and infection control.

Wang *et al.* (2021) developed silk fibroin-based core–shell nanofibers for drug delivery and tissue engineering applications. The nanofibers, with a core of silk fibroin and a shell of poly(ε-caprolactone), exhibited excellent mechanical properties and biocompatibility.^196^ The incorporation of silk fibroin in the core of the nanofibers improved cell adhesion and proliferation, making them suitable for tissue engineering. Moreover, these nanofibers demonstrated controlled drug release, reducing the initial burst release of the drug and ensuring sustained delivery. This research highlights the potential of silk fibroin-based materials as scaffolds for tissue engineering and as drug carriers for cancer therapy.

Martínez *et al.* (2020) explored the use of silk fibroin nanoparticles for biodistribution studies by radiolabeling them with [^111^In] In and conjugating them with fluorescein isothiocyanate.^197^ These nanoparticles were characterized by dynamic light scattering and scanning electron microscopy, confirming their stability and suitable size for *in vivo* applications. The radiolabeled nanoparticles were injected into New Zealand rabbits, and scintigraphic imaging revealed that the nanoparticles were retained in the articular space for up to 24 hours. This study demonstrates the utility of silk fibroin nanoparticles in tracking drug delivery systems *in vivo*, offering valuable insights into their biodistribution and potential for cancer therapy.

Collectively, these studies highlight the multifaceted potential of silk fibroin nanoparticles in cancer therapy. Their biodegradability, biocompatibility, and ability to be functionalized with various agents make them promising candidates for targeted drug delivery, immune modulation, and tissue engineering. By modifying the surface of silk fibroin nanoparticles, researchers can optimize their properties to enhance therapeutic efficacy, reduce side effects, and improve patient outcomes in cancer treatment.

## 7 Public health impact of polymeric biodegradable nanoparticles in cancer therapy

The deployment of Polymeric Biodegradable Nanoparticles (PBNPs) in cancer therapy holds significant promise in transforming the landscape of cancer treatment, not only by advancing scientific understanding and improving clinical outcomes but also by making a considerable public health impact. The multifaceted benefits of PBNPs in cancer therapy go far beyond the immediate confines of medical research and clinical settings. They offer profound improvements in the treatment paradigm by targeting the specific needs of cancer patients, reducing the broader burden of the disease, and enhancing the quality of life for millions of individuals worldwide.^133,189^

One of the most substantial impacts that the use of PBNPs can have on public health is the potential for reduced cancer mortality. Cancer remains one of the leading causes of death globally, with millions of individuals diagnosed each year. Traditional cancer treatments, such as chemotherapy and radiation, have proven effective in many instances, but they often come with limitations, such as non-specific targeting of cancerous cells, systemic toxicity, and considerable side effects that severely compromise the patient's overall health. PBNPs, however, offer a more refined approach to therapy, particularly through their ability to encapsulate and deliver drugs specifically to tumor sites. This targeted drug delivery can lead to enhanced therapeutic efficacy, as the medication is concentrated in the areas that need it most, rather than being distributed throughout the entire body. By limiting off-target effects and enhancing the cytotoxicity at the cancer site, PBNPs can significantly increase the effectiveness of treatments, leading to higher survival rates. In cases where traditional therapies may have failed or been too toxic, the precision provided by PBNPs opens new doors to treatment, ultimately leading to improved outcomes for patients.^152,166^

The ability of PBNPs to specifically target tumor cells is closely tied to another critical benefit that these nanoparticles offer: minimized side effects. One of the most distressing aspects of cancer treatment, particularly chemotherapy, is the severe side effects that patients endure. Chemotherapy drugs, while potent against cancer cells, are also toxic to healthy cells in the body, particularly those in the gastrointestinal system, blood, hair follicles, and bone marrow. The systemic nature of these treatments leads to side effects such as nausea, vomiting, fatigue, hair loss, and immunosuppression, all of which significantly reduce the patient's quality of life.^189,198^ Additionally, the long-term toxicity of many chemotherapeutic agents may lead to organ damage or secondary cancers, further compounding the health challenges faced by cancer patients. PBNPs, with their ability to be engineered for specific targeting, reduce the exposure of non-cancerous tissues to the toxic effects of chemotherapy and other cancer drugs. By delivering the therapeutic agents directly to the cancer cells or tissues, PBNPs spare the surrounding healthy tissues, thereby minimizing collateral damage and reducing the occurrence of adverse effects. This targeted approach significantly improves the overall patient experience, not just by reducing physical discomfort but also by helping maintain a patient's functional capacity, leading to improved long-term outcomes and better overall well-being.^191,194^

In the context of public health, the accessibility and scalability of PBNP technology could play a pivotal role in ensuring that advanced cancer therapies are available to a larger portion of the global population. Currently, cancer therapies, particularly those involving expensive biologics and personalized medicine, are often limited to wealthier populations or those in developed countries with advanced healthcare systems.^125^ This disparity in access to treatment creates significant inequalities in cancer outcomes between different regions of the world. However, the scalability of PBNP production offers a potential solution to this problem. PBNPs can be synthesized using relatively simple and cost-effective methods, making them more affordable compared to traditional drug therapies or complex biologics. With improvements in production techniques, PBNPs could be manufactured on a large scale, significantly reducing the cost of cancer treatments. This reduction in cost would make these therapies more accessible to low- and middle-income countries, where cancer treatment options are often limited, thereby bridging the gap in healthcare disparity. Moreover, the ability to store and transport these nanoparticles efficiently means that they could be distributed to remote or underserved regions, ensuring that cancer patients in every corner of the world have access to the latest therapeutic options.^198,199^

Beyond economic considerations, the deployment of PBNPs could result in broader healthcare access in terms of treatment adaptability. Cancer is not a singular disease but a collection of over 100 different types, each with distinct molecular and genetic characteristics. Personalized medicine, which tailors' cancer treatment to the individual's specific genetic makeup and the molecular profile of their tumor, is one of the most promising frontiers in oncology. However, the high cost and complexity of many personalized treatment methods often make them inaccessible. PBNPs, with their versatility, could be adapted to a wide range of cancer types and patient profiles, offering an avenue for individualized therapies without the prohibitive costs associated with other personalized treatments.^200^ By adjusting the composition of PBNPs, such as the type of drug they carry, the surface modifications to enhance targeting, or their release kinetics, these nanoparticles can be tailored to suit the needs of each patient. This adaptability ensures that even in diverse healthcare settings, treatments can be optimized for efficacy, resulting in better overall outcomes for cancer patients across various populations.^198^

Another important facet of the public health impact of PBNPs is their potential to promote cancer prevention. Early detection and prevention of cancer are crucial to reducing the burden of the disease on society. Traditional cancer prevention methods, such as lifestyle changes and routine screenings, have been effective in some cancers but are not universally applicable or sufficient. The use of PBNPs in the delivery of vaccines or other prophylactic therapies could help in preventing the onset of certain cancers.^24^ For example, nanoparticles could be used to deliver targeted therapies that stimulate the immune system to recognize and destroy early-stage cancer cells before they can grow into full-blown malignancies. Additionally, PBNPs could play a role in delivering chemopreventive agents more effectively to reduce the risk of cancer development in high-risk individuals, such as those with a genetic predisposition to certain cancers. The preventive capacity of PBNPs could therefore shift the focus from reactive treatment to proactive disease management, which would significantly reduce cancer incidence and its associated healthcare costs.^21,24^

The transformative potential of PBNPs in cancer therapy is not just limited to individual patient outcomes or economic factors but also extends to broader public health strategies. By improving the efficacy and safety of cancer treatments, enhancing accessibility, and offering new avenues for cancer prevention, PBNPs contribute to the creation of a more robust and sustainable healthcare system. They provide a more equitable platform for cancer care, ensuring that innovations in treatment reach a global population, especially those in regions with limited access to advanced healthcare resources.^25^ This wide-reaching impact not only holds promise for reducing the global burden of cancer but also fosters a healthcare system that prioritizes patient-centered care and outcomes. With continued advancements in PBNP technology, we may witness a paradigm shift in how cancer is treated, ultimately leading to improved survival rates, better patient quality of life, and a more equitable distribution of healthcare resources worldwide. The incorporation of PBNPs in cancer therapy represents a new frontier in oncology that holds the potential to reshape the public health landscape, ushering in a future where cancer is not only treatable but preventable and accessible to all.^110^

## 8 Challenges and future directions

Despite the immense promise that Polymeric Biodegradable Nanoparticles (PBNPs) hold in revolutionizing cancer therapy, several significant challenges need to be addressed for them to reach their full potential in clinical and commercial applications. These challenges, which span the areas of production, safety, regulatory approval, and environmental sustainability, must be carefully navigated to ensure that PBNPs can be deployed safely, effectively, and responsibly on a global scale.

One of the most pressing challenges in the development and application of PBNPs is ensuring scalability and cost-effective manufacturing. The synthesis of PBNPs, while highly promising at the laboratory scale, often encounters significant obstacles when moving to industrial-scale production. Ensuring that PBNPs can be produced in large quantities without compromising the quality or functionality of the nanoparticles is critical to their widespread use in cancer therapy. Achieving consistent particle size, surface characteristics, and drug encapsulation efficiency across large batches is a complex task that requires robust manufacturing processes. Variations in production methods can lead to discrepancies in nanoparticle properties, which could affect the therapeutic outcome. Furthermore, the cost of raw materials, the complexity of the synthesis process, and the need for specialized equipment to produce PBNPs at scale could significantly increase the overall cost of the therapy. For PBNPs to become a viable treatment option globally, their production must be economically feasible, efficient, and scalable. This may require the development of new manufacturing techniques, such as high-throughput production systems, automation, or the use of alternative, less expensive materials. Ensuring that the production methods are both scalable and sustainable will be pivotal to meeting the growing demand for these therapies and facilitating their integration into global healthcare systems.

Safety and toxicity concerns are also among the major hurdles in the development of PBNPs for cancer therapy. While PBNPs offer the promise of more targeted and localized treatment with fewer side effects compared to traditional therapies, the long-term safety of these nanoparticles remains largely uncharted. The biocompatibility of PBNPs, especially when used *in vivo*, must be thoroughly evaluated to ensure that they do not induce any harmful effects, either acutely or over time. This includes assessing the potential for toxicity to normal cells, tissues, and organs, as well as the possible effects on the immune system. Even though the biodegradable nature of PBNPs suggests that they may pose fewer risks than non-biodegradable alternatives, there is still a need for extensive preclinical and clinical studies to evaluate their behavior in living organisms, including their biodegradation products, potential accumulation in organs, and long-term effects. The interactions between PBNPs and the immune system are particularly important, as nanoparticles can sometimes trigger unwanted immune responses, including inflammation, which could undermine the therapeutic benefits of the treatment. Furthermore, the potential for PBNPs to accumulate in vital organs, such as the liver or kidneys, and the effects of such accumulation on organ function and overall health need to be closely studied. Only through rigorous safety testing and clinical trials can the risks associated with PBNP-based therapies be properly understood and mitigated, ensuring that they are both effective and safe for patient use.

Regulatory hurdles also represent a significant barrier to the widespread adoption of PBNPs in clinical settings. Nanomedicine, including the use of PBNPs for cancer therapy, is subject to stringent regulatory oversight, but the approval process for nanoparticle-based therapies remains complex and often inconsistent. Regulatory agencies such as the U.S. Food and Drug Administration (FDA), the European Medicines Agency (EMA), and others around the world have established frameworks for evaluating the safety and efficacy of new drugs and medical devices, but there is still a lack of standardized protocols specifically for nanoparticle-based treatments. The unique properties of nanoparticles, such as their small size, high surface area, and ability to cross biological barriers, present challenges in terms of evaluating their safety, quality, and effectiveness. The existing regulatory frameworks for pharmaceuticals and medical devices were not designed with nanoparticles in mind, and as a result, they may not fully address the unique challenges posed by nanomedicine. For example, the evaluation of nanoparticle toxicity requires specialized testing methods to assess the nanoparticles' interactions with biological systems, as well as their potential for accumulation and long-term effects. Furthermore, the lack of standardized testing protocols for the characterization of PBNPs, such as their size distribution, surface charge, and drug loading capacity, could lead to delays in regulatory approval or inconsistent results across different studies. Developing clear, internationally accepted regulatory guidelines for PBNP-based therapies will be essential to accelerate their approval and adoption in clinical practice. These guidelines will need to address the specific challenges posed by nanoparticles, while ensuring that they are safe, effective, and high-quality products that meet the rigorous standards set by regulatory agencies.

In addition to these technical and regulatory challenges, environmental considerations are becoming an increasingly important factor in the development of PBNPs. As the use of nanoparticles in medicine and other fields continues to grow, there is a growing recognition of the need to assess the environmental impact of these materials. PBNPs are designed to be biodegradable, meaning they are expected to break down over time into non-toxic components that can be safely eliminated from the body. However, the biodegradation process, the nature of the byproducts, and their potential impact on ecosystems remain largely unexplored. For example, when PBNPs are excreted from the body, they could potentially enter water supplies or soil, where they may interact with organisms in the environment. Understanding the degradation products of PBNPs and their potential toxicity to aquatic life, soil microorganisms, or other components of the ecosystem is crucial for ensuring that these nanoparticles do not pose unforeseen risks to the environment. Research into the biodegradability and environmental fate of PBNPs must be conducted to assess their long-term impact and develop strategies to minimize any negative ecological effects. This is particularly important as the use of PBNPs expands, and large quantities of nanoparticles are introduced into healthcare systems, where they may eventually find their way into the environment. Sustainable production methods, responsible disposal practices, and the development of environmentally friendly materials will be critical to ensuring that the widespread use of PBNPs in cancer therapy does not result in harmful environmental consequences.

Despite these challenges, the future of PBNPs in cancer therapy holds great promise. Researchers are already making significant strides in addressing the scalability of production, improving safety profiles, and establishing clearer regulatory pathways. Technological innovations, such as automated manufacturing processes, improved nanoparticle characterization techniques, and novel methods of assessing biodegradability, are helping to overcome some of the current obstacles. Additionally, the growing awareness of environmental and safety concerns is driving the development of more sustainable, eco-friendly nanoparticle formulations that minimize toxicity and ensure safe disposal. Collaborative efforts between researchers, regulatory bodies, and industry stakeholders will be essential to creating a conducive environment for the successful translation of PBNP-based therapies from the lab to the clinic. With continued research, investment, and collaboration, PBNPs have the potential to revolutionize cancer treatment, providing safer, more effective, and more accessible therapies that could ultimately benefit patients around the world.

## 9 Conclusion

Protein-based nanoparticles (PBNPs) are emerging as a highly promising class of nanomaterials, with the potential to transform both antimicrobial and cancer therapies. Their distinctive characteristics, including biocompatibility, customizable functionality, and ease of modification, enable them to tackle significant challenges in modern medicine, such as antibiotic resistance and targeted cancer treatment. PBNPs operate through various mechanisms, including microbial membrane disruption, immune system modulation, biofilm inhibition, and targeted drug delivery, which enhances therapeutic effectiveness while minimizing adverse effects. Noteworthy examples, such as albumin, lactoferrin, and gelatin nanoparticles, along with peptide-based nanoparticles, demonstrate their broad applicability in both infection control and cancer therapy. Additionally, PBNPs show great promise in advancing cancer treatment through controlled drug release and tumor microenvironment modulation, providing opportunities for precision medicine. However, challenges remain, particularly with respect to scaling production, ensuring long-term safety, navigating regulatory processes, and addressing environmental impacts. Overcoming these challenges is essential for the broader clinical implementation of PBNPs. With continued research and technological progress, the potential of PBNPs to improve outcomes in both infectious diseases and cancer therapy is highly promising, offering groundbreaking solutions for global public health challenges.

## Data availability

This study is a review article, and no new data were generated or analyzed during the course of this research. All data discussed and referenced are available in the publicly accessible sources cited within the article. Further information can be provided upon reasonable request from the corresponding author.

## Conflicts of interest

On behalf of all authors, the corresponding author states that there is no conflict of interest.
